# Disulfiram metabolite Cu(DDC)_2_ enhances radionuclide uptake *in vivo* revealing insights into tumoural ablation resistance

**DOI:** 10.1016/j.ebiom.2026.106165

**Published:** 2026-02-11

**Authors:** Katie Brookes, Jessica S. Fear, Caitlin E.M. Thornton, Ling Zha, Jana Kim, Benjamin Small, Sarinya Wongsanit, Hannah R. Nieto, Holly Adcock, Adam Jones, Truc T. Pham, Giovanni Bottegoni, Liam R. Cox, Vinodh Kannappan, Weiguang Wang, Caroline M. Gorvin, Daniel G. Stover, Christine Spitzweg, Sissy Jhiang, Matthew D. Ringel, Moray J. Campbell, Kavitha Sunassee, Philip J. Blower, Kristien Boelaert, Vicki E. Smith, Martin L. Read, Christopher J. McCabe

**Affiliations:** a Department of Metabolism and Systems Science, College of Medicine and Health, University of Birmingham, Birmingham, UK; b School of Biomedical Engineering & Imaging Sciences, King's College London, London, UK; c Research Institute in Healthcare Science, Faculty of Science and Engineering, University of Wolverhampton, Wolverhampton, UK; d Disulfican Ltd, University of Wolverhampton Science Park, Wolverhampton, UK; e School of Chemistry, University of Birmingham, Birmingham, UK; f Institute of Clinical Sciences, University of Birmingham, Birmingham, UK; g Università degli Studi di Urbino Carlo Bo, Urbino, Italy; h Division of Endocrinology, Diabetes, and Metabolism and Cancer Biology Program, The Ohio State University College of Medicine and Comprehensive Cancer Center, Columbus, OH, USA; i Department of Internal Medicine IV, University Hospital of Munich, LMU Munich, Munich, Germany; j Division Endocrinology, Diabetes, Metabolism and Nutrition, Mayo Clinic, Rochester, MN, USA; k Division of Cancer Biology, Cedars Sinai Cancer, Los Angeles, USA; l Department of Applied Health Sciences, College of Medicine and Health, University of Birmingham, Birmingham, UK

**Keywords:** Thyroid cancer, Breast cancer, NIS, VCP, Proteostasis, Transcription

## Abstract

**Background:**

Exploitation of the sodium iodide symporter (NIS) has potentially broad clinical application across different tumour ablative settings but often fails in aggressive cancer due to diminished transport activity. We aimed to discover whether enhancing NIS function by modulating proteostasis was targetable *in vivo*, as well as the clinical relevance to radioiodide (RAI) treatment of patients with cancer.

**Methods:**

We used 3D modelling, iterative design, reformulation, RAI uptake, RNA-Seq, cell surface biotinylation assays and NanoBRET in transformed cell lines and primary thyroid cells from patients to identify new drugs targeted at enhancing NIS function and to uncover their respective mechanisms. Systemic drug responses were monitored via ^99m^Tc pertechnetate gamma counting and SPECT/CT imaging in wild-type BALB/c and Tg-rtTA/tetO-BRAF^V600E^ mice, as well as orthotopic NOD.Cg-Prkdc^scid^ Il2rg^tm1Wjl^/SzJ (NSG) breast cancer.

**Findings:**

Copper diethyldithiocarbamate (Cu(DDC)_2_), a metabolite of the FDA-approved drug disulfiram, modulated NIS function in thyroid and breast cancer cells (*P* < 0.05). Mechanistically, Cu(DDC)_2_ elicited a dual effect on NIS function, targeting valosin containing protein (VCP)—a key regulator of proteostasis—as well as inducing potent transcriptional responses (*P* < 0.05). In mice, the copper-bound metabolite stimulated NIS activity in normal thyroid tissue, thyroid tumours and in breast orthotopic tumours (*P* < 0.05), the latter augmented by the histone deacetylase inhibitor vorinostat (SAHA). Notably, there was clinical association of drug-perturbed genes in RAI-treated thyroid cancer, enabling construction of a robust dual risk score classifier for predicting recurrence (AUC >0.95; *P* < 0.001).

**Interpretation:**

Our findings reveal a mechanistic pathway towards enhancing radionuclide uptake *in vivo*, with clinical relevance for RAI therapy and identifying survival indicators of recurrent disease.

**Funding:**

This work was funded by the U.S. 10.13039/100000005Department of Defense (BC201532P1), 10.13039/501100000265Medical Research Council (CiC/1001505 and MR/Z504828/1), 10.13039/501100000884British Thyroid Foundation (1002175). We further acknowledge support from the 10.13039/100010269Wellcome Trust and EPSRC funded Centre for Medical Engineering at King’s College London (203148/Z/16/Z), the Wellcome Multiuser Equipment Radioanalytical Facility (212885/Z/18/Z), and the EPSRC programme for Next Generation Molecular Imaging and Therapy with Radionuclides (EP/S019901/1).

**Research in context**

**Evidence before this study**

β-emitting radioiodide is a widely used and effective treatment to destroy thyroid cancer cells post-surgery and for targeting metastases. Resistance to radioiodide treatment in aggressive disease reflects the diminished activity of the sodium iodide symporter (NIS), the sole transporter responsible for cellular iodide uptake at the plasma membrane. Oncogenically dysregulated cellular processes (e.g. proteostasis, transcription) outside of canonical signalling pathways are emerging as critical targets to enhance NIS function, but their translational relevance has not been defined *in vivo*.

**Added value of this study**

We pursued four distinct strategies to identify drugs with translatable potential to improve NIS function *in vivo*. We found that the disulfiram metabolite Cu(DDC)_2_ stimulates radionuclide uptake in nine *in vitro* human model systems (thyroid and breast) and three *in vivo* preclinical mouse models. Detailed mechanistic studies revealed Cu(DDC)_2_ as a dual agonist targeting proteostasis and transcriptional pathways to enhance NIS activity. From these insights, we established a survival indicator based on clinically relevant transcription factors and proteostasis genes linked with recurrence and radioiodide resistance.

**Implications of all the available evidence**

Our study identifies a drug approach capable of enhancing NIS activity *in vivo*, thereby providing a basis for further therapeutic studies aimed at tumour ablation in the thyroid and other NIS-expressing cancers such as breast.

## Introduction

The unique ability of the sodium iodide symporter (NIS) to transport iodide and related radionuclides is critical in a wide array of therapeutic and imaging settings.^1^ This includes cellular ablation in cancer and hyperthyroidism, whole body imaging of tumours and metastases, tracking oncolytic viruses and CAR-T cells, ablative NIS delivery in tumours, and ongoing clinical trials in multiple current tumour settings (NCT05081492, NCT06508463, NCT05346484).^2^, ^3^, ^4^, ^5^, ^6^, ^7^ The harnessing of NIS activity, however, often fails or is sub-optimal *in vivo*. Radioiodide treatment for thyroid cancer, for instance, is hampered by diminished NIS expression in subsets of patients, particularly those with aggressive and metastatic disease.^8^ NIS activity is further compromised by its non-functional intracellular location, away from the plasma membrane (PM), which contributes to the failure of ^131^I as a therapeutic option in NIS-expressing cancers such as breast.^9^, ^10^, ^11^

Combinatorial targeting of the MAPK pathways which generally drive tumourigenesis in thyroid cancer (BRAF and MEK inhibitors) has shown recent promise in overcoming radioiodine refractoriness, although there are issues of drug resistance, adverse events and a lack of randomised trials.^12^, ^13^, ^14^, ^15^, ^16^ New approaches are thus urgently required to manipulate NIS function *in vivo*, and hence enhance radioiodide uptake and tumour/metastatic ablation.

We recently identified a panel of FDA-approved compounds which significantly induced radioiodide uptake,^17^ including the proteasomal inhibitor disulfiram.^18^^,^^19^ The use of mass spectrometry to identify new interacting proteins of NIS also previously led us to identify the Endoplasmic Reticulum Associated Degradation (ERAD) protein p97/VCP as being critical to NIS protein processing.^20^ A critical cofactor of VCP is NPL4, and disulfiram was recently shown to inhibit NPL4 activity via its copper bound diethyldithiocarbamate metabolite Cu(DDC)_2_.^21^ We hence hypothesised that disulfiram increases radioiodide uptake by transiently interfering with ERAD via a VCP/NPL4 pathway, permitting more NIS protein to be trafficked to the plasma membrane.

A second FDA-approved candidate drug in our study was the anti-fungal agent clotrimazole, which has been identified as a re-purposed small molecule capable of allosterically inhibiting VCP activity.^17^^,^^20^^,^^22^ Importantly, combining clotrimazole or similar VCP inhibitors (VCPi) with drugs targeting different cellular process, such as the histone deacetylase (HDAC) inhibitor SAHA, had potent effects on NIS activity both in thyroid and breast cells *in vitro*.^17^ However, although well tolerated, clotrimazole is poorly bioavailable.^22^

In the current study we conjectured that disulfiram and clotrimazole might represent key starting points for developing drugs with translatable potential to enhance radioiodide uptake *in vivo*. As part of an extensive rational design and reformulation drug screen, we show that while multiple clotrimazole analogues enhanced radioiodide uptake *in vitro*, the disulfiram metabolite Cu(DDC)_2_ was more tractable *in vivo*. Contrary to its reported cardinal function of inhibiting NPL4,^21^ we identify that Cu(DDC)_2_ acts as a dual agonist to modulate NIS function, impacting both transcriptional and proteostatic pathways *in vivo*, and generating insight into predicting tumour recurrence risk in radioiodide-treated patients. Critically, based on these unique findings, combinatorial approaches induced significant tumoural radionuclide uptake into orthotopic murine breast cancers. We thus reveal mechanistic understanding of how to manipulate NIS function *in vivo* to enhance radionuclide uptake.

## Methods

### Ethics

This study was conducted according to the Declaration of Helsinki ethical guidelines and collection of normal human thyroid tissue was approved by the Local Research Ethics Committee (Birmingham Clinical Research Office, Birmingham, UK; reference number 17-291). No age/sex information was available or race/ethnicity categories considered as subjects were anonymised and tissue collected as excess to surgery as part of our ethics agreement. Informed written consent was obtained from each subject.

All animal experiments were conducted in compliance with the ARRIVE guidelines and performed in accordance with the Animals (Scientific Procedures) Act, 1986 with protocols approved by UK Home Office (PP5976272 and PP9982297) and animal welfare and ethical review body for King's College London (St Thomas' Campus).

### Animal experiments

Mice were housed in ventilated cages (n = 2 to 4 per cage) under controlled temperature (23 ± 2 °C), humidity (40–60%) and light/dark cycles lasting 12 h/day. Animals received dust-free bedding and nesting material, with ad libitum access to chow and autoclaved water. Male wild-type BALB/c mice (8–10 weeks of age, n = 6–11 animals/group, Charles River) received Cu(DDC)_2_-albumin nanoparticles (3–5 mg/kg) or control vehicle (saline) by IP or IV injection on days 1 and 3 of experiment. On day 4, mice were anaesthetised by isoflurane inhalation (3%, Animalcare, York, in O_2_) and maintained under isoflurane anaesthesia during IV administration of ^99m^Tc-pertechnetate (^99m^TcO4^-^, 0.5 MBq). After 30 min, mice were culled by anaesthetic overdose and tissues harvested. Thyroid glands were removed using a dissecting microscope and radioactivity measured by gamma counting (1282 Compugamma, LKB Wallac). Male Tg-rtTA/tetO-BRAF^V600E^ mice^23^ (8–10 weeks of age, n = 3–6 animals/group) received Cu(DDC)_2_-albumin nanoparticles (5 mg/kg) or control vehicle (saline) by IV injection. Thyroid tumours were established by feeding Tg-rtTA/tetO-BRAF^V600E^ mice on a doxycycline (DOX) chow diet (2.5 g/kg, Inotiv) for 7 days (total animals used = 21). Control mice were fed on normal chow (TD.97184, Inotiv). CB-5083 (15 or 25 mg/kg/day versus control vehicle) was given to male wild-type BALB/c mice (8–10 weeks of age, n = 3 animals/group, Charles River, total animals used = 9) by oral gavage administration for 4 days prior to IV administration of ^99m^TcO4^−^.

MDA-MB-231 xenografts were established in 6-week-old immunodeficient female NOD.Cg-Prkdc^scid^ Il2rg^tm1Wjl^/SzJ (NSG) mice (Charles River) by injecting 1 × 10^6^ cells into the mammary fat pad while mice were under anaesthesia. When MDA-MB-231 tumours reached 89 ± 16 mm^3^ (day 26 post tumour inoculation) mice (n = 3/group) were administered with Cu(DDC)_2_-albumin (5 mg/kg/day; IV) + SAHA (100 mg/kg/day; IP) or control vehicle. Cu(DDC)_2_ was administered 1 h after SAHA. On day 4, mice were maintained under isoflurane anaesthesia during IV administration of ^99m^TcO4^-^ (10 MBq) and imaged by SPECT/computed tomography (CT) (NanoSPECT, Mediso).

Mice were randomly assigned to treatment and control groups before data collection using a computer-generated random sequence in Randomice.^24^ Total number of animals used in study was 75. Sample size calculation was initially informed by effect sizes and variability from our prior study using the same tracer and wild-type BALB/c mice.^25^ In that dataset, the drug combination SAHA and chloroquine produced a mean increase of 362 units relative to vehicle (vehicle mean 687 ± 146; drug mean 1049 ± 311), corresponding to a large effect size (Cohen's d ≈ 1.5). Power calculations (α = 0.05, 80% power) indicated that at least 5 mice per group would be required for this study. Our study with the drug CB-5083 was exploratory and the sample size was selected to use the minimum number of animals necessary while allowing detection of biologically meaningful differences.

In our orthotopic breast cancer model we used the minimum number of mice necessary to achieve the study's objectives which is in accordance with the 3Rs (Replacement, Reduction, Refinement) ethos. We used SPECT/CT imaging as it gives highly quantitative and reproducible measurements, resulting in lower inter-animal variability, further supporting our sample size calculation with a group size of 3 mice being considered as appropriate and ethically justified.^26^ Data points were excluded only if due to technical errors or outside of quality control thresholds. Measurements were conducted at consistent times of day to avoid circadian-related variability.

### Drugs and in silico modelling

VCP inhibitors CB-5083 and CB-5399 were kindly provided by Cleave Therapeutics (San Francisco, USA). Cu(DDC)_2_-albumin was kindly provided by Disulfican (University of Wolverhampton), while compounds C21 to C24 were synthesised by SIA Enamine (Latvia) and dissolved in DMSO. Details on the preparation of Cu(DDC)_2_-albumin are available (https://patents.google.com/patent/EP3459526A1/en). Modified clotrimazole compounds (C1–C20, C25) were synthesised using standard procedures (School of Chemistry, University of Birmingham) and resuspended in DMSO. Predicted binding of clotrimazole to an allosteric binding site in VCP was modelled using Schrödinger (Schrodinger, Inc.–New York) and 3D-coordinates from the cryo-EM resolved structure of VCP in complex with allosteric VCP inhibitor UPCDC30245 (PDB: 5FTJ).^27^ All drugs were diluted in RPMI-1640 medium (1:100; Life Technologies) prior to cell treatment. For IP and IV administration in mice, nanoencapsulated Cu(DDC)_2_ was formulated in saline. CB-5083 was resuspended in 0.5% methyl cellulose (400cp/water) using a mortar and pestle and vortexed prior to oral gavage administration. SAHA was formulated in 5% DMSO, 40% PEG400, 5% Tween-80 and saline prior to IP administration.

### Tissue and cell culture

Primary thyrocytes were isolated and cultured as previously described.^17^ Thyroid, breast and bone marrow mesenchymal stem cell lines (TPC-1, 8505C, SW1736, BCPAP, SUM52, ZR751, AU565, L87-NIS) were maintained in RPMI-1640 (Life Technologies),^28^, ^29^, ^30^ while cervical cancer HeLa, embryonic kidney HEK293 and breast cell lines MCF7 and MDA-MB-231 were maintained in DMEM (Sigma–Aldrich). SK-BR-3 cell line was maintained in McCoy's 5A modified media. All media was supplemented with 10% fetal bovine serum (FBS), penicillin (10^5^ U/l), and streptomycin (100 mg/l) and cell lines maintained at 37 °C and 5% CO_2_ in a humidified environment. Cell lines were kindly provided by Rebecca Schweppe (TPC-1, SW1736 and BCPAP, University of Colorado), Clare Davies (AU565 and ZR751, University of Birmingham) and John Heath (SUM52, University of Birmingham).^31^, ^32^, ^33^ Sources of other cell lines are also given (Supplementary Information). Cells were cultured at low passage (limit of 10 passages to avoid overpassaging), authenticated by short tandem repeat (STR) analysis (NorthGene, Biofortuna) and tested for mycoplasma contamination (EZ-PCR kit; Geneflow; latest test─2/2025). STR analysis was conducted using 8 loci allowing identical matches and approximate power of discrimination of 1 in 1,000,000,000. Cell lines were considered to be related with a matching percentage >80% (Supplementary Figure S1).

### Nucleic acids and transfection

VCP cDNA was cloned into pcDNA3.1 with an added N-terminal LgBiT tag, whereas a SmBiT tag was added to the C-Terminal of NIS cDNA as previously described.^17^ NIS-NanoLuc (Nluc) cDNA was synthesised and subcloned into pcDNA3.1 by GeneArt (ThermoFisher Scientific). Nevin Lambert (Georgia Regents University) kindly provided the NanoBRET plasma membrane marker KRAS-Venus, as well as the Venus-tagged markers RAB5 and RAB11. Venus-tagged markers RAB1 and RAB8 were kindly provided by Kevin Pfleger (University of Western Australia).^34^ Plasmid DNA and siRNA transfections were performed with TransIT-LT1 (Mirus Bio) and Lipofectamine RNAiMAX (ThermoFisher Scientific) in accordance with the manufacturers’ guidelines.

### Western blotting and cell surface biotinylation assays

Western blotting and cell surface biotinylation assays (CSBA) were performed as described previously.^17^^,^^25^^,^^35^ Blots were probed with specific antibodies against Na,K-ATPase (1:1000; Cell Signalling Technology), NIS (1:1000; Proteintech), NPL4 (1:500; Cell Signalling Technology), VCP (1:1000; Cell Signalling Technology) and β-actin (1:10000; Sigma–Aldrich). HRP-conjugated secondary antibodies (Agilent Technologies) against mouse or rabbit IgG were used at 1:2000 dilution.

### RNA-seq and TaqMan qPCR

Total RNA was extracted using the RNeasy Micro Kit (Qiagen) and reverse transcribed using the Reverse Transcription System (Promega). Mouse thyroid tissue was homogenised in buffer RLT using TissueLyser II (Qiagen; 2 × 2 min cycles; 30 Hz) and 5 mm stainless steel beads. Expression of specific mRNAs was determined using the 7500 Real-time PCR system (Applied Biosystems) as previously.^17^ TaqMan qPCR assays are listed (Supplementary Information). RNA sequencing (RNA-Seq) was performed in 8505C cells treated with Cu(DDC)_2_ (250 nM, 24 h) versus vehicle. RNA-Seq libraries were prepared at the Next Generation Sequencing Facility (Genomics Birmingham Genomics Service, University of Birmingham). Unless stated, all analyses was undertaken using the R platform for statistical computing (version 4.1.3) and the indicated library packages implemented in Bioconductor (release 3.14), ensuring consistent package versions across all analyses. FASTQC files were QC processed, adaptors trimmed (Trimmomatic) and aligned to GRCh38 (Rsubread).^36^ The percentage of reads aligning to rRNA was consistently below 3%. On average, more than 19,000 transcripts were detected per sample with at least one mapped read. Transcript abundance estimates were normalised and differentially expressed genes (DEGs) identified using a standard pipeline (TMM normalisation, logPV >0.69 & |log_2_FC| > 0.37), and functional classification (DAVID, ToppGene) performed.

### NanoBiT, NanoBRET and ROS-Glo cell assays

Cells were seeded in 6-well plates at a density of 3.5 × 10^5^ cells per well and transfected with plasmid DNA (e.g. 50 ng pcDNA3.1-NIS-Nluc and 500 ng pcDNA3.1-KRAS-Venus). 24 h post-transfection, cells were harvested and reseeded into white 96-well plates in phenol-red-free DMEM (Life Technologies). Furimazine (Promega) was added to each well in accordance with the manufacturer's guidelines and readings taken at 120 s intervals for up to 30 min (PHERAstar FS microplate reader; BMG Labtech). In some experiments, cells were treated with CB-5339 or Cu(DDC)_2_ prior to addition of furimazine. NanoBRET signal was calculated using standard protocols by dividing the acceptor emission at 535 nm by the donor emission at 475 nm before normalising to background signal. ROS levels were measured using the ROS-Glo™ H_2_O_2_ Assay (Promega) in accordance with the manufacturers' guidelines.

### Radioiodide uptake assays

Cells were seeded in 24-well plates and then treated with drugs and/or transfected with siRNA. Cells were then incubated for 1 h with 0.05 μCi iodine-125 (^125^I) (Hartmann Analytic; Folkestone, UK) at a final concentration of 10^−7^ mol/l NaI. Following incubation, cells were washed twice in HBSS (Sigma–Aldrich) to remove unincorporated ^125^I and lysed in 100 μl 2% SDS. Radioiodide uptake was determined using the Berthold Gamma Counter (Berthold) to measure gamma radiation (counts per minute). Protein concentrations were determined using the Pierce™ BCA colorimetric assay (ThermoFisher Scientific). ^125^I uptake relative to protein concentration was calculated in picomoles of ^125^I/μg protein, according to the following equation: Picomoles of ^125^I/μg protein = [5000∗(Counts/Total Protein)]/120000.

### Immunofluorescence and immunohistochemistry

24 h post transfection, cells were washed with PBS and fixed for 15 min at RT in 4% paraformaldehyde/PBS. After rinsing in PBS and 0.1 M glycine/PBS, cells were permeabilised in 0.1% saponin buffer. Incubation with a mixture of primary antibodies [mouse-anti-HA (1:100) and rabbit-anti-NIS (1:100)] was performed at RT for 1 h. Cells were rinsed three times with saponin buffer before a 1 h incubation with secondary antibodies (Alexa-Fluor-555-conjugated goat anti-rabbit or Alexa-Fluor-488-conjuated goat anti-mouse). Finally, cells were rinsed with saponin buffer (3×) and PBS (1×) and mounted onto slides using Prolong Gold anti-fade reagent with DAPI (Molecular Probes). Cells were viewed and images captured using 100× objective on a LSM 880 Airyscan confocal microscope. Images were analysed using FIJI software.

Mouse tissues fixed in 10% formalin were paraffin-embedded, sectioned into 5 μm slices and stained with haematoxylin and eosin (H&E) (Cellular Pathology Service, University of Birmingham). Mouse thyroid specimens were immunostained with a specific rabbit monoclonal antibody against Ki67 (1:10,000) using standard protocols (Cellular Pathology Service, University of Birmingham). Immunostained sections were counterstained with Mayer's haematoxylin.

### TCGA, GEO and transcription factor datasets

Gene expression data and clinical information for papillary thyroid cancer (PTC) were downloaded from cBioPortal (cbioportal.org/), FireBrowse (firebrowse.org), NCI Genomic Data Commons (GDC; portal.gdc.cancer.gov/) and the Gene Expression Omnibus (GEO).^37^, ^38^, ^39^ RNA-Seq data for 501 TCGA THCA and 59 normal thyroid samples were analysed (Broad GDAC Firehose, https://doi.org/10.7908/C11G0KM9). Normalised gene expression values were transformed as X = log_2_ (X+1) where X represents the normalised fragments per kilobase transcript per million mapped reads (FPKM) values. Differential gene expression analysis was also performed using the GEO2R interactive web tool in GEO^40^ for analysis of the GEO dataset GSE33630.^41^^,^^42^ Heatmaps were constructed and hierarchical cluster analysis performed using Morpheus (Broad Institute; https://software.broadinstitute.org/morpheus). Functional gene classification of RNA-Seq data was performed using DAVID^43^^,^^44^ and ToppGene.^45^ A manually curated list of transcription factors in the human genome (n = 1639) was downloaded from https://humantfs.ccbr.utoronto.ca/.^46^

### LASSO regression and patient survival characteristics

Receiver operating characteristic (ROC) curves were plotted and cut-off values calculated in IBM SPSS Statistics (Version 29). Patients were stratified into high and low expression groups for 337 Cu(DDC)_2_-associated transcription factor (TF) genes, and disease-free survival (DFS) characteristics determined [i.e. Kaplan–Meier (log-rank test), and univariate Cox regression analyses]. The machine learning algorithm LASSO was used to identify predictors of recurrence in the most clinically relevant TF genes (i.e. n = 20–63, DFS, *q* < 0.05, adjusted using the Benjamini-Hochberg FDR correction procedure). TF gene riskscore classifiers (13, 16 and 22 genes) were constructed based on genes with non-zero coefficient values. In addition, a 22 gene dual TF + VCP/proteostasis riskscore classifier was constructed based on coefficient values derived from LASSO regression of 35 candidate genes [i.e. 22 TF and 13 VCP/proteostasis genes]. A combined riskscore for each patient was calculated according to the equation: riskscore = ∑coefficient value ∗ expression (FPKM). Subsequent ROC curve analysis was performed for each multigene riskscore classifier and patients stratified into high and low risk of recurrence using cut-off values calculated as above. Patient DFS characteristics [i.e. Kaplan–Meier (log-rank test), univariate and multivariate analyses] were determined based on multigene riskscore classifiers. Multivariate models were adjusted to control for potential confounding with the following covariates: age, sex, disease stage and ATA risk group. Further details on development of risk prediction models are given (Supplementary Information).

### Statistics

Statistical analyses were performed using IBM SPSS Statistics (Version 29), GraphPad Prism (Version 10.1), XLSTAT and Microsoft Excel. All results were obtained from triplicate biological experiments unless otherwise indicated. Normality of data was assessed using the Shapiro–Wilk test. *P*-values > 0.05 indicated that the data did not significantly deviate from normality. For comparison between two groups, data were subjected to the Student's t-test, and for multiple comparisons one-way ANOVA was used with either Dunnett's or Tukey's post-hoc test. Kruskal–Wallis and Spearman's correlation tests were performed on non-parametric data. *P*-values were adjusted using the Benjamini-Hochberg FDR correction procedure to correct for multiple comparisons. Dunn's multiple comparison post-hoc testing was used after Kruskal–Wallis tests to determine significance between datasets in groups of 3 or more. Non-parametric methods were used to analyse TCGA data (Shapiro–Wilk test; *P* < 0.001). Fisher's exact test was used to determine the significance of nonrandom associations between two categorical variables. *P* < 0.05 was considered significant. All *P*-values reported were two-sided.

### Role of funders

The funders had no role in study design, data collection and analysis, decision to publish, or preparation of the manuscript.

## Results

### Drug screening identifies modulators of NIS activity

**Fig. 1:**
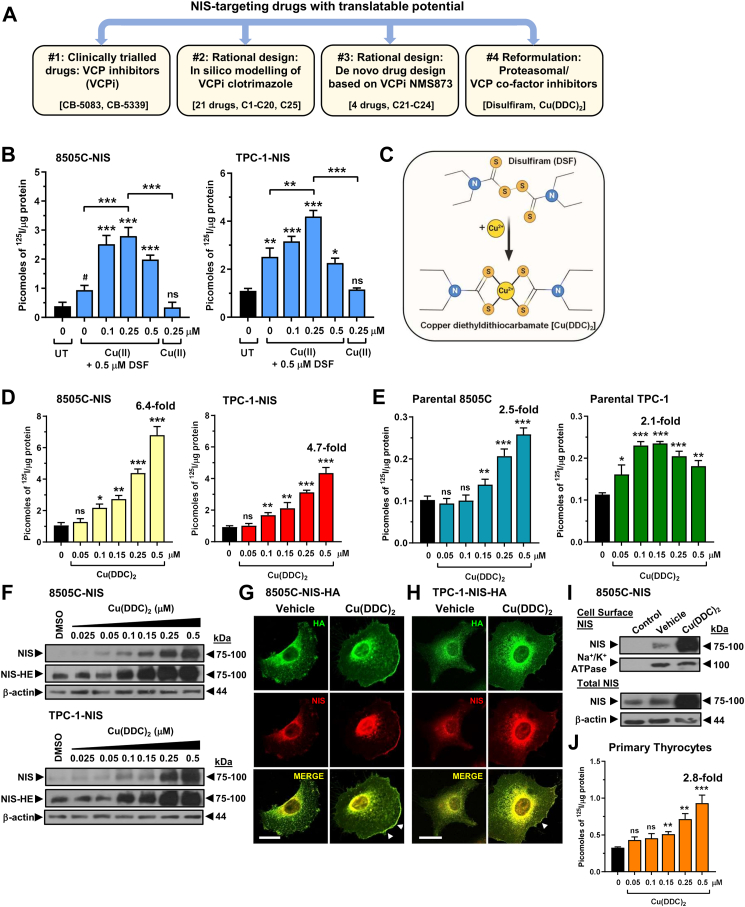
**Copper-bound metabolite of disulfiram augments NIS activity and radioiodide u**ptake. (A) Overview of rational design and reformulation drug strategies used in study. **(B)** RAI uptake of copper gluconate [Cu(II)]-treated 8505C–NIS and TPC-1-NIS cells alone or in combination with disulfiram (DSF) versus untreated (UT). **(C)** Schematic illustrating metabolic conversion of DSF to copper diethyldithiocarbamate [Cu(DDC)_2_]. **(D)** RAI uptake of Cu(DDC)_2_-treated 8505C–NIS and TPC-1-NIS cells. **(E)** Same as (D) but using parental 8505C and TPC-1 cells. **(F)** Western blot analysis of NIS expression in Cu(DDC)_2_-treated 8505C–NIS and TPC-1-NIS cells; HE, higher exposure. **(G** and **H)** Confocal imaging of 8505C–NIS-HA (G) and TPC-1-NIS-HA (H) cells treated with Cu(DDC)_2_ or vehicle (DMSO). Confocal images represent HA expression (green), NIS expression (red), and a merged image (yellow). Arrows (white) indicate regions of greater NIS plasma membrane localisation; HA, haemagglutinin. Scale bar: 20 μm. **(I)** Western blot analysis of NIS protein levels at the PM relative to Na+/K + ATPase following the cell-surface biotinylation assay (CSBA) in 8505C–NIS cells after Cu(DDC)_2_ treatment. Control: Biotin tag omitted (*upper*), total protein before biotin separation (*lower*). **(J)** RAI uptake of Cu(DDC)_2_-treated human primary thyrocytes. Data presented as mean ± SEM (n = 3); one-way ANOVA, Dunnett's or Tukey's post hoc test (ns, not significant; ^∗^*P* < 0.05, ^∗∗^*P* < 0.01, ^∗∗∗^*P* < 0.001); unpaired two-tailed t-test (^#^*P* < 0.05).

To identify drugs with translatable potential to improve NIS function *in vivo* we undertook 4 drug development approaches (Fig. 1A). First, clinically trialled VCP inhibitors CB-5083 and CB-5339 induced RAI (^125^I) uptake in NIS-expressing cells (Supplementary Figure S2A and B), as well as in human primary thyrocytes (Supplementary Figure S2C), but did not induce thyroidal uptake of radionuclides in BALB/c mice (Supplementary Figure S2D).

Second, based on our previous data that allosteric inhibition of VCP via the drug clotrimazole is a promising alternative route to drug NIS activity (Supplementary Figure S3A),^20^ we used computational iterative drug design to construct 21 analogues with improved bioavailability (Supplementary Figure S3B–G, Supplementary Table S1). Third, we constructed 4 additional compounds with greater predicted VCP binding based on the cryo-EM structure of VCPi NMS783 (Supplementary Figure S4A and B).^47^ However, when tested alone or in combination with SAHA, the total 25 clotrimazole analogues gave only modest improvements in RAI uptake (Supplementary Figures S3D, 4C and 4D).^17^^,^^25^ We therefore confined subsequent experiments to our fourth approach: reformulation of the proteasomal inhibitor disulfiram targeting NIS degradation.

### Copper-bound metabolite targets NIS activity in thyroid cancer and human primary cells

Interestingly, copper gluconate augmented disulfiram's potentiation of RAI (^125^I) uptake in NIS-expressing TPC-1 and 8505C cells *in vitro* (Fig. 1B), whereas copper gluconate by itself had no effect (Fig. 1B, Supplementary Figure S5A). Disulfiram is metabolised to diethyldithiocarbamate (DDC) *in vivo*, a hydrophilic and highly polar intermediatory which readily binds elemental copper to form the DDC-metal complex Cu(DDC)_2_ (Fig. 1C). Skrott et al. reported that Cu(DDC)_2_ binds and inhibits VCP via its co-factor NPL4.^21^ We therefore addressed the hypothesis that inhibition of VCP via its critical co-factor NPL4 might represent an attractive strategy to target NIS function *in vivo*.

A significant finding was that Cu(DDC)_2_ markedly increased RAI uptake across multiple parental and stable NIS-expressing thyroid cancer cell lines in a dose-dependent manner (> 2-fold, Fig. 1D and E, Supplementary Figure S5B), accompanied by parallel increases in NIS but not VCP protein (Fig. 1F, Supplementary Figure S5C–G), as well as enhanced NIS localisation at the plasma membrane (PM) (Fig. 1G–I, Supplementary Figure S6A–E). Stable NIS cells were used to enhance the sensitivity of assay detection and accentuate the impact of Cu(DDC)_2_ on post-translational mechanisms. Control experiments showed that the methylated and inactive metabolite of disulfiram (MeDDC), lacking the copper ion, did not induce RAI uptake (Supplementary Figure S6F). Critically, Cu(DDC)_2_ was also active in human primary thyrocytes (Fig. 1J), indicating that it impacts endogenous NIS activity, rather than merely overcoming neoplastic repression of NIS function.

**Fig. 2:**
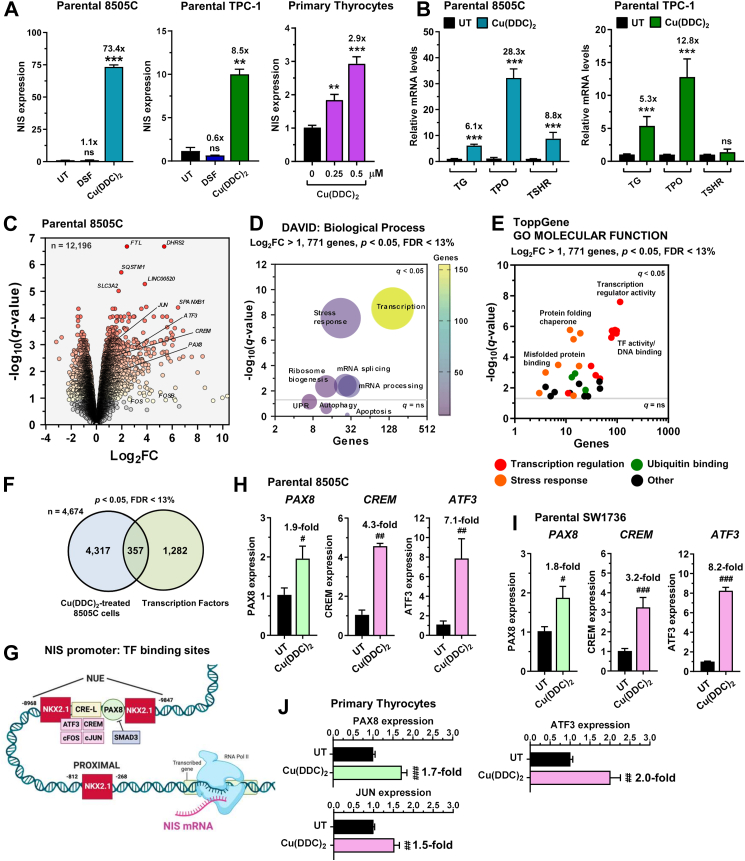
**Robust transcriptional response induced by copper-bound metabolite in thyroid cells**. (A) Relative NIS mRNA in parental 8505C and TPC-1 cells treated with DSF or Cu(DDC)_2_, as well as in human primary thyrocytes treated with Cu(DDC)_2_ at indicated doses. **(B)** Relative TG, TPO and TSHR mRNA levels in Cu(DDC)_2_-treated parental 8505C and TPC-1 cells. **(C)** Volcano plot comparing log_2_FC with q-value (-log base 10) for RNA-Seq analysis (n = 12,196 genes) of parental 8505C cells treated with Cu(DDC)_2_ versus vehicle. **(D** and **E)** DAVID: biological process (D) and ToppGene: GO molecular function (E) classification of 771 differentially expressed genes (DEGs; log_2_FC > 1, *P* < 0.05, FDR <13%). **(F)** Venn diagram showing overlap in DEGs (n = 4674, *P* < 0.05, FDR < 13%) versus human transcription factors (TF). **(G)** Schematic illustrating the relative position of TF binding sites in the human NIS promoter. Created with BioRender.com. **(H** and **I)** Relative PAX8, CREM and ATF3 mRNA in parental 8505C (H) and SW1736 cells (I) treated with Cu(DDC)_2_ versus untreated (UT). **(J)** Relative PAX8, ATF3 and JUN mRNA in human primary thyrocytes treated with Cu(DDC)_2_ versus UT. Data presented as mean ± SEM (n = 3), one-way ANOVA, Dunnett's post hoc test (ns, not significant, ^∗∗^*P* < 0.01, ^∗∗∗^*P* < 0.001), unpaired two-tailed t-test (^#^*P* < 0.05, ^##^*P* < 0.01, ^###^*P* < 0.001).

As Cu(DDC)_2_ treatment was associated with increased NIS protein we assessed the effect of Cu(DDC)_2_ on NIS transcription and noted a marked impact on NIS mRNA levels in parental and stable NIS-expressing TPC-1 and 8505C cells, as well as in human primary thyrocytes (Fig. 2A, Supplementary Figure S6G). In contrast, disulfiram had a less pronounced and inconsistent impact on NIS mRNA. Unexpectedly, Cu(DDC)_2_ also increased expression of the thyroid genes thyroglobulin (*TG*) and thyroid peroxidase (*TPO*, Fig. 2B), which are central to thyroid hormone biosynthesis, and whose expression is generally strongly repressed in thyroid cancer.

### Dissecting the mechanistic impact of targeting NIS in thyroid cells

As disulfiram's anticancer capability has been reported to include elevating reactive oxygen species (ROS) levels,^18^^,^^48^^,^^49^ we assessed whether Cu(DDC)_2_ induces significant ROS in thyroid cells. Whereas copper gluconate induced ROS levels, Cu(DDC)_2_ and Cu(II) + disulfiram had no effect (Supplementary Figure S7A). As copper gluconate itself did not increase cellular RAI uptake (Supplementary Figure S5A), it is therefore unlikely that Cu(DDC)_2_'s mechanism of action primarily involves modulating ROS, which is known to impact NIS function.^50^

Given the marked transcriptional changes induced by Cu(DDC)_2_ in thyroid cells, and the lack of any impact on ROS, we next performed RNA-Seq analysis to identify transcriptional pathways altered by Cu(DDC)_2_ (Fig. 2C). Functional classification confirmed strong drug:gene associations with VCP and proteasomal inhibitors including disulfiram, as well as copper-based drugs (Supplementary Figure S7B, Supplementary Table S2). Importantly, Cu(DDC)_2_ was associated with potent transcriptional changes (Fig. 2D and E, Supplementary Figure S7C), including significant dysregulation (*P* < 0.05, FDR < 13%) of 21.8% of genes (357/1639) encoding transcription factors in the human genome (Fig. 2F).^46^ Within this we identified transcription factors upregulated by Cu(DDC)_2_ with well-characterised functional binding sites in the NIS promoter (Fig. 2G),^51^ including PAX8 (*P* = 8.9 × 10^−4^), CREM (*P* = 1.3 × 10^−4^), ATF3 (*P* = 2.7 × 10^−5^) and JUN (*P* = 1.5 × 10^−5^). TaqMan qPCR confirmed Cu(DDC)_2_-induced transcription factor expression in multiple cell types, including human primary thyrocytes (Fig. 2H–J, Supplementary Figure S7D–F). Thus, apart from its described role as an inhibitor of the VCP co-factor NPL4,^21^ we now identify a role for Cu(DDC)_2_ as a regulator of NIS transcription.

### Copper-bound metabolite acts as a dual agonist to enhance NIS activity

**Fig. 3:**
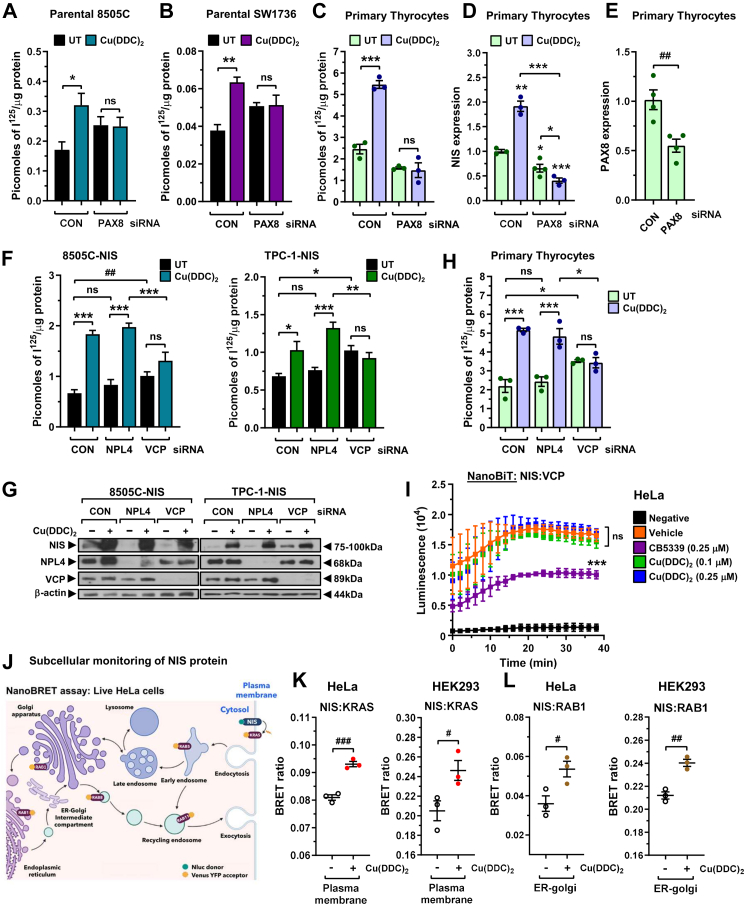
**Dual agonist effect of Cu(DDC)_2_ enhances NIS activity**. (A**–**C) RAI uptake in parental 8505C cells (A), parental SW1736 cells (B) and human primary thyrocytes (C) following PAX8-siRNA depletion and Cu(DDC)_2_ treatment. CON: scrambled control siRNA. **(D)** Relative NIS mRNA in human primary thyrocytes following PAX8-siRNA depletion and Cu(DDC)_2_ treatment. **(E)** Relative PAX8 mRNA in human primary thyrocytes following PAX8-siRNA depletion. **(F)** RAI uptake in 8505C–NIS and TPC-1-NIS cells following NPL4-or VCP-siRNA depletion and Cu(DDC)_2_ treatment. **(G)** Western blot analysis of NIS, NPL4 and VCP in 8505C–NIS and TPC-1-NIS cells after NPL4-or VCP-siRNA depletion. **(H)** Same as (F) but in human primary thyrocytes. **(I)** NanoBiT evaluation of protein: protein interaction between NIS and VCP in living HeLa cells treated with CB5339 or Cu(DDC)_2_ versus controls. **(J)** Schematic illustrating NanoBRET assay to monitor proximity of NIS with plasma membrane protein KRAS, as well as subcellular markers RAB5 (early endosome) and RAB11 (recycling endosome). Created with BioRender.com. Modified from Read ML et al. Clinical Cancer Research, 2024.^25^**(K** and **L)** NanoBRET evaluation of NIS localisation at the PM (K, KRAS) or in ER-golgi (L, RAB1) in live HeLa and HEK293 cells treated with Cu(DDC)_2_. Data presented as mean ± SEM (n ≥ 3), one-way ANOVA, Dunnett's or Tukey's post hoc test (ns, not significant, ^∗^*P* < 0.05, ^∗∗^*P* < 0.01, ^∗∗∗^*P* < 0.001), unpaired two-tailed t-test (^#^*P* < 0.05, ^##^*P* < 0.01, ^###^*P* < 0.001).

To challenge the hypothesis that Cu(DDC)_2_ has a dual effect on NIS function, we first ablated PAX8, a master regulator of the thyroid differentiated phenotype.^52^ Critically, Cu(DDC)_2_ was unable to induce RAI uptake or NIS mRNA expression when PAX8 was depleted (Fig. 3A–E). In contrast to PAX8, ablation of CREM had no impact on Cu(DDC)_2_-induced RAI uptake in multiple thyroid cell types (Supplementary Figure S8A–C).

Surprisingly, when we abrogated VCP and NPL4, Cu(DDC)_2_ retained activity in the absence of NPL4 but not VCP (Fig. 3F–H, Supplementary Figure S8D). Thus, despite being an inhibitor of NPL4,^21^ Cu(DDC)_2_ required functional VCP but not NPL4 expression to enhance RAI uptake. Subsequent NanoBiT assays in live HeLa and HEK293 cells revealed that Cu(DDC)_2_ did not impact the stringency of NIS:VCP binding, thus implying a role as a non-canonical VCP inhibitor (Fig. 3I, Supplementary Figure S8E and F).

We next performed NanoBRET assays in live cells to determine whether Cu(DDC)_2_ impacts NIS subcellular localisation—the cardinal determinant of its symporter function—independently of transcriptional regulation. Utilising KRAS tagged with Venus-YFP, Cu(DDC)_2_ was associated with increased NIS localisation at the PM (Fig. 3J and K).^53^ We also detected greater NIS protein in the endoplasmic reticulum (ER) to cis-golgi location (RAB1) but not at other subcellular locations (Fig. 3L, Supplementary Figure S8G and H). Collectively, these data suggest that Cu(DDC)_2_ has a dual effect on NIS function, enhancing PAX8 induction of NIS expression, whilst inhibiting VCP function independently of NPL4 to impact subsequent NIS protein processing and cellular localisation.

### Reformulated Cu(DDC)_2_ induces NIS activity and transcriptional responses *in vivo*

**Fig. 4:**
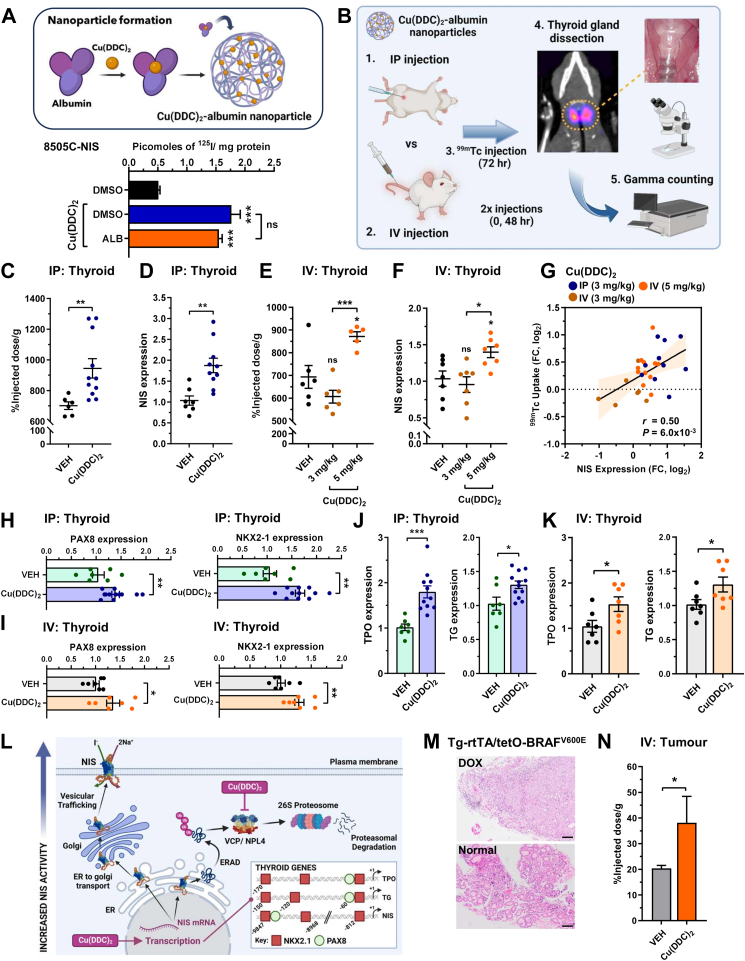
**Copper-bound metabolite stimulates NIS activity to enhance radionuclide uptake *in vivo***. **(A)** Schematic illustrating albumin nanoencapsulation of Cu(DDC)_2_. *Lower*: RAI uptake in 8505C–NIS cells treated with Cu(DDC)_2_ in DMSO or albumin nanoencapsulated (ALB). **(B)** Overview of *in vivo* study to investigate the effect of Cu(DDC)_2_ given by IP (step 1) or IV routes (step 2) on thyroidal NIS function in WT BALB/c mice. **(C** and **D)**^99m^TcO4^-^ uptake (C, n = 6–11) and relative NIS mRNA (D) in thyroid glands from Cu(DDC)_2_-ALB treated WT BALB/c mice given by IP route. Total animals used = 18). **(E** and **F)** Same as (C and D) but Cu(DDC)_2_-ALB given by IV route at indicated doses (n = 5–7). Total animals used = 21. (**G**) Pearson correlation analysis between thyroidal ^99m^TcO4^-^ uptake (FC, log_2_) and relative NIS mRNA (FC, log_2_) in Cu(DDC)_2_-ALB treated WT BALB/c mice as outlined (B). 95% CI (upper/lower) are shown. **(H** and **I)** Relative PAX8 and NKX2-1 mRNA in thyroids from Cu(DDC)_2_-ALB treated WT BALB/c mice given by IP (H) or IV (I) routes. **(J** and **K)** Same as (H and I) but relative TPO and TG mRNA. **(L)** Schematic illustrating the dual impact of Cu(DDC)_2_ on NIS function to enhance RAI uptake by inducing NIS mRNA and inhibiting VCP activity. *Inset*—promoter/enhancer regions of *TPO*, *TG* and *NIS* genes with relative positions of NKX2-1 and PAX8 binding sites. **(M)** Representative H&E stained images of thyroid tissue from Tg-rtTA/tetO-BRAF^V600E^ mice fed with DOX (*upper*) versus normal (*lower*) chow for 7 days. Scale bars, 100 μM. **(N)**^99m^TcO4^-^ uptake in thyroid tissue in DOX chow fed Tg-rtTA/tetO-BRAF^V600E^ mice treated with Cu(DDC)_2_-ALB given by IV route (n = 3–6). Total animals used = 9. Data presented as mean ± SEM, unpaired two-tailed t-test (ns, not significant, ^∗^*P* < 0.05, ^∗∗^*P* < 0.01, ^∗∗∗^*P* < 0.001). Images created with BioRender.com.

Cu(DDC)_2_ has relatively poor *in vivo* solubility, limiting its potential clinical utility.^54^ We thus reformulated the drug with albumin to enhance its solubility (Fig. 4A). Importantly, Cu(DDC)_2_ nanoencapsulated with albumin retained the ability to stimulate RAI uptake in comparison to non-encapsulated drugs (Fig. 4A, Supplementary Figure S8I), enabling progression to *in vivo* experiments (Fig. 4B), especially in murine models in which the core physiological role of NIS to mediate sodium-dependent iodide uptake into thyroid cells is conserved with humans.

In wild-type BALB/c mice, intraperitoneal (IP) administration of albumin nano-encapsulated Cu(DDC)_2_ significantly induced thyroidal uptake of the radiotracer technetium-99m (^99m^TcO4^-^) (Fig. 4C, n = 11, 3 mg/kg dose) compared to vehicle (n = 6), with thyroidal NIS mRNA levels increased by almost 2-fold (Fig. 4D). Likewise, intravenous (IV) injection of nano-encapsulated Cu(DDC)_2_ at a dose of 5 mg/kg increased thyroidal ^99m^TcO4^-^ uptake (Fig. 4E, n = 5), again accompanied by elevated thyroidal NIS mRNA (Fig. 4F). In contrast, there were no notable differences in ^99m^TcO4^-^ uptake in other major tissues (Supplementary Figure S9A). Importantly, there was a positive correlation between thyroidal ^99m^TcO4^-^ uptake and NIS mRNA levels (r_s_ = 0.503) in Cu(DDC)_2_-treated mice (Fig. 4G). Thyroid transcription factors PAX8 and NKX2-1—as well as the thyroid genes TPO and TG—were also induced in thyroids of Cu(DDC)_2_-treated mice compared to controls (Fig. 4H–K, Supplementary Figure S9B), further implicating a common mechanism for transcriptional upregulation of thyroid-specific genes (Fig. 4L).

We next progressed to Tg-rtTA/tetO-BRAF^V600E^ mice to examine the translatable potential of Cu(DDC)_2_ to improve NIS function in thyroid tumours (Fig. 4M, Supplementary Figure S9C and D), which had diminished expression of thyroid genes (Supplementary Figure S9E) and elevated proliferative activity (Supplementary Figure S9F and G). Importantly, treatment with Cu(DDC)_2_ (Supplementary Figure S9H) led to a significant increase (1.9 ± 0.5-fold, *P* < 0.05) in tumour uptake of ^99m^TcO4- after 30 min (Fig. 4N, n = 3, 5 mg/kg dose) versus vehicle (n = 6), but not in salivary glands or liver (Supplementary Figure S9I), as well as no differences in body weight (Supplementary Figure S9J).

Combined, our data therefore demonstrate that Cu(DDC)_2_ significantly targets and stimulates NIS activity in normal and tumourous thyroid glands *in vivo*.

### Clinically relevant drug-associated genes improve risk stratification of radioiodine-treated cancer

**Fig. 5:**
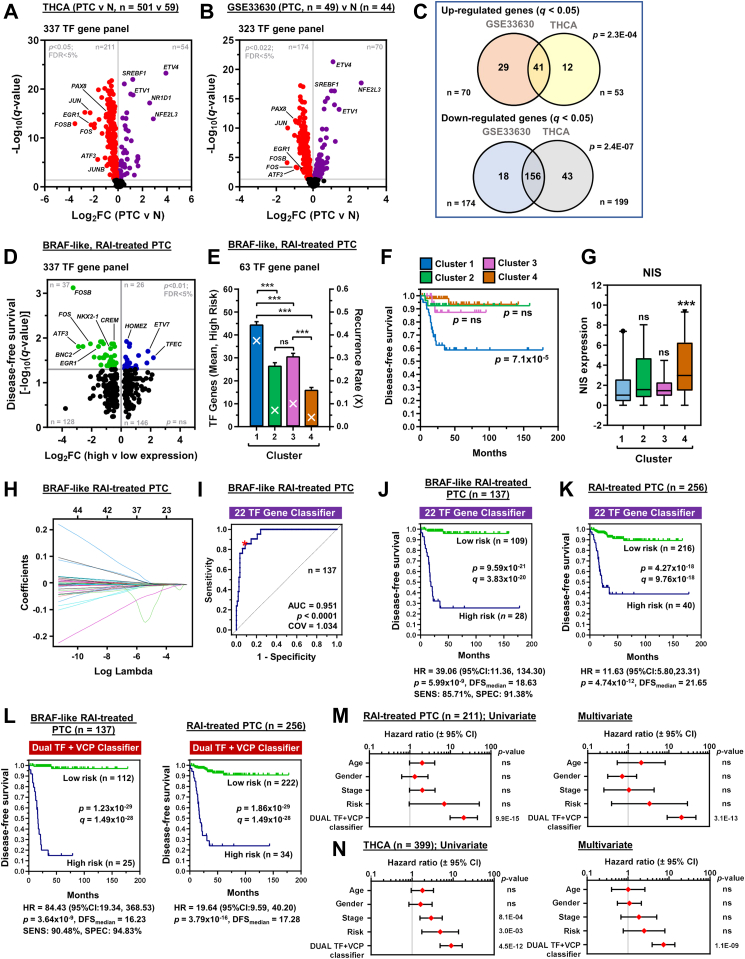
**Transcription factor and VCP/proteostasis genes predict recurrence risk in RAI-treated PTC**. (A) Volcano plot comparing log_2_FC with *q*-value (-log base 10) for the THCA PTC cohort and 337 transcription factor (TF) genes. **(B)** Volcano plot comparing log_2_FC with *q*-value (-log base 10) for the GSE33630 dataset and 323 TF genes. **(C)** Venn diagram illustrating TF genes common to both GSE33630 and THCA PTC datasets. **(D)** Volcano plot illustrating log_2_FC compared to *q*-value (-log base 10) for disease-free survival (DFS) in the BRAF-like, RAI-treated PTC cohort and 337 TF gene panel. **(E)** Mean number of dysregulated TF genes stratified into high-risk group (bars; left y-axis) and recurrence rate (white crosses; right y-axis) in patient clusters 1 to 4 (n = 20–49). **(F)** Representative Kaplan–Meier analysis of DFS for BRAF-like, RAI treated PTC stratified into patient clusters 1 to 4, log-rank test. **(G)** Box and whisker plot showing NIS expression (log_2_) in BRAF-like, RAI-treated PTC stratified into patient clusters 1 to 4, Kruskal–Wallis test followed by Dunn's post hoc test (ns, not significant, ^∗∗∗^*P* < 0.001). **(H)** LASSO regression analysis used to construct a 22 TF gene riskscore classifier. LASSO coefficient plot (loglambda). Y-axis: coefficient value; x-axis (lower): log(λ) value, and x-axis (upper): gene number. **(I**–**K)** ROC analysis (I) and Kaplan–Meier curve of the 22 TF gene riskscore classifier in BRAF-like, RAI-treated PTC (J) or RAI-treated PTC (K). **(L)** Kaplan–Meier analysis of DFS in BRAF-like, RAI-treated PTC (*left*) or RAI-treated PTC (*right*) stratified with the dual TF + VCP riskscore classifier. **(M)** Uni- (*left*) and multivariate analysis (*right*) of RAI-treated PTC (n = 211) stratified with the dual TF + VCP classifier, adjusting for the covariates age, sex, disease stage and ATA risk group in the multivariate model. **(N)** Same as (M) but with the entire TCGA THCA cohort (n = 399).

Having identified that Cu(DDC)_2_ targets multiple pathways to enhance radionuclide uptake, we next investigated the clinical relevance of Cu(DDC)_2_-associated transcription factors (TF) linked to PTC recurrence risk post-RAI therapy (Supplementary Figure S10). Importantly, we identified significant dysregulation of Cu(DDC)_2_-perturbed TF common to both TCGA THCA and GSE33630 PTC datasets (Fig. 5A–C). Hierarchical cluster analysis identified distinct clusters associated with either a predominately BRAF- (clusters 2 and 3) or RAS-like (clusters 1 and 4) gene signature (Supplementary Figure S11),^55^ mirroring significant alterations in TF expression in the differentiation states of BRAF- and RAS-like PTC (Supplementary Figure S12A–E). Moreover, Cu(DDC)_2_-perturbed TF were identified in recurrent PTC (Supplementary Figure S12F; v non-recurrent, *n* = 455), as well as in the more aggressive recurrent BRAF-like and RAI-treated PTC cohorts (Supplementary Figure S12G).

TF have pivotal roles in thyrocyte development, dedifferentiation, RAI avidity and tumour aggressiveness.^51^^,^^56^ Here, the expression of 151 Cu(DDC)_2_-perturbed TF, including PAX8 and AP-1 TF family members, were highly correlated with thyroid differentiation status in BRAF-like PTC (Supplementary Figure S13A and B). Importantly, a putative redifferentiation effect of Cu(DDC)_2_ contributing to restoration of RAI uptake was supported by a significant inverse relationship of our TF expression data (Fig. 2C) with TCGA THCA (Supplementary Figure S13C), and the independent GEO PTC dataset GSE33630 (Supplementary Figure S13D).

Critically, ROC determination in BRAF-like, RAI-treated PTC identified 63 clinically relevant TF genes linked with reduced disease-free survival (Fig. 5D; Supplementary Figure S14A) and increased risk of recurrence (Supplementary Figure S14B and C). Subsequent hierarchical cluster analysis revealed a strong association between dysregulated TF gene expression and recurrence (cluster 1, Fig. 5E and F, Supplementary Figure S15A), which was not linked with ATA risk and disease staging classification (Supplementary Figure S15B and C). Expression of NIS (Fig. 5G) and known transcriptional regulators were however lower for patients with greater recurrence (Supplementary Figure S15D, cluster 1 versus 4), despite an equivalent thyroid differentiation score (Supplementary Figure S15E).

Using a machine-learning algorithm, we next constructed a panel of multigene riskscore classifiers for predicting recurrence (13, 16 and 22 genes; Fig. 5H, Supplementary Figure S16A). Importantly, the 22 TF gene-based riskscore had the highest predictive effect as indicated by an AUC value of 0.951, along with substantially reduced DFS (Fig. 5I and J), compared to individual TF genes (Supplementary Figure S14B, S14C, S16B and S16C, AUC 0.544–0.727) and other multigene classifiers (Supplementary Figure S16D and E, Supplementary Table S3). Patients at higher risk also had a significantly worse prognosis (Fig. 5J, HR = 39.06, 95% CI 11.36–134.30), which was validated in larger THCA cohorts (Fig. 5K, Supplementary Figure S16F).

Previously we reported that a 13 VCP/proteostasis gene-based riskscore was predictive of PTC recurrence.^17^ Extrapolating from our mechanistic insights, we used a machine learning algorithm to combine both TF and VCP/proteostasis genes and generate a riskscore classifier with even greater sensitivity (90.48%), specificity (94.85%), PPV (76%) and NPV (98.2%) for stratifying recurrence risk in RAI-treated PTC compared to a panel of gene classifiers (Fig. 5L, Supplementary Figure S17A–D and S18A–D, Supplementary Table S4). Importantly, multivariate analysis reinforced the superior performance of the combined TF and VCP riskscore as an independent predictor of patient outcome after RAI therapy with adjustment for covariates, including age, sex, disease stage and ATA risk group (Fig. 5M and N, Supplementary Tables S5 and S6).

### Cu(DDC)_2_ and SAHA augment NIS function in breast cancer *in vivo*

**Fig. 6:**
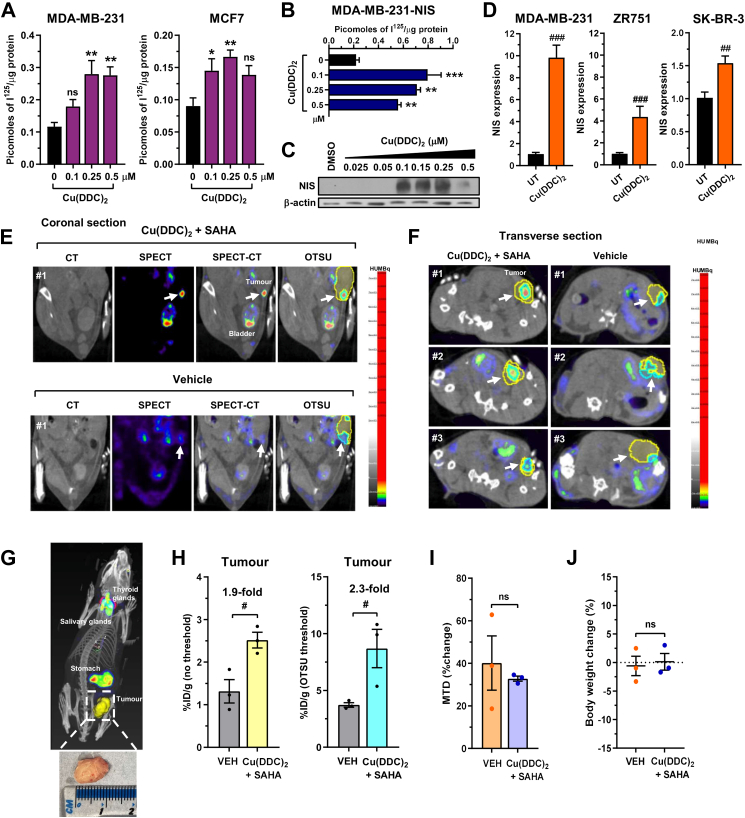
**Cu(DDC)_2_ and SAHA stimulate endogenous NIS activity in breast cancer**. (A) RAI uptake of Cu(DDC)_2_-treated parental breast cancer MDA-MB-231 and MCF7 cells. **(B)** Same as (A) but in NIS stable expressing MDA-MB-231-NIS cells. **(C)** Western blot analysis of NIS expression in Cu(DDC)_2_-treated MDA-MB-231-NIS cells. Mwt: NIS (75–100 kDa), β-actin (44 kDa). **(D)** RAI uptake of Cu(DDC)_2_-treated MDA-MB-231, ZR751 and SKBR3 breast cancer cells. **(E)** SPECT/CT images of technetium-99m (^99m^TcO4^-^) uptake in parental MDA-MB-231 mammary pad xenografts. Mice were treated with either Cu(DDC)_2_ + SAHA (*upper*) or vehicle (*lower*). Images: CT, SPECT, SPECT-CT (with relative location of tumour and bladder indicated) and SPECT-CT with OTSU thresholding analysis of tumour region. **(F)** Same as (E) but showing transverse section for SPECT-CT image with OTSU thresholding analysis of tumour region. **(G)** Representative SPECT/CT image showing relative location of tumour region defined for data analysis. Relative location of stomach, salivary glands and thyroid glands are indicated. (*Below*) Representative image of MDA-MB-231 orthotopic tumour. **(H)**^99m^TcO4^-^ uptake in parental MDA-MB-231 mammary pad xenograft tumours quantified from SPECT/CT images (n = 3/group). Data shown as %injected dose per gram (%ID/g) with either no thresholding (*left*) or with OTSU thresholding analysis (*right*). Total animals used = 6. **(I** and **J)** Mean tumour diameter (MTD) (I) and body weight change (%) (J) in female NOD.Cg-Prkdc^scid^ Il2rg^tm1Wjl^/SzJ (NSG) mice bearing MDA-MB-231 orthotopic tumours. Data presented as mean ± SEM (n = 3), (A and B) one-way ANOVA, Dunnett's post hoc test (ns, not significant, ^∗^*P* < 0.05, ^∗∗^*P* < 0.01, ^∗∗∗^*P* < 0.001), (D, H, I and J) unpaired two-tailed t-test (^#^*P* < 0.05, ^##^*P* < 0.01, ^###^*P* < 0.001).

Finally, we sought to investigate the potentially broader clinical application for Cu(DDC)_2_ to enhance radionuclide uptake in breast cancer cells. NIS expression is up-regulated in 70–80% of breast tumours, but tumoural ^131^I uptake is typically inadequate to achieve a therapeutic effect.^11^ Here, a significant finding was that Cu(DDC)_2_ induced RAI uptake in parental (Fig. 6A, Supplementary Figure S19A), and stable NIS expressing breast cells (Fig. 6B), which was accompanied by increased NIS protein and mRNA (Fig. 6C and D). Our previous study showed that a combinatorial strategy based on the HDACi SAHA with a VCPi has a maximal impact on NIS activity *in vitro*.^17^ Given the additive effect of Cu(DDC)_2_ and SAHA to induce NIS protein in thyroid cells (Supplementary Figure S19B), we thus investigated their combined impact *in vivo* using an orthotopic MDA-MB-231 breast cancer mouse model (Supplementary Figure S19C). Importantly, SPECT/CT imaging and subsequent image quantification of tumour xenografts revealed that Cu(DDC)_2_ and SAHA increased ^99m^TcO4^-^ uptake by 2.3 ± 0.5-fold compared to vehicle controls (Fig. 6E–H, Supplementary Figure S19D, n = 3/group). No differences in tumour volume or body weight were apparent (Fig. 6I and J). These findings therefore demonstrate the potential usefulness of Cu(DDC)_2_ in modulating endogenous NIS function in a non-thyroid setting.

In summary, our study identifies a dual agonist capable of targeting NIS activity to enhance RAI uptake *in vivo* via transcriptional and VCP/proteostasis pathways, both in thyroid and breast cancer settings. Combined mechanistic and bioinformatics approaches provide insight into stratifying radioiodine-treated patients with thyroid cancer for predicting subsequent tumour recurrence.

## Discussion

Despite the effectiveness of radioiodine therapy, it is predicted that the total mortality for thyroid cancer will rise by 91.2% by 2050.^57^ The exploitation of NIS activity offers a versatile and promising opportunity to develop more effective therapeutic cancer strategies, thereby overcoming treatment failure attributed to poor tumoural ^131^I uptake. Here, advancing our previous work highlighting the impact of VCP and proteostasis inhibitors on radioiodide uptake *in vitro*,^17^^,^^20^ we enacted multiple strategies to develop drugs with translatable potential to enhance NIS function. Our findings now identify the ability of the disulfiram metabolite Cu(DDC)_2_ to significantly stimulate radionuclide uptake *in vivo*, as well as uncovering a unique dual agonist effect on NIS function.

A major hurdle limiting the development of drugs targeting NIS function is a lack of mechanistic insight governing their action. This is particularly challenging in cancer due to the inherent complexity of how NIS activity is dysregulated, including aberrant expression, trafficking and processing.^51^ The majority of preclinical and clinical strategies have focussed on re-establishing the expression of NIS.^23^^,^^58^^,^^59^ Exploring a different approach, we previously attempted to modulate the intracellular processing of NIS and identified the proteostatic inhibitor disulfiram as a potent enhancer of radioiodide uptake.^17^ This led us in the current study to define the action of a copper metabolite of disulfiram which—rather than acting solely via disulfiram's proteostatic mechanism(s)—was a significant transcriptional regulator of NIS expression. The ERRγ inverse agonist GSK5182 has similarly been identified as having a dual action on NIS expression, targeting the MAPK pathway and nuclear receptor modulation.^60^ However, to the best of our knowledge, Cu(DDC)_2_ is a previously unreported example of a dual agonist targeting proteostatic and transcriptional pathways to enhance NIS function.

Several of disulfiram's anti-tumour properties are mediated via Cu(DDC)_2_ inhibition of the VCP cofactor NPL4.^21^ VCP is a highly conserved multifunctional protein, with essential roles in pathways related to the ubiquitin-proteasome system, including ERAD, and is also central to non-proteolytic functions of ubiquitin signalling.^61^ Cu(DDC)_2_ has been shown to segregate VCP from chromatin and into inactive agglomerates by disrupting NPL4 zinc finger motifs.^21^ In our studies, we discovered a transcriptional impact of Cu(DDC)_2_ on NIS expression mediated via the key thyroid transcription factor PAX8. Transient knockdown of PAX8 prevented NIS upregulation by Cu(DDC)_2_, as well as the subsequent induction of radioiodide uptake. However, whilst NPL4 was not obligate to this, the presence of VCP remained critical. VCP has numerous cofactors in addition to NPL4, with up to 170 different post-translational modifications modulating their specific interactions.^62^ Within this complexity we suggest that as well as transcriptional activation, Cu(DDC)_2_ impacts a facet of VCP biology which is independent of the cofactor NPL4.

As Cu(DDC)_2_ is poorly soluble,^54^ we used albumin nanoencapsulation to improve its *in vivo* stability and bioavailability.^21^ Importantly, Cu(DDC)_2_-albumin retained *in vitro* impact upon NIS function, which emboldened us to progress to murine studies. Whether delivered via IV or IP routes, nanoencapsulated Cu(DDC)_2_ stimulated thyroidal ^99m^TcO4^-^ uptake in wild type BALB/c mice, implying a role in the normal physiological processing of NIS activity. Our study further demonstrated the translatable potential of Cu(DDC)_2_ by enhancement of tumoural radionuclide uptake in a murine model of thyroid cancer. It was not possible to ascertain the relative contribution of Cu(DDC)_2_'s impact on inhibiting NIS protein degradation versus enhancing NIS transcription in our murine studies. From our *in vitro* data both are important, but in determining how much either process drives enhanced radionuclide accumulation remains technically challenging. Further *in vivo* studies are now warranted, including the evaluation of different Cu(DDC)_2_ dosing regimens which may result in greater magnitudes of effect, as well as investigating the impact of our drug strategies on tumoural ablation.

NIS expression is up-regulated in many breast tumours, including triple-negative breast cancers and brain metastases.^63^^,^^64^ This raises the exciting prospect of exploiting radioiodide uptake as a therapeutic strategy, if cellular uptake was sufficient to achieve a therapeutic effect.^11^ To address this, we identified a combinatorial drug strategy based on Cu(DDC)_2_ and SAHA capable of enhancing radionuclide uptake in murine orthotopic breast tumours. Whilst our preclinical findings offer a promising approach, clinical trials will be necessary to determine its efficacy and safety in enhancing radioiodide uptake in patients with breast cancer. The FDA-approved drug SAHA has been tested in multiple breast cancer trials, but not in combination with a VCP inhibitor or radioiodide. Interestingly, SAHA combined with the proteasomal inhibitor bortezomib has been trialled in patients with breast cancer, but limited patient numbers constrained assessment of clinical efficacy.^65^

In the current study, SAHA was given to mice at a human equivalent dose of ∼500 mg for an average adult, suggesting that the drug doses used are feasible for patient utility.^66^ The anticipated short timeframe (48 h) for SAHA and Cu(DDC)_2_ administration into patients prior to radioiodine therapy should also help minimise adverse events. The risk of the thyroid glands being targeted collaterally is an additional consideration. However, thyroidal ^131^I uptake can be blocked by thyroid hormone (T3) and methimazole (MMI) treatment,^67^ making higher doses of ^131^I bioavailable to a breast tumour. Thus, we anticipate that patients with breast cancer would additionally require T3 and MMI for 2 weeks prior to radioiodide therapy.^67^

The integration of bioinformatic methodologies is a pivotal tool to provide potential insights into oncogenic mechanisms and to determine future directions.^68^^,^^69^ Extrapolating from mechanistic studies, we uncovered clinical significance of dysregulated transcription and proteostasis pathways with radioiodide treatment outcome in recurrent PTC. In particular, we propose that oncogenic dysregulation of transcription factors known to regulate NIS expression results in repressed radioiodide uptake and hence poorer tumour cell ablation and outcome. This would appear to be an unexpectedly widespread phenomenon, as 63 transcription factors showed direct correlation with outcome, including ZNF703, GABPA and ETV7, which have well-described roles in thyroid cancer.^70^, ^71^, ^72^, ^73^ Adopting a combinatorial bioinformatic approach, we further constructed a 22 gene dual riskscore, incorporating VCP/proteostasis genes, enabling still higher specificity and sensitivity, as well as being an independent predictor of recurrence.^17^ We envisage that the riskscore could be incorporated into the dynamic risk stratification that occurs for patients who have undergone a total thyroidectomy and radioiodide treatment, to tailor subsequent treatment to disease risk and recurrence surveillance.

Overall, our study elucidates a dual mechanism targeting NIS activity *in vivo* via transcriptional and VCP/proteostasis pathways to enhance radioiodide uptake, both in the canonical setting of the thyroid, but also in breast tumours, where radioiodine therapy has long been considered as a potential therapeutic option. Future studies should now appraise the impact of the drug strategies we have identified in further preclinical testing and clinical trials.

## Contributors

KB, GB, LRC, MLR and CJM conceived and designed the study; KB, JSF, CEMT, LZ, JK, BS, SW, HA, AJ, TTP and MLR performed experiments; KB, JSF, CEMT, JK, KS, MLR and CJM interpreted the data; KB, JSF, JK, BS, HA, AJ, TTP, GB, LRC, VK, WW, CMG, CS, MDR, KS, PJB, KB, MLR and CJM provided methods and resources; KB, HRN, VES, MLR and CJM wrote and edited the manuscript; DGS, SJ, MDR, MJC and CJM obtained funding for the study; MJC, VES and CJM provided supervision. All authors read and approved the final manuscript. Both MLR and CJM have verified the underlying data of this manuscript and are responsible for the decision to submit the manuscript.

## Data sharing statement

The study data is available from the corresponding author Christopher J. McCabe upon request. Email address: mccabcjz@bham.ac.uk. RNA-seq data are available from NCBI GEO (Accession number: GSE317461).

## Declaration of interests

CJM receives support from the U.S. Department of Defense and Medical Research Council to cover conference fees and travel, as well as serving as Chief Operating Officer and Secretary of the American Thyroid Association. All other authors make no further declarations of conflicting interests.

## Appendix A Supplementary data

Supplementary Figures and Tables

Supplemental Western Blots
