# Supplementary material for: Disulfiram metabolite Cu(DDC)2 enhances radionuclide uptake in vivo revealing insights into tumoural ablation resistance
Source: eBioMedicine. 2026 Feb 11;125:106165. doi: 10.1016/j.ebiom.2026.106165 (PMC12917385; doi:10.1016/j.ebiom.2026.106165)
Supplement: Supplementary Figures and Tables [file mmc1.pdf]

SUPPLEMENTARY FIGURE S1

A 8505C

northgene™
A BIOFORTUNA brand

Laboratory Report

Test Requested	Cell Line Authentication
Case Number	C-30880-1
Date Sample Received	14/02/2024
Date Sample Tested	14/02/2024
Date Sample Reported	19/02/2024

Sample Name / Cell Line	Cell Line Source / Profile Source	Sample Number	DNA Number
8505C	University of Birmingham	S-1070452	D-1070452
8505C (CVCL_1054)	Cellosaurus Database	N/A	N/A

Table of Allelic Data

	Genotypes		
STR Locus	8505C (Test Sample)	8505C (CVCL_1054) (Comparison Sample)	Match vs. Mis-Match
D5S818	10 | 11	10 | 11	Match
D13S317	13 | 13	13 | 13	Match
D7S820	10 | 10	10 | 10	Match
D16S539	12 | 12	12 | 12	Match
vWA	17 | 19	17 | 19	Match
TH01	6 | 9	6 | 9	Match
TPOX	10 | 11	10 | 11	Match
CSF1PO	12 | 13	12 | 13	Match
AMEL	X | X	X | X	Match
D3S1358	16 | 17	16 | 17	Match
D21S11	28 | 32.2	28 | 32.2	Match
D18S51	16 | 16	16 | 16	Match
Penta E	12 | 15	12 | 15	Match
Penta D	9 | 10	9 | 10	Match
D8S1179	10 | 13	10 | 13	Match
FGA	23 | 23	23 | 23	Match
D19S433	13 | 14	13 | 14	Match
D2S1338	17 | 24	17 | 24	Match

Matching Percentage: **100%**
Outcome: **Related**

B TPC-1

northgene™
A BIOFORTUNA brand

Laboratory Report

Test Requested	Cell Line Authentication
Case Number	C-30880-2
Date Sample Received	14/02/2024
Date Sample Tested	14/02/2024
Date Sample Reported	19/02/2024

Sample Name / Cell Line	Cell Line Source / Profile Source	Sample Number	DNA Number
TPC1	University of Birmingham	S-1070453	D-1070453
TPC-1 (CVCL_6298)	Cellosaurus Database	N/A	N/A

Table of Allelic Data

	Genotypes		
STR Locus	TPC1 (Test Sample)	TPC-1 (CVCL_6298) (Comparison Sample)	Match vs. Mis-Match
D5S818	8 | 10	8 | 10	Match
D13S317	11 | 12	11 | 12	Match
D7S820	11 | 11	11 | 11	Match
D16S539	9 | 9	9 | 9	Match
vWA	14 | 18	14 | 18	Match
TH01	9 | 9	9 | 9	Match
TPOX	11 | 11	11 | 11	Match
CSF1PO	11 | 12	11 | 12	Match
AMEL	X | X	X | X	Match
D3S1358	16 | 17	16 | 17	Match
D21S11	30 | 31.2	30 | 31.2	Match
D18S51	13 | 16	13 | 16	Match
Penta E	18 | 18	18 | 18	Match
Penta D	9 | 13	9 | 13	Match
D8S1179	11 | 17	11 | 17	Match
FGA	20 | 21	20 | 21	Match
D19S433	13 | 13	ǂ | ǂ	Mis-Match
D2S1338	16 | 23	ǂ | ǂ	Mis-Match

Matching Percentage: **100%**
Outcome: **Related**

C SW1736

northgene™
A BIOFORTUNA brand

Laboratory Report

Test Requested	Cell Line Authentication
Case Number	C-30880-4
Date Sample Received	14/02/2024
Date Sample Tested	14/02/2024
Date Sample Reported	19/02/2024

Sample Name / Cell Line	Cell Line Source / Profile Source	Sample Number	DNA Number
SW1736	University of Birmingham	S-1070455	D-1070455
SW1736 (CVCL_3883)	Cellosaurus Database	N/A	N/A

Table of Allelic Data

	Genotypes		
STR Locus	SW1736 (Test Sample)	SW1736 (CVCL_3883) (Comparison Sample)	Match vs. Mis-Match
D5S818	12 | 13	12 | 13	Match
D13S317	11 | 12	11 | 12	Match
D7S820	8 | 11	8 | 11	Match
D16S539	11 | 12	11 | 12	Match
vWA	16 | 19	16 | 19	Match
TH01	6 | 6	6 | 6	Match
TPOX	11 | 11	11 | 11	Match
CSF1PO	12 | 12	12 | 12	Match
AMEL	X | X	X | X	Match
D3S1358	16 | 17	16 | 17	Match
D21S11	29 | 31	29 | 31	Match
D18S51	14 | 14	14 | 14	Match
Penta E	11 | 17	11 | 17	Match
Penta D	12 | 12	12 | 12	Match
D8S1179	13 | 14	13 | 13	Mis-Match
FGA	22 | 22	22 | 22	Match
D19S433	14 | 14	ǂ | ǂ	Mis-Match
D2S1338	19 | 25	ǂ | ǂ	Mis-Match

Matching Percentage: **98%**
Outcome: **Related**

D BCPAP

northgene™
A BIOFORTUNA brand

Laboratory Report

Test Requested	Cell Line Authentication
Case Number	C-30880-5
Date Sample Received	14/02/2024
Date Sample Tested	14/02/2024
Date Sample Reported	19/02/2024

Sample Name / Cell Line	Cell Line Source / Profile Source	Sample Number	DNA Number
BCPAP	University of Birmingham	S-1070456	D-1070456
B-CPAP (CVCL_0153)	Cellosaurus Database	N/A	N/A

Table of Allelic Data

	Genotypes		
STR Locus	BCPAP (Test Sample)	B-CPAP (CVCL_0153) (Comparison Sample)	Match vs. Mis-Match
D5S818	10 | 11	10 | 11	Match
D13S317	12 | 12	12 | 12	Match
D7S820	10 | 10	10 | 10	Match
D16S539	11 | 12	11 | 12	Match
vWA	14 | 17	14 | 17	Match
TH01	6 | 9.3	6 | 9.3	Match
TPOX	8 | 11	8 | 11	Match
CSF1PO	13 | 13	13 | 13	Match
AMEL	X | X	X | X	Match
D3S1358	16 | 17	16 | 17	Match
D21S11	30 | 31.2	30 | 30	Mis-Match
D18S51	13 | 17	13 | 17	Match
Penta E	5 | 12	5 | 12	Match
Penta D	10 | 11	10 | 11	Match
D8S1179	12 | 13	12 | 13	Match
FGA	20 | 23	20 | 23	Match
D19S433	13.2 | 15	14 | 15	Mis-Match
D2S1338	18 | 18	18 | 18	Match

Matching Percentage: **95%**
Outcome: **Related**

E MDA-MB-231

northgene™
A BIOFORTUNA brand

Laboratory Report

Test Requested	Cell Line Authentication
Case Number	C-30880-3
Date Sample Received	14/02/2024
Date Sample Tested	14/02/2024
Date Sample Reported	19/02/2024

Sample Name / Cell Line	Cell Line Source / Profile Source	Sample Number	DNA Number
MDA-MB-231	University of Birmingham	S-1070454	D-1070454
MDA-MB-231 (CVCL_0062)	Cellosaurus Database	N/A	N/A

Table of Allelic Data

	Genotypes		
STR Locus	MDA-MB-231 (Test Sample)	MDA-MB-231 (CVCL_0062) (Comparison Sample)	Match vs. Mis-Match
D5S818	12 | 12	12 | 12	Match
D13S317	13 | 13	13 | 13	Match
D7S820	8 | 9	8 | 9	Match
D16S539	12 | 12	12 | 12	Match
vWA	15 | 18	15 | 18	Match
TH01	7 | 9.3	7 | 9.3	Match
TPOX	8 | 9	8 | 9	Match
CSF1PO	12 | 13	12 | 13	Match
AMEL	X | X	X | X	Match
D3S1358	16 | 16	16 | 16	Match
D21S11	30 | 33.2	30 | 33.2	Match
D18S51	11 | 16	11 | 16	Match
Penta E	11 | 11	11 | 11	Match
Penta D	11 | 14	11 | 14	Match
D8S1179	13 | 13	13 | 13	Match
FGA	22 | 23	22 | 23	Match
D19S433	11 | 14	11 | 14	Match
D2S1338	20 | 21	20 | 21	Match

Matching Percentage: **100%**
Outcome: **Related**

F ZR751

northgene™
A BIOFORTUNA brand

UKAS TESTING 7816

Laboratory Report

Test Requested	Cell Line Authentication
Case Number	C-25115c
Date Sample Received	08/04/2022
Date Sample Tested	11/04/2022
Date Sample Reported	19/04/2022

Sample Name	Sample/Comparison Profile Source	Sample Number	DNA Number
ZR751	University of Birmingham	S-1057715	D-1057715
ZR-75-1	DSMZ Database	N/A	N/A

Table of Allelic Data

	Genotypes		
STR Locus	ZR751 (Test Sample)	ZR-75-1 (Comparison Sample)	Match vs. Mis-Match
D5	13 | 13	13 | 13	Match
D13	9 | 9	9 | 9	Match
D7	10 | 11	10 | 11	Match
D16	11 | 11	11 | 11	Match
vWA	16 | 18	16 | 18	Match
Amel	X | X	X | X	Match
TPOX	8 | 8	8 | 8	Match
CSF1PO	10 | 11	10 | 11	Match
THO1	7 | 9.3	7 | 9.3	Match

Matching Percentage: **100%**
Outcome: **Related**

G HeLa

northgene™
A BIOFORTUNA brand

Laboratory Report

Test Requested	Cell Line Authentication
Case Number	C-25115f
Date Sample Received	08/04/2022
Date Sample Tested	11/04/2022
Date Sample Reported	19/04/2022

Sample Name	Sample/Comparison Profile Source	Sample Number	DNA Number
HeLa	University of Birmingham	S-1057718	D-1057718
HeLa	DSMZ Database	N/A	N/A

Table of Allelic Data

	Genotypes		
STR Locus	HeLa (Test Sample)	HeLa (Comparison Sample)	Match vs. Mis-Match
D5	11 | 12	11 | 12	Match
D13	12 | 13.3	12 | 13.3	Match
D7	8 | 12	8 | 12	Match
D16	9 | 10	9 | 10	Match
vWA	16 | 18	16 | 18	Match
Amel	X | X	X | X	Match
TPOX	8 | 12	8 | 12	Match
CSF1PO	9 | 10	9 | 10	Match
THO1	7 | 7	7 | 7	Match

Matching Percentage: **100%**
Outcome: **Related**

H HEK293

northgene™
A BIOFORTUNA brand

Laboratory Report

Test Requested	Cell Line Authentication
Case Number	C-25115h
Date Sample Received	08/04/2022
Date Sample Tested	11/04/2022
Date Sample Reported	19/04/2022

Sample Name	Sample/Comparison Profile Source	Sample Number	DNA Number
HEK293	University of Birmingham	S-1057720	D-1057720
HEK-293.2sus	DSMZ Database	N/A	N/A

Table of Allelic Data

	Genotypes		
STR Locus	HEK293 (Test Sample)	HEK-293.2sus (Comparison Sample)	Match vs. Mis-Match
D5	8 | 8	8 | 8	Match
D13	12 | 14	12 | 14	Match
D7	11 | 11	11 | 12	Mis-Match
D16	9 | 13	9 | 13	Match
vWA	16 | 19	16 | 19	Match
Amel	X | X	X | X	Match
TPOX	11 | 11	11 | 11	Match
CSF1PO	12 | 12	12 | 12	Match
THO1	7 | 9.3	7 | 9.3	Match

Matching Percentage: **94%**
Outcome: **Related**

I **AU565**

northgene™
A BIOFORTUNA brand

Laboratory Report

Test Requested Cell Line Authentication
Case Number C-25115g
Date Sample Received 08/04/2022
Date Sample Tested 11/04/2022
Date Sample Reported 19/04/2022

Sample Name	Sample/Comparison Profile Source	Sample Number	DNA Number
Aus65	University of Birmingham	S-1057719	D-1057719
AU565 [AU-565]	DSMZ Database	N/A	N/A

Table of Allelic Data

<table>
<tr><th></th><th colspan="2">Genotypes</th><th></th></tr>
<tr><th>STR Locus</th><th>Aus65 (Test Sample)</th><th>AU565 [AU-565] (Comparison Sample)</th><th>Match vs. Mis-Match</th></tr>
<tr><td>D5</td><td>9 | 12</td><td>9 | 12</td><td>Match</td></tr>
<tr><td>D13</td><td>11 | 11</td><td>11 | 12</td><td>Mis-Match</td></tr>
<tr><td>D7</td><td>9 | 12</td><td>9 | 12</td><td>Match</td></tr>
<tr><td>D16</td><td>9 | 9</td><td>9 | 9</td><td>Match</td></tr>
<tr><td>vWA</td><td>17 | 17</td><td>17 | 17</td><td>Match</td></tr>
<tr><td>Amel</td><td>X | X</td><td>X | X</td><td>Match</td></tr>
<tr><td>TPOX</td><td>8 | 11</td><td>8 | 11</td><td>Match</td></tr>
<tr><td>CSF1PO</td><td>12 | 12</td><td>12 | 12</td><td>Match</td></tr>
<tr><td>THO1</td><td>8 | 9</td><td>8 | 9</td><td>Match</td></tr>
</table>

Matching Percentage: **94%**
Outcome: **Related**

J **MCF7**

UKAS TESTING 7816

northgene™
A BIOFORTUNA brand

Laboratory Report

Test Requested Cell Line Authentication
Case Number C-28016a
Date Sample Received 04/04/2023
Date Sample Tested 04/04/2023
Date Sample Reported 18/04/2023

Sample Name / Cell Line	Cell Line Source / Profile Source	Sample Number	DNA Number
MCF-7	University of Birmingham	S-1064482	D-1064482
MCF-7 (ACC-115)	DSMZ Database	N/A	N/A

Table of Allelic Data

<table>
<tr><th></th><th colspan="3">Genotypes</th></tr>
<tr><th>STR Locus</th><th>MCF-7 (Test Sample)</th><th>MCF-7 (ACC-115) (Comparison Sample)</th><th>Match vs. Mis-Match</th></tr>
<tr><td>D5S818</td><td>11 | 12</td><td>11 | 12</td><td>Match</td></tr>
<tr><td>D13S317</td><td>11 | 11</td><td>11 | 11</td><td>Match</td></tr>
<tr><td>D7S820</td><td>8 | 9</td><td>8 | 9</td><td>Match</td></tr>
<tr><td>D16S539</td><td>11 | 12</td><td>11 | 12</td><td>Match</td></tr>
<tr><td>vWA</td><td>14 | 15</td><td>14 | 15</td><td>Match</td></tr>
<tr><td>TH01</td><td>6 | 6</td><td>6 | 6</td><td>Match</td></tr>
<tr><td>TPOX</td><td>9 | 12</td><td>9 | 12</td><td>Match</td></tr>
<tr><td>CSF1PO</td><td>10 | 10</td><td>10 | 10</td><td>Match</td></tr>
<tr><td>AMEL</td><td>X | X</td><td>X | X</td><td>Match</td></tr>
<tr><td>D3S1358</td><td>16 | 16</td><td>16 | 16</td><td>Match</td></tr>
<tr><td>D21S11</td><td>30 | 30</td><td>30 | 30</td><td>Match</td></tr>
<tr><td>D18S51</td><td>14 | 14</td><td>14 | 14</td><td>Match</td></tr>
<tr><td>Penta E</td><td>7 | 12</td><td>7 | 12</td><td>Match</td></tr>
<tr><td>Penta D</td><td>12 | 12</td><td>12 | 12</td><td>Match</td></tr>
<tr><td>D8S1179</td><td>10 | 14</td><td>10 | 14</td><td>Match</td></tr>
<tr><td>FGA</td><td>23 | 24 | 25</td><td>23 | 24 | 25</td><td>Match</td></tr>
<tr><td>D19S433</td><td>13 | 14</td><td>13 | 14</td><td>Match</td></tr>
<tr><td>D2S1338</td><td>21 | 23</td><td>21 | 23</td><td>Match</td></tr>
</table>

Matching Percentage: **100%**
Outcome: **Related**

K **SK-BR-3**

DSMZ Scientific Data:

Date Sample: 3/5/2022

Authenticity:
STR analysis according to the global standard ANSI/ATCC ASN-0002.1-2021 (2021) resulted in an authentic STR profile of the reference STR database.

Fig. S1: **STR profiles of cell lines used in study.** Representative examples of cell line authentication reports for **(A)** 8505C, **(B)** TPC-1, **(C)** SW1736, **(D)** BCPAP, **(E)** MDA-MB-231, **(F)** ZR751, **(G)** HeLa, **(H)** HEK293, **(I)** AU565 and **(J)** MCF7 cells (NorthGene, Biofortuna). DNA profiles were compared to profiles located on the Cellosaurus database. Analysis was conducted using 8 loci allowing identical matches and approximate power of discrimination of 1 in 1,000,000,000. Cell lines were considered to be related with a matching percentage > 80%. **(K)** STR profiling report provided by DSMZ after purchase of SK-BR-3 cells (3/5/2022). Cells used within a limit of 10 passages to avoid overpassaging.

SUPPLEMENTARY FIGURE S2

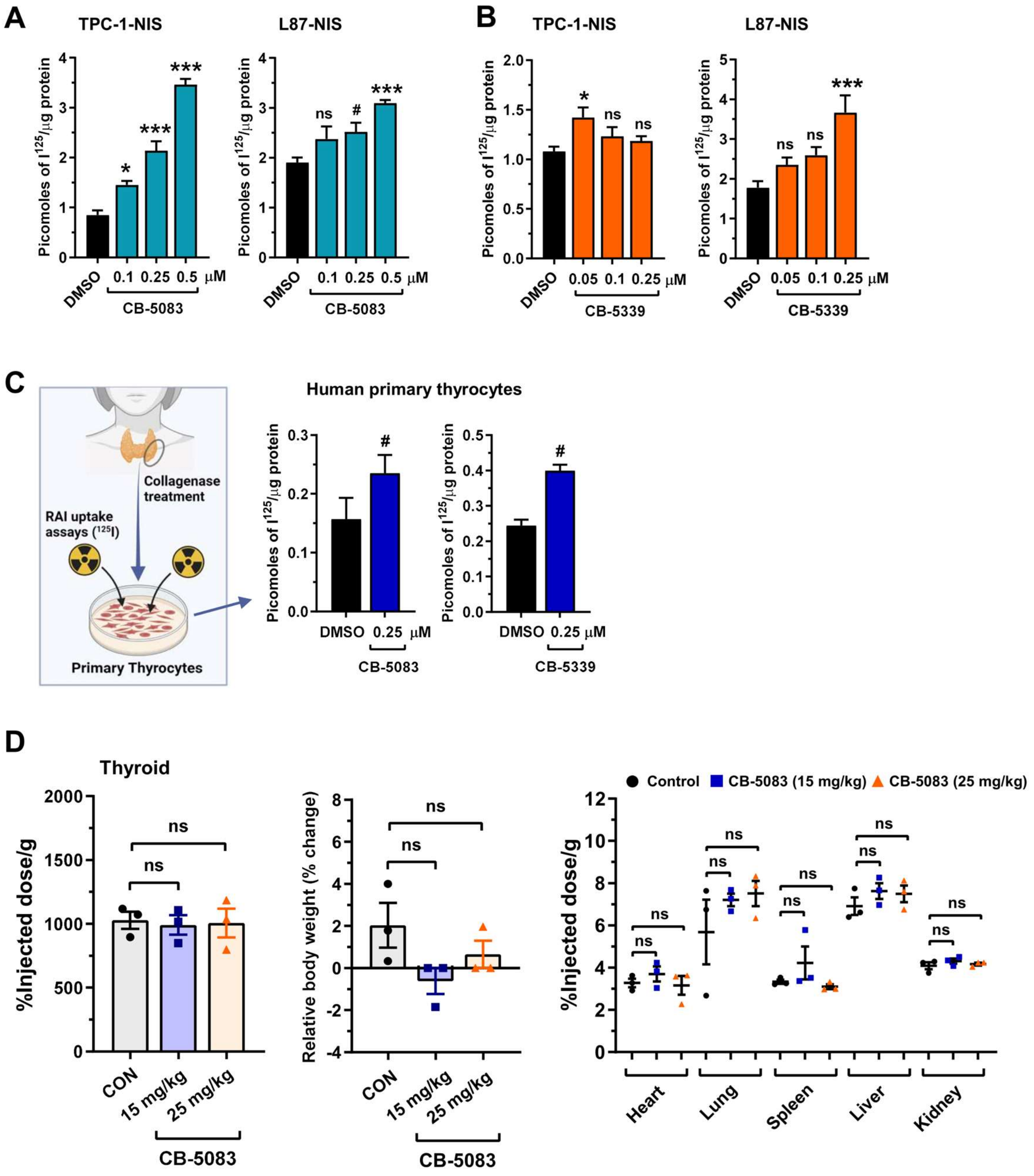

Fig. S2: **Evaluation of clinically trialled VCPi CB-5083 and CB-5339 on NIS activity. (A** and **B)** RAI uptake in TPC-1-NIS and L87-NIS cells treated with CB-5083 (A) or CB-5339 (B) for 24 hours. **(C)** RAI uptake in human primary thyrocytes treated with CB-5083 or CB-5339. *Left*: Schematic illustrating culturing of human primary thyrocytes and RAI uptake assays. Created with BioRender.com. **(D)** *Left*: Technetium-99m pertechnetate (^{99m}TcO4^{-}) uptake (n = 3) in thyroid glands dissected from wild-type (WT) BALB/c mice administered by oral gavage with CB-5083 at indicated doses. Mice were given a total of 4 doses. *Middle*: Body weight change (%) in WT BALB/c mice administered with CB-5083 at indicated doses versus controls (n = 3 per group). *Right*: Biodistribution

of $^{99m}TcO4^-$ uptake across tissues harvested from WT BALB/c mice administered with CB-5083 at indicated doses versus controls (n = 3 per group). Total animals used = 9. Data presented as mean ± S.E.M ($n = 3$); one-way ANOVA, Dunnett's post hoc test (ns, not significant; $*p < 0.05$; $***p < 0.001$); unpaired two-tailed t-test ($^{\#}p < 0.05$).

SUPPLEMENTARY FIGURE S3

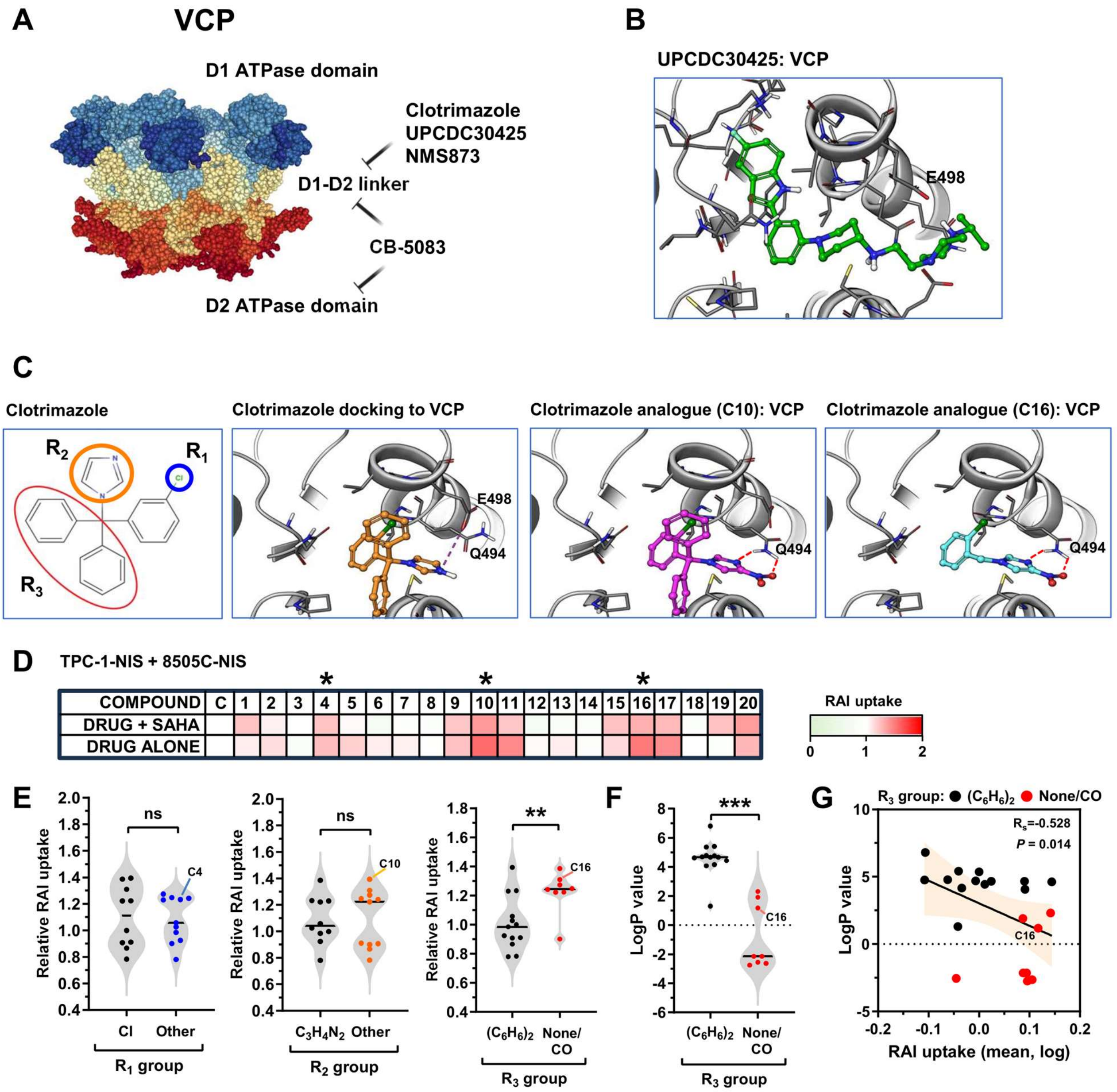

Fig. S3: **Debulking of aryl groups in clotrimazole enhances NIS activity and drug bioavailability.** **(A)** Schematic illustrating VCP structure and regions targeted by VCP inhibitors. Targeted inhibition of the D1 to D2 20 residue linker region prevents conformational changes required for VCP function, while targeting the D2 domain inhibits ATPase enzyme activity. **(B)** Computational modelling of allosteric VCP inhibitor UPCDC30425 (green) binding to VCP. **(C)** Modelling the docking of clotrimazole and analogues (C10 and C16) to VCP. Dashed lines: predicted hydrogen bonds with residues (Q494, E498) in allosteric binding pocket of VCP. Left: Chemical structure of clotrimazole highlighting modifications made at the chloro-substituted aryl ring (R_1; blue), imidazole ring (R_2;

orange) and aryl substituent groups (R_3; red). **(D)** Heat map depicting mean RAI uptake in TPC-1-NIS and 8505C-NIS cells treated with 20 analogues versus clotrimazole (C) alone (*lower*) or in combination with SAHA (*upper*) with significant differences indicated (asterisk). **(E)** Mean RAI uptake in thyroid cancer cells (8505C-NIS, TPC-1-NIS) treated with clotrimazole analogues with an intact (R_1 group-Cl) or modified chloro-substituted aryl ring (R_1 group-Other) (*left*), an intact (R_2 group-$C_3H_4N_2$) or modified imidazole ring (R_2 group-Other) (*middle*), or an intact [R_3 group-$(C_6H_6)_2$] versus modified aryl substituent groups (R_3 group-None/CO) (*right*). Unpaired two-tailed t-test (ns, not significant; $^{**}P < 0.01$). **(F)** Comparison of logP values for clotrimazole analogues with an intact (black) versus modified R_3 group (red). Unpaired two-tailed t-test ($^{***}P < 0.001$). **(G)** Correlation analysis between mean RAI uptake and logP values of clotrimazole analogues with an intact (black) or modified R_3 aryl group (red).

SUPPLEMENTARY FIGURE S4

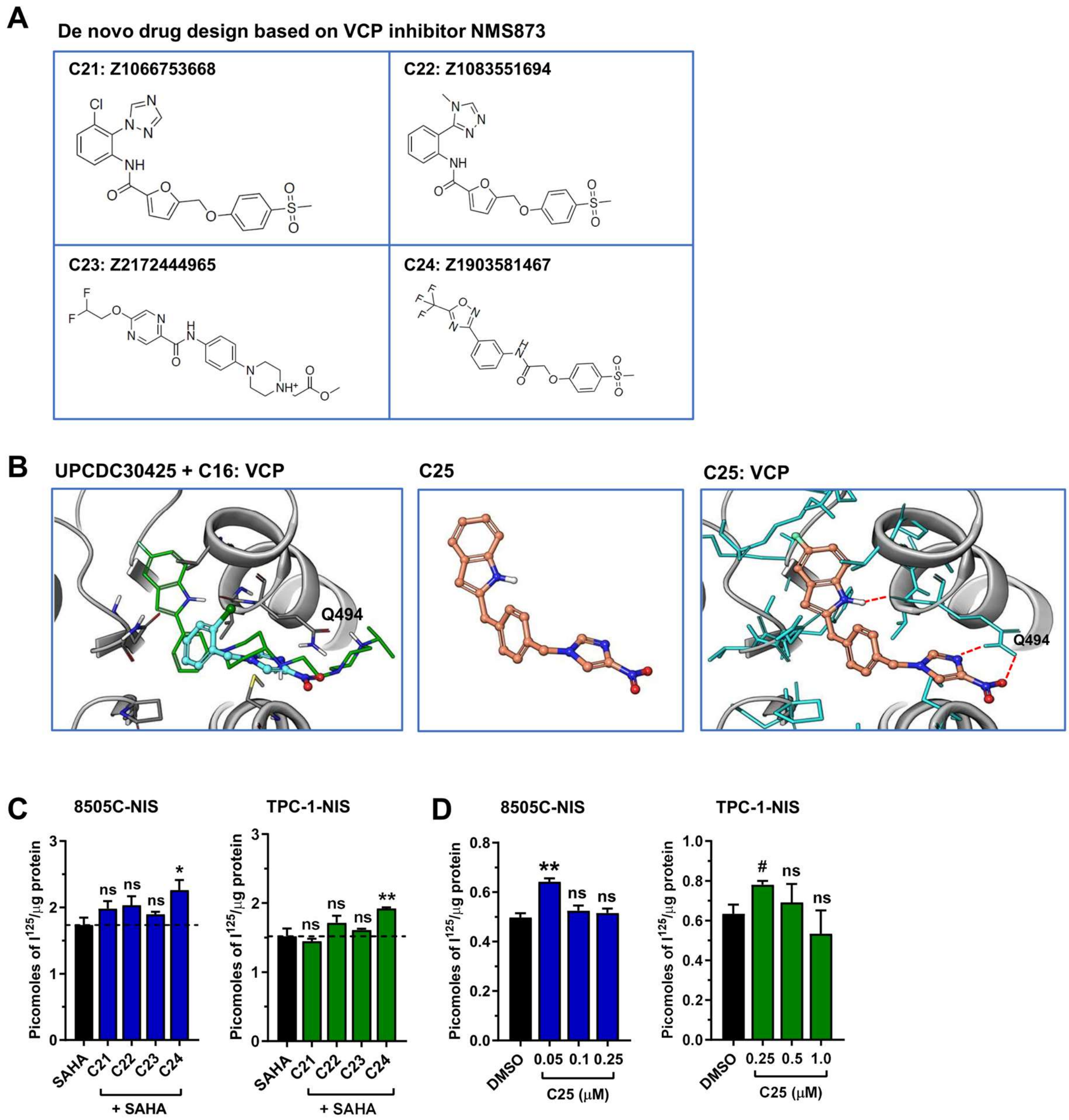

***Fig. S4:* Evaluation of VCP inhibitors based on the cryo-EM structure of NMS783. (A)** Chemical structures of compounds C21 to C24 based on the published cryo-EM structure of VCP inhibitor NMS873. **(B)** *Left*: Docking of UPCDC30425 (green) to VCP showed that the piperazine tail extended farther into the binding pocket than compound C16 (cyan). Based on this, compound C25 was generated as a modified compound C16 mimicking the piperazine tail of UPCDC30425 to capture additional VCP interactions. *Middle*: Chemical structure of compound C25. *Right*: Compound C25 modelled in the binding pocket of VCP. Dashed lines: predicted hydrogen bonds with residues (e.g. Q494) in the VCP allosteric binding pocket. **(C)** RAI uptake in 8505C-NIS and TPC-1-NIS cells treated with compounds C21 to C24 in combination with SAHA versus SAHA alone. Dashed line: RAI uptake using SAHA

alone. **(D)** RAI uptake of 8505C-NIS and TPC-1-NIS cells treated with compound C25 at indicated doses for 24 hours. Data presented as mean ± S.E.M (n = 3); one-way ANOVA followed by Dunnett's post hoc test (ns, not significant; $*P < 0.05$; $**P < 0.01$); unpaired two-tailed t-test ($^{\#}P < 0.05$).

SUPPLEMENTARY FIGURE S5

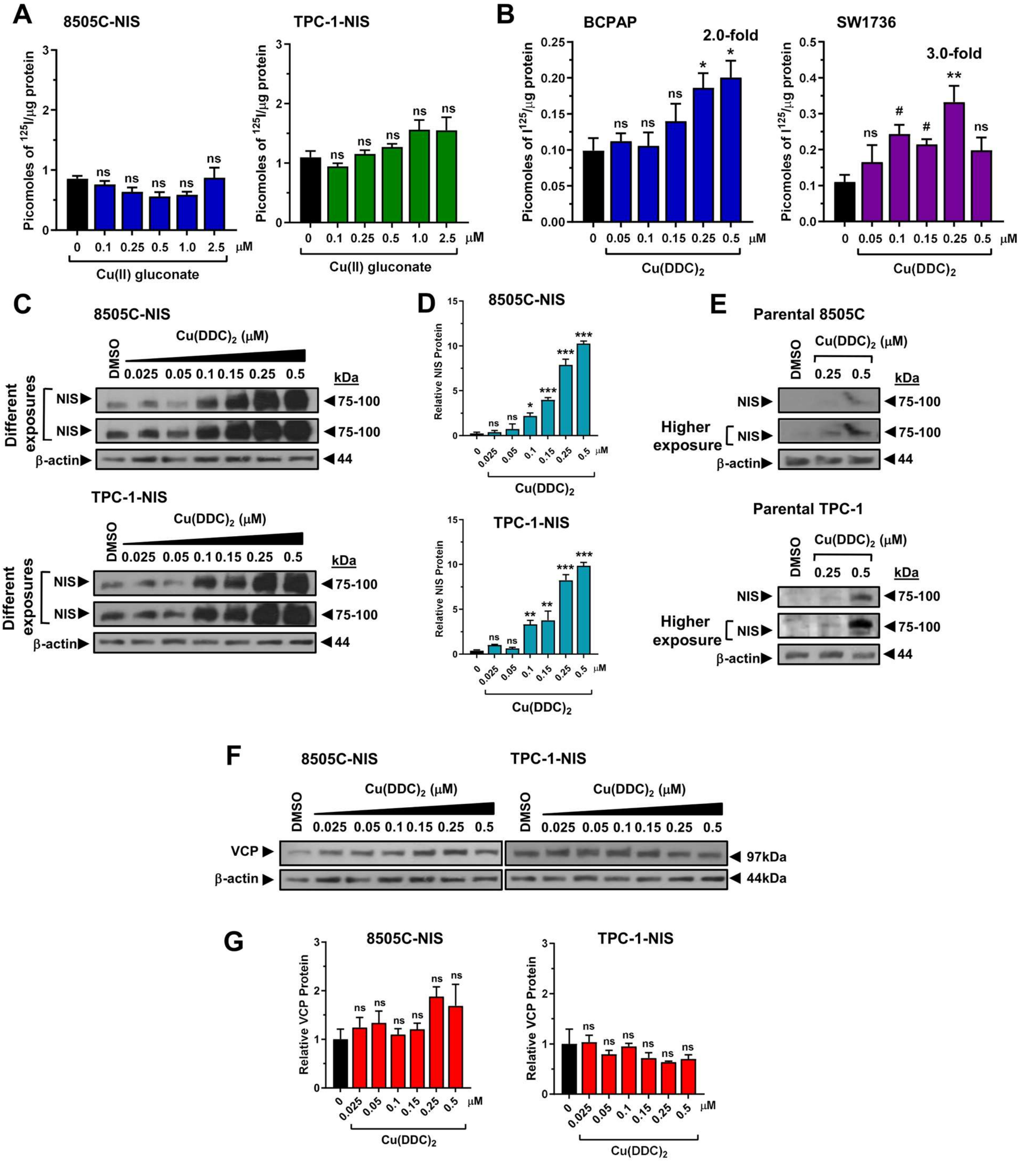

Fig. S5: **Disulfiram metabolite enhances NIS protein levels in thyroid cancer cells. (A)** RAI uptake of 8505C-NIS and TPC-1-NIS cells treated with copper gluconate [Cu(II)] at indicated doses for 24 hours. **(B)** RAI uptake of BCPAP and SW1736 cells treated with $Cu(DDC)_2$ at indicated doses for 24 hours. **(C)** Western blot analysis of NIS expression in $Cu(DDC)_2$-treated 8505C-NIS and TPC-1-NIS cells at different exposure times. **(D)** Quantification of NIS protein levels in 8505C-NIS and TPC-1-NIS cells treated with $Cu(DDC)_2$ at indicated doses for 24 hours. **(E)** Western blot analysis of NIS expression in $Cu(DDC)_2$-treated parental 8505C and TPC-1 cells at indicated doses. **(F)** Western blot

analysis of VCP expression in 8505C-NIS and TPC-1-NIS cells treated with $Cu(DDC)_2$ at indicated doses for 24 hours. **(G)** Quantification of VCP protein levels in $Cu(DDC)_2$-treated 8505C-NIS and TPC-1-NIS cells. Data presented as mean ± S.E.M (n = 3); one-way ANOVA followed by Dunnett's post hoc test (ns, not significant; $*P < 0.05$; $**P < 0.01$; $***P < 0.001$); unpaired two-tailed t-test ($^{\#}P < 0.05$).

SUPPLEMENTARY FIGURE S6

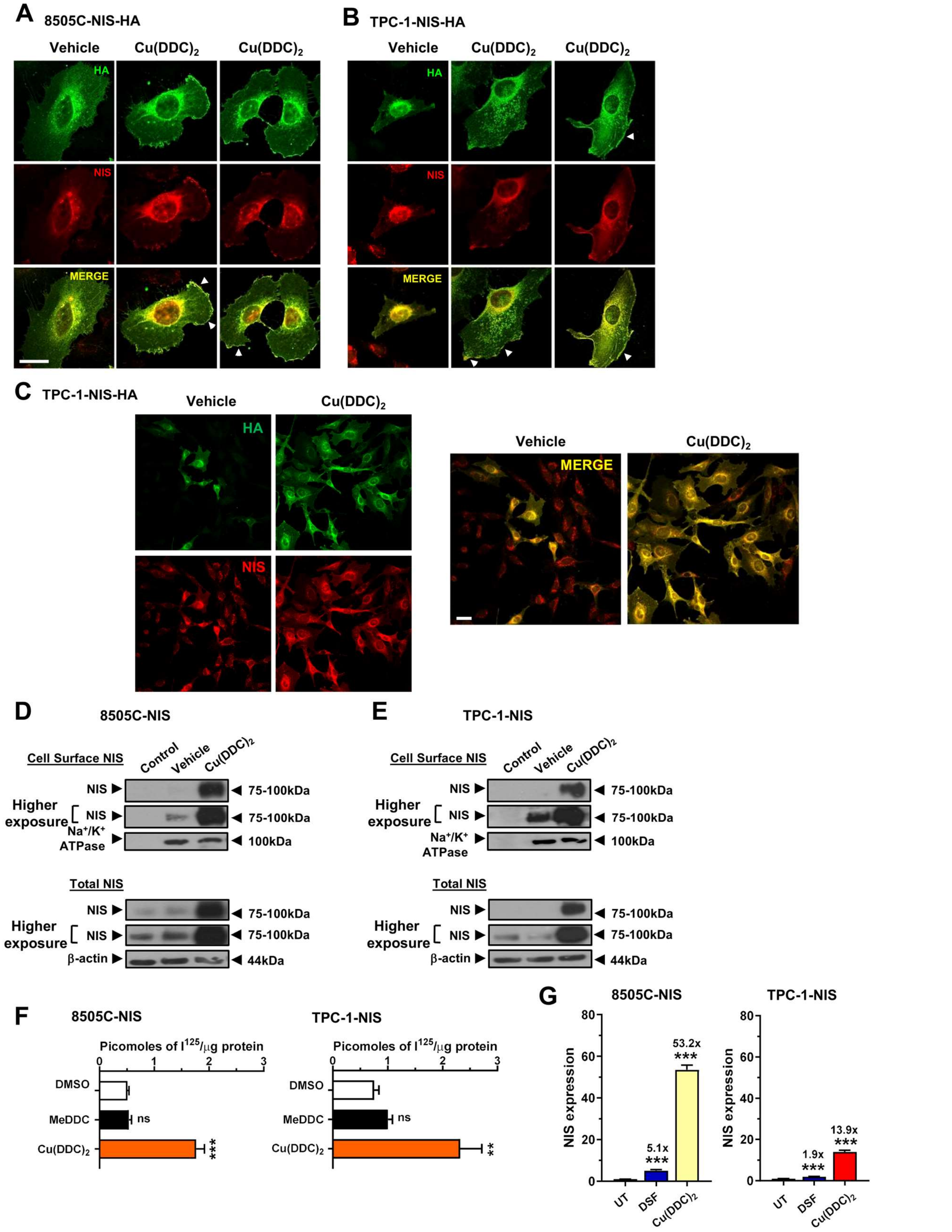

161

162

***Fig. S6:* Disulfiram metabolite enhances NIS protein at the plasma membrane. (A** and **B)** Confocal imaging in 8505C-NIS-HA (A) and TPC-1-NIS-HA (B) cells treated with 0.25 µM $Cu(DDC)_2$ for 24 hours. Confocal images represent HA expression (green), NIS expression (red) and a merged image (yellow). Arrows (white): Plasma membrane regions with enhanced NIS localisation. Scale bar: 20 µm. **(C)** Same as (B) but showing multiple $Cu(DDC)_2$-treated TPC-1-NIS-HA cells. **(D** and **E)** Western blot analysis of NIS protein levels at the plasma membrane (cell surface NIS) relative to Na+/K+ ATPase following the CSBA in $Cu(DDC)_2$-treated 8505C-NIS (D) and TPC-1-NIS (E) cells. Different exposures of NIS expression are given. Control: Biotin tag omitted (*upper*), total protein before biotin separation (*lower*). **(F)** RAI uptake assays in 8505C-NIS (*left*) and TPC-1 NIS cells (*right*) treated with $Cu(DDC)_2$ and S-methyl N,N-diethyldithiocarbamate (MeDDC). **(G)** Relative NIS mRNA levels in 8505C-NIS and TPC-1-NIS cells treated with either disulfiram (DSF) or $Cu(DDC)_2$. Data presented as mean ± S.E.M (n = 3); one-way ANOVA followed by Dunnett's post hoc test (ns, not significant; $^{**}P < 0.01$; $^{***}P < 0.001$).

.

SUPPLEMENTARY FIGURE S7

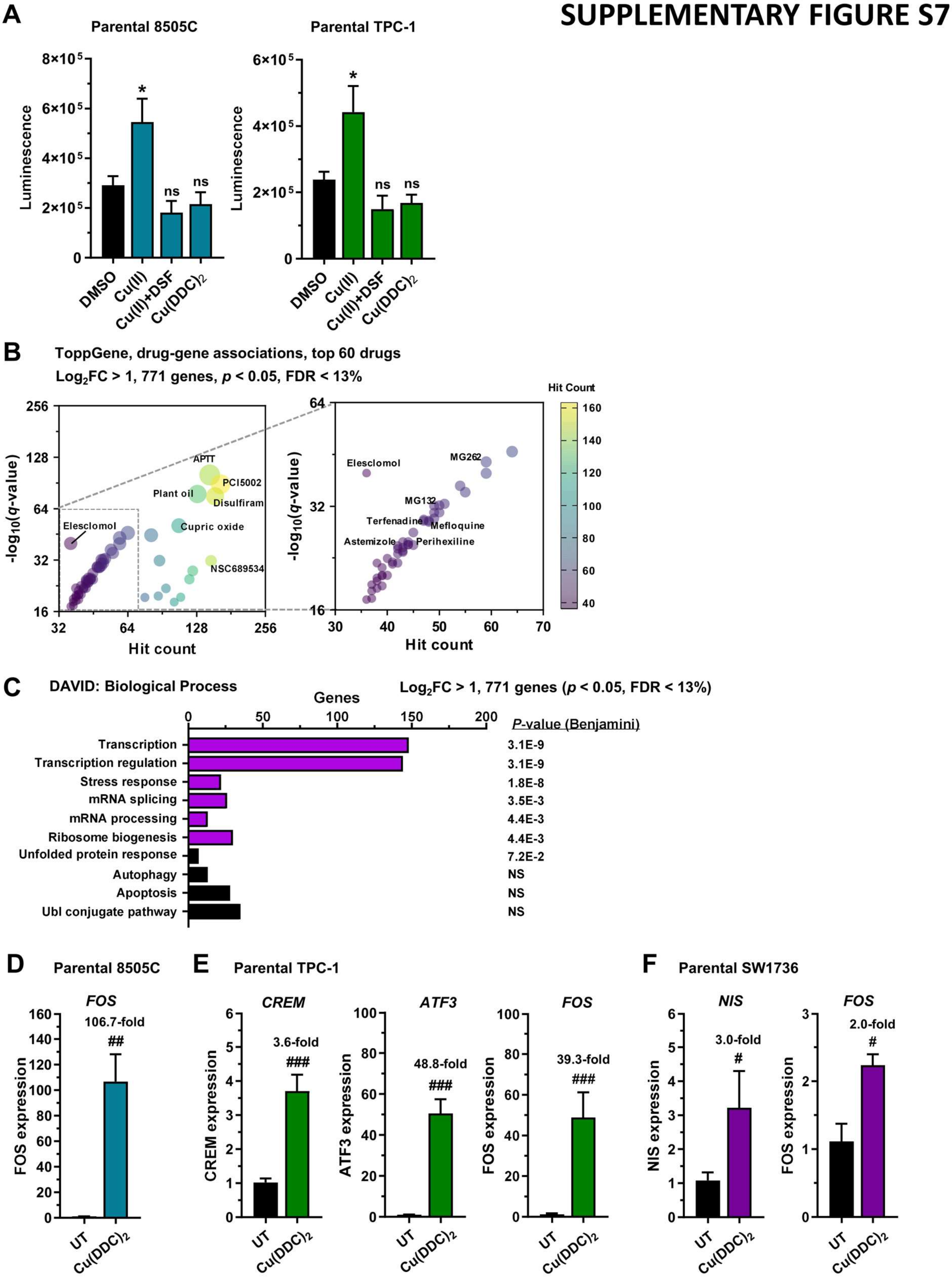

Fig. S7: **Cu(DDC)$_2$ induces transcriptional pathways in thyroid cancer cells. (A)** ROS-Glo assay evaluation of H_2O_2 levels in parental 8505C and TPC-1 cells treated with Cu(II), Cu(II)+DSF and Cu(DDC)$_2$ versus DMSO. **(B)** ToppGene classification of drug-gene associations for 771 differentially expressed genes ($log_2FC > 1$, $P < 0.05$, FDR < 13%) in parental 8505C cells treated with 0.25 μM Cu(DDC)$_2$. *Right*: enlarged view of drug-gene associations with hit count < 64. **(C)** DAVID functional classification of 771 differentially expressed genes ($log_2FC > 1$, $P < 0.05$, FDR < 13%) in parental 8505C cells treated with Cu(DDC)$_2$. **(D)** Relative FOS mRNA in parental 8505C cells treated with Cu(DDC)$_2$. **(E)** Relative CREM, ATF3 and FOS mRNA in parental TPC-1 cells treated with Cu(DDC)$_2$. Cu(DDC)$_2$ dose: 0.25 μM. **(F)** Relative NIS and FOS mRNA in parental SW1736 cells treated with Cu(DDC)$_2$. Data presented as mean ± S.E.M (n = 3); one-way ANOVA followed by Dunnett's post hoc test (ns, not significant; $^*P < 0.05$); unpaired two-tailed t-test ($^{\#}P < 0.05$; $^{\#\#}P < 0.01$; $^{\#\#\#}P < 0.001$).

SUPPLEMENTARY FIGURE S8

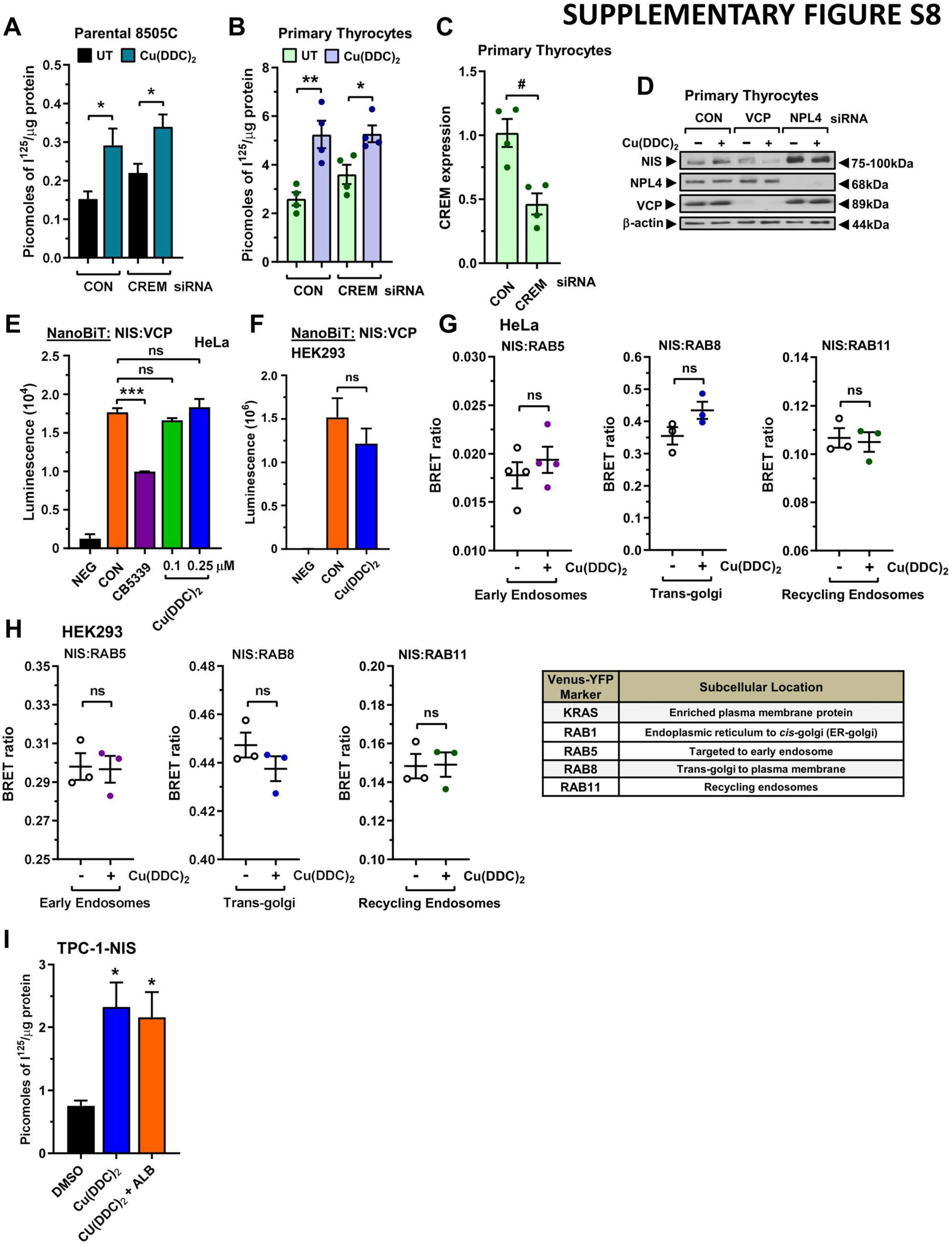

Venus-YFP Marker	Subcellular Location
KRAS	Enriched plasma membrane protein
RAB1	Endoplasmic reticulum to *cis*-golgi (ER-golgi)
RAB5	Targeted to early endosome
RAB8	Trans-golgi to plasma membrane
RAB11	Recycling endosomes

Fig. S8: **$Cu(DDC)_2$ acts as a dual agonist to enhance NIS activity. (A and B)** RAI uptake in parental 8505C cells (A) and human primary thyrocytes (B) following CREM-siRNA depletion and $Cu(DDC)_2$ treatment. CON – scrambled control siRNA. **(C)** Relative CREM mRNA in human primary thyrocytes following CREM-siRNA depletion. **(D)** Western blot analysis of NIS, VCP and NPL4 in human primary thyrocytes following VCP- or NPL4-siRNA depletion. **(E)** NanoBiT evaluation of protein: protein interaction between NIS and VCP in living HeLa cells treated with 0.25 μM CB5339 or $Cu(DDC)_2$ at indicated doses. NanoBiT assay results at 20 minutes post-addition of Nano-Glo live cell assay substrate. **(F)** Same as (E) but in live HEK293 cells treated with 0.25 μM $Cu(DDC)_2$. **(G and H)** NanoBRET evaluation of NIS localisation in early endosomes (RAB5), trans-golgi network (RAB8) or recycling endosomes (RAB11) in live HeLa (G) or HEK293 (H) cells treated with $Cu(DDC)_2$. **(I)** RAI uptake in TPC-1-NIS cells treated with $Cu(DDC)_2$ in DMSO or nanoencapsulated in albumin (ALB). Data presented as mean ± S.E.M ($n \geq 3$); one-way ANOVA followed by Dunnett's or Tukey's post hoc test (ns, not significant; *$P < 0.05$; **$P < 0.01$; ***$P < 0.001$); unpaired two-tailed t-test ($^{\#}P < 0.05$).

SUPPLEMENTARY FIGURE S9

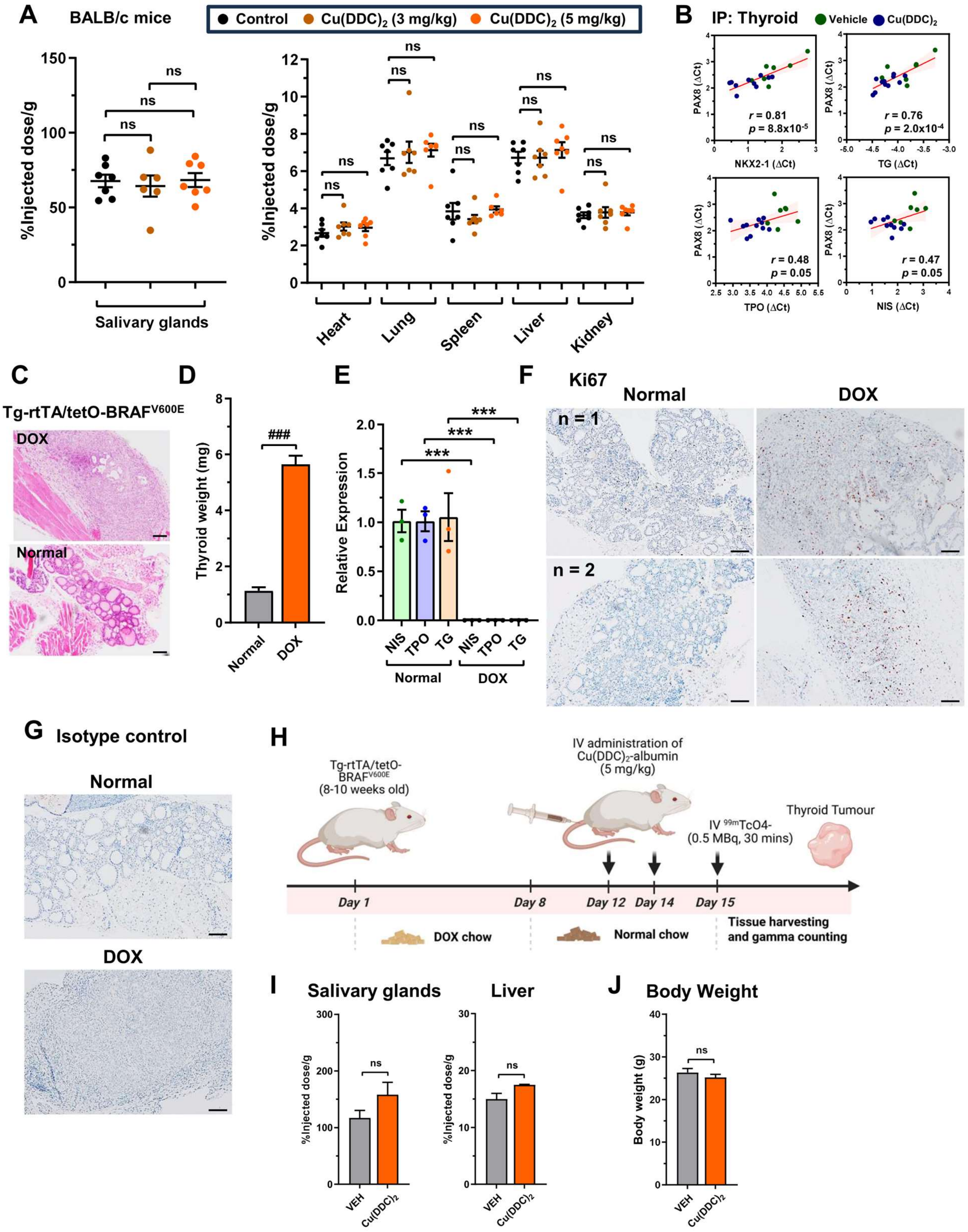

Fig. S9: **Tg-rtTA/tetO-BRAF^{V600E} mouse model to study the impact of Cu(DDC)$_2$ on radionuclide uptake in thyroid cancer. (A)** Biodistribution of $^{99m}TcO4^-$ uptake in tissues harvested from WT BALB/c mice administered with Cu(DDC)$_2$-ALB at dose of 3 mg/kg (brown dot) or 5 mg/kg (orange dot) versus controls (n = 5-7 per group). Total animals used = 21. **(B)** Correlation of PAX8 with thyroid genes *NKX2-1*, *TG*, *TPO* and *NIS* (ΔCT values, Pearson) in thyroids from Cu(DDC)$_2$-ALB treated WT BALB/c mice. 95% CI (upper/lower) are shown. **(C)** Representative H&E stained images of thyroid tissue from Tg-rtTA/tetO-BRAF^{V600E} mice fed with DOX (*upper*) versus normal (*lower*) chow for 7 days. Scale bars, 100 μM. **(D)** Thyroid tissue weight of Tg-rtTA/tetO-BRAF^{V600E} mice fed with DOX versus normal chow for 7 days (n = 3 per group). **(E)** Relative mRNA expression of NIS, TPO and TG in thyroid tissue of Tg-rtTA/tetO-BRAF^{V600E} mice fed with DOX versus normal chow for 7 days (n = 3 per group). **(F)** Representative images of Ki67 staining in thyroid tissue from Tg-rtTA/tetO-BRAF^{V600E} mice fed with DOX (*upper*) versus normal (*lower*) chow for 7 days. Scale bars, 100 μM. **(G)** Same as (F) but using an isotype control antibody. **(H)** Overview of *in vivo* study to determine the impact of Cu(DDC)$_2$ given by IV administration at 5 mg/kg dose on thyroidal NIS function in Tg-rtTA/tetO-BRAF^{V600E} mice. **(I)** $^{99m}TcO4^-$ uptake in salivary glands and liver harvested from DOX chow fed Tg-rtTA/tetO-BRAF^{V600E} mice as outlined in (H) and administered with Cu(DDC)$_2$-ALB at dose of 5 mg/kg versus vehicle (n = 3-6 per group). Total animals used = 9. **(J)** Same conditions as (I) but showing body weight of Tg-rtTA/tetO-BRAF^{V600E} mice treated with CU(DDC)$_2$-ALB versus vehicle. Data presented as mean ± S.E.M; one-way ANOVA followed by Tukey's post hoc test (ns, not significant; $^{***}P < 0.001$); unpaired two-tailed t-test (ns, not significant; $^{###}P < 0.001$). Images created with BioRender.com.

SUPPLEMENTARY FIGURE S10

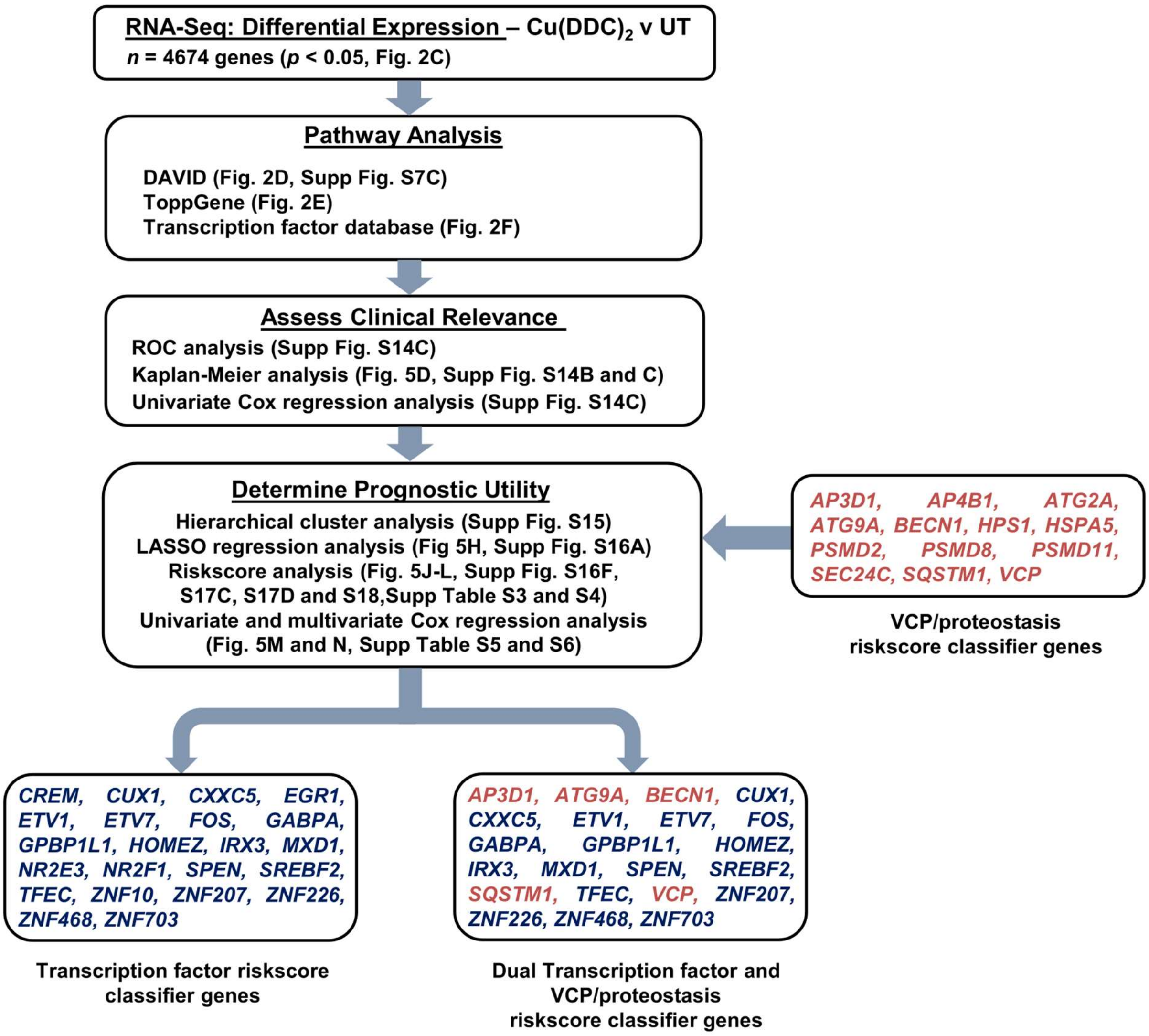

Fig. S10: **Overview of construction of riskscore classifiers for PTC recurrence.** Summary of bioinformatic pipeline used to identify potential clinical biomarkers for PTC recurrence and construction of (i) a transcription factor (TF) riskscore (22 genes, blue text) and (ii) a dual TF and VCP/proteostasis riskscore classifier (22 genes, blue and red text).

SUPPLEMENTARY FIGURE 11

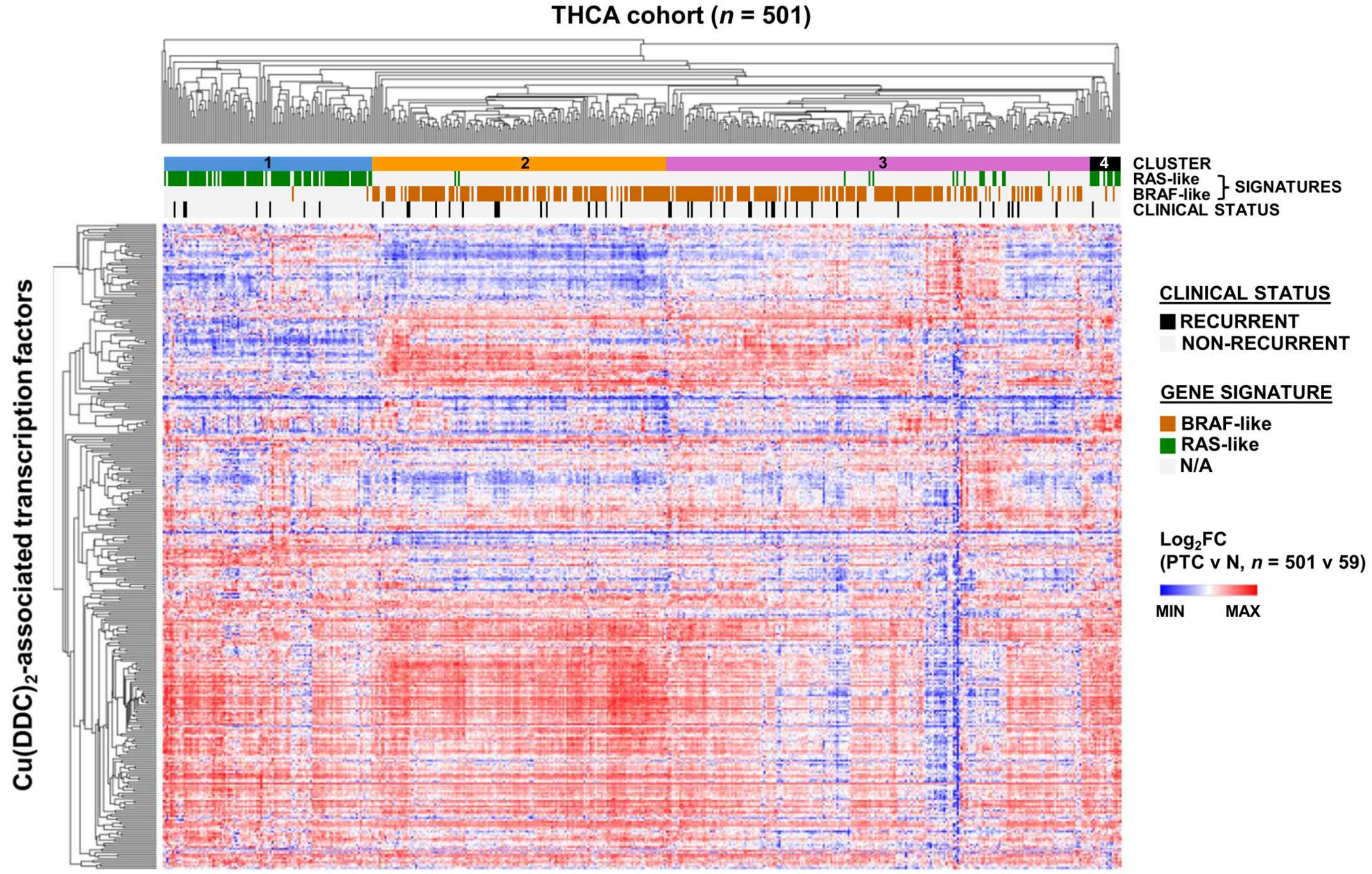

Fig. S11: **Hierarchical cluster analysis of $Cu(DDC)_2$-perturbed transcription factors in thyroid cancer.** Hierarchical cluster analysis of the thyroid cancer TCGA cohort (THCA, n = 501) based on log_2FC (PTC versus N) of 337 $Cu(DDC)_2$-perturbed TF genes. PTC characteristics including clinical status (recurrent versus non-recurrent) and gene signature [BRAF-like, RAS-like or not available (N/A)] are indicated.

SUPPLEMENTARY FIGURE 12

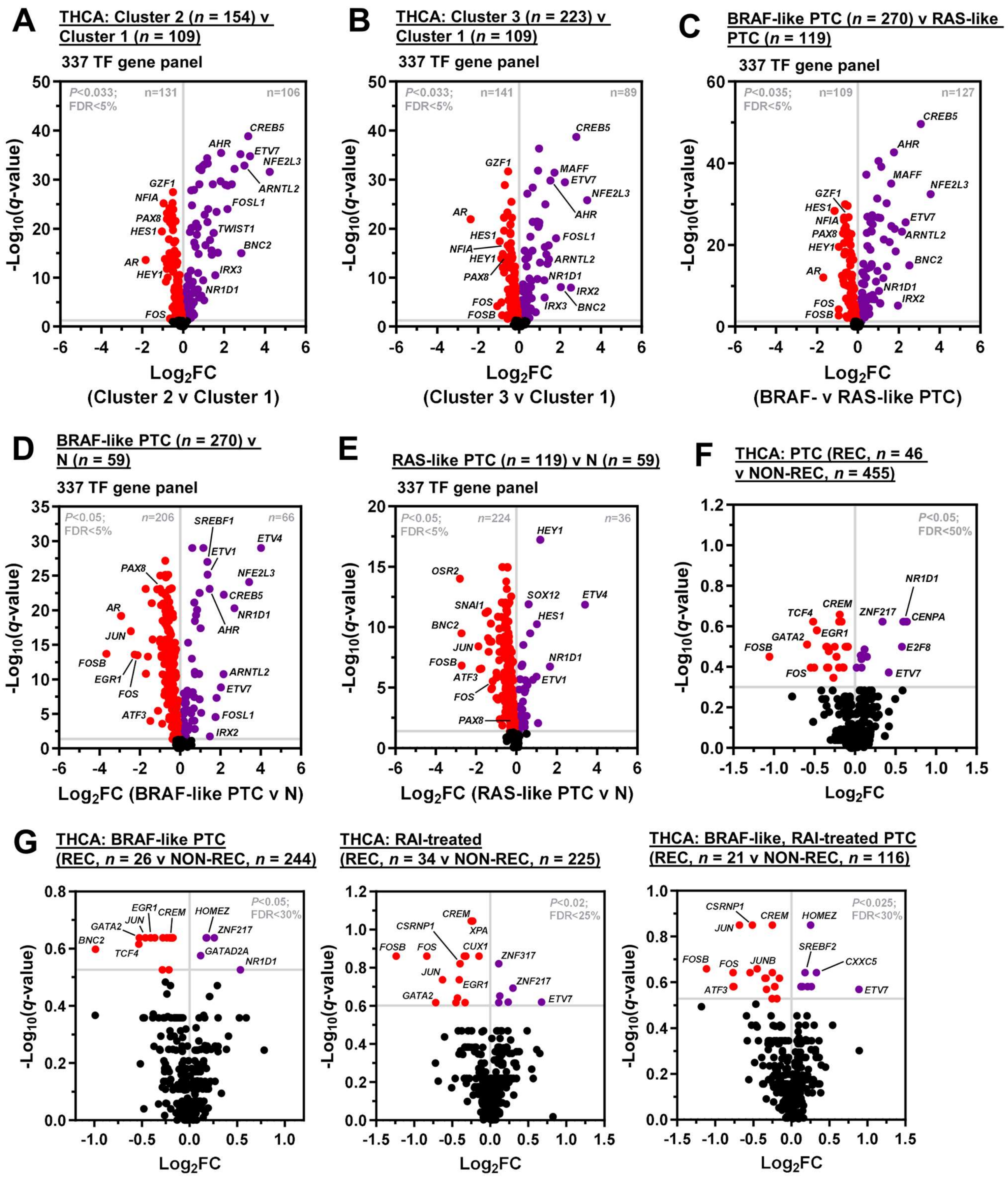

314
315

Fig. S12: **Identification of Cu(DDC)$_2$-perturbed transcription factors linked with PTC recurrence. (A)** Volcano plot comparing log$_2$FC for 337 TF genes in THCA cohorts stratified by hierarchical cluster analysis [cluster 1 (blue) versus cluster 2 (orange); Supplementary Fig. S11]. **(B)** Same as (A) but comparing THCA hierarchical cluster 1 (blue) versus cluster 3 (pink) cohorts. **(C-E)** Volcano plots comparing log$_2$FC with *q*-value (-log base 10) for 337 TF genes in THCA cohorts - (C) BRAF-like PTC versus RAS-like PTC, (D) BRAF-like PTC versus normal (N) and (E) RAS-like PTC versus N. **(F)** Volcano plot comparing log$_2$FC with q-value (-log base 10) for 337 TF genes in the entire THCA cohort [recurrent (REC) versus non-recurrent (NON-REC)]. **(G)** Same as (F) but in the BRAF-like PTC (*left*), RAI-treated (*middle*) or BRAF-like, RAI-treated PTC (*right*) cohort [REC versus NON-REC].

SUPPLEMENTARY FIGURE 13

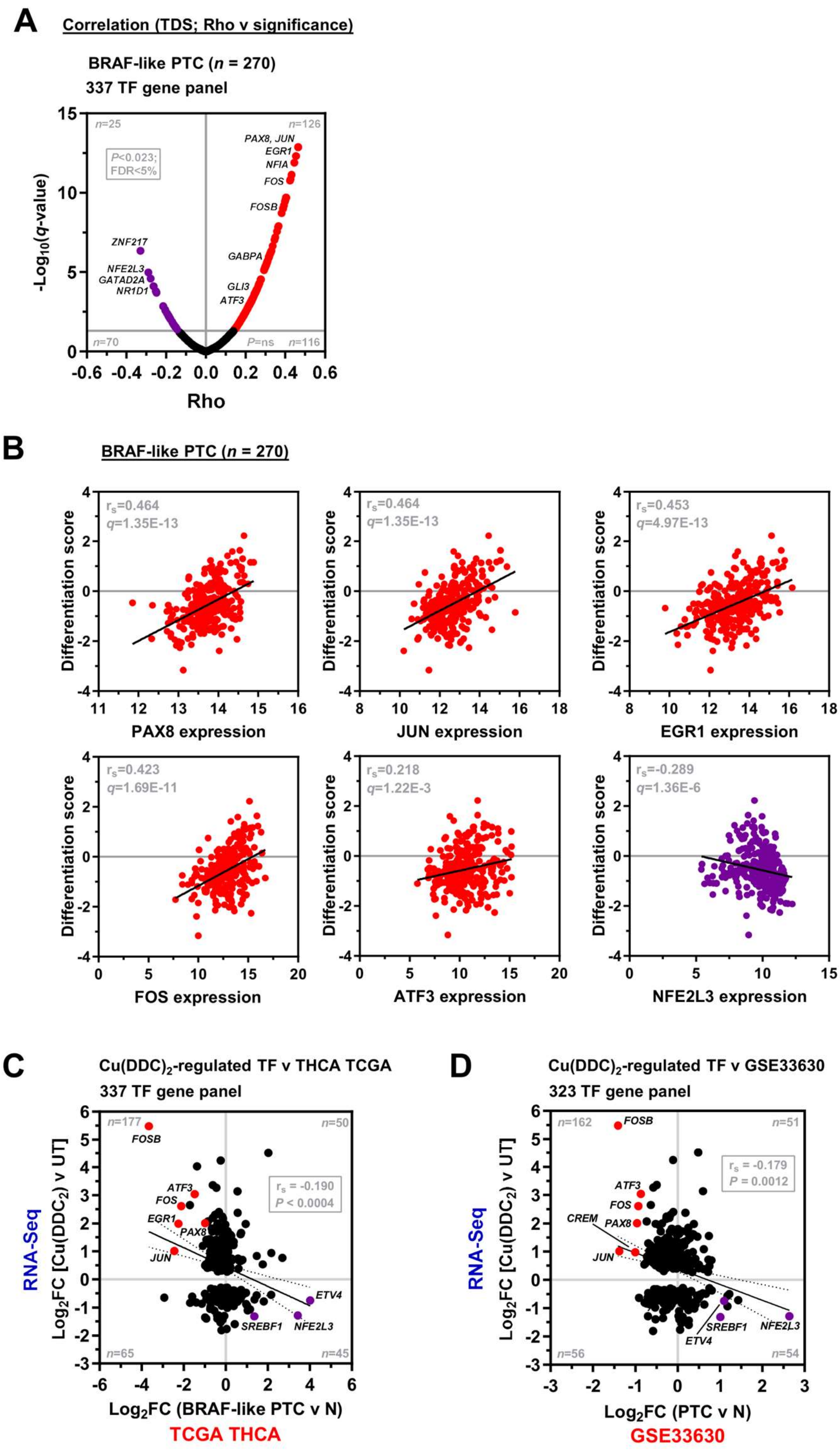

Fig. S13: **Molecular profiling identifies a putative redifferentiation effect of $Cu(DDC)_2$. (A)** Volcano plot illustrating Spearman's correlation coefficient rho with q-value (-log base 10) for TF gene expression (n = 337) versus thyroid differentiation score (TDS) in the BRAF-like PTC cohort. **(B)** Scatter plots showing correlation between differentiation score and TCGA expression for 6

representative genes in the BRAF-like PTC cohort; r_s - Spearman's rank coefficient. **(C)** Scatterplot showing correlation between RNA-Seq [log_2FC, $Cu(DDC)_2$ versus UT; Fig. 3D] and THCA (log_2FC, BRAF-like PTC versus N) expression data for 337 TF genes; r_s - Spearman's rank coefficient. **(D)** Same as (c) but showing correlation with 323 TF genes in the GSE33630 dataset (PTC versus N).

SUPPLEMENTARY FIGURE S14

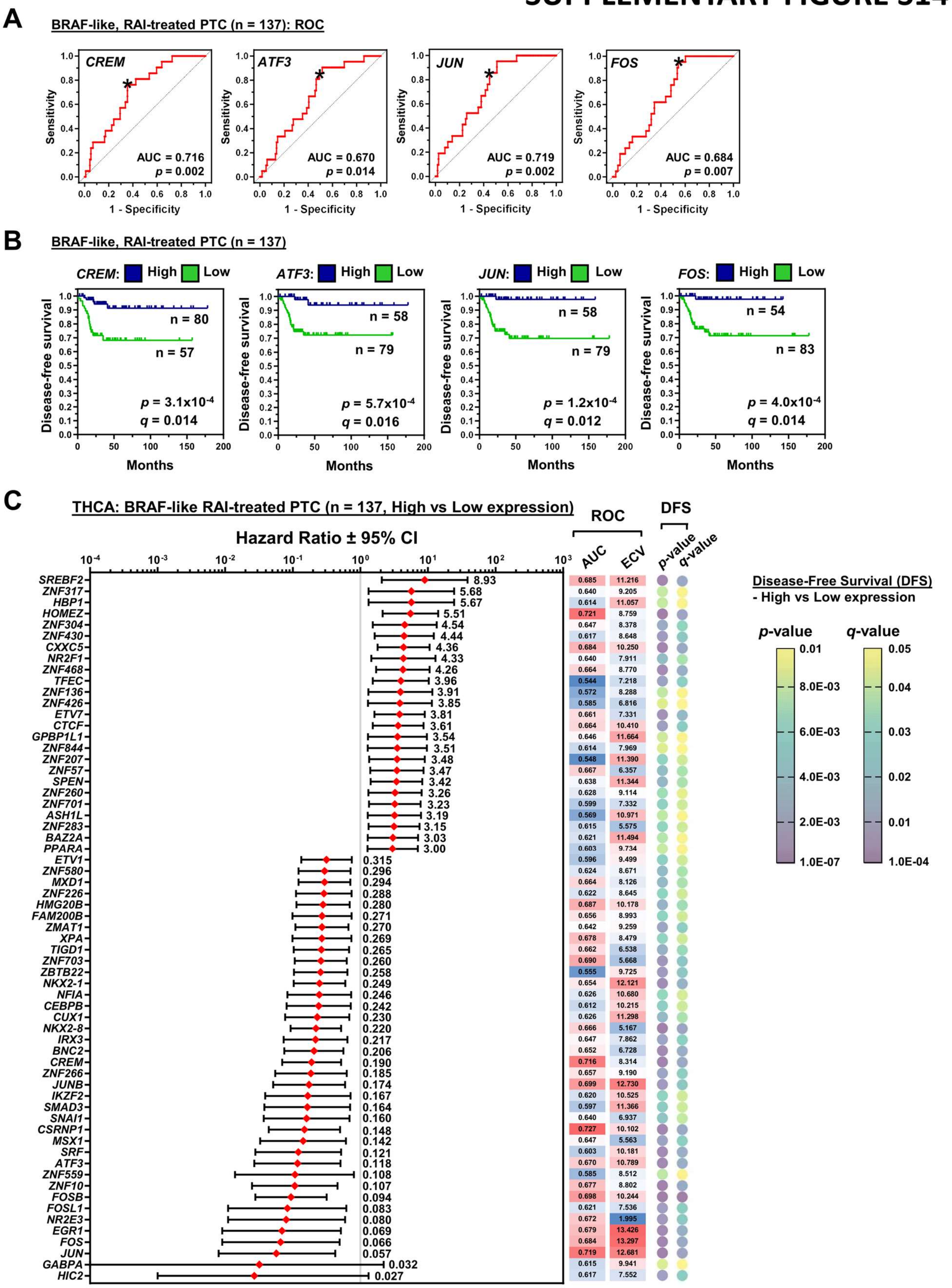

Fig. S14: **Clinically relevant Cu(DDC)$_2$-perturbed transcription factors in recurrent PTC. (A)** Receiver operating characteristic (ROC) curves of 4 representative transcription factor (TF) genes (*CREM*, *ATF3*, *JUN* and *FOS*) in BRAF-like, RAI-treated PTC. **(B)** Representative Kaplan-Meier analysis of disease-free survival (DFS) in BRAF-like, RAI-treated PTC stratified on high versus low tumoural expression of *CREM*, *ATF3, JUN* and *FOS*; log-rank test. Number (n) of patients per sub-group (high/low), as well as *p*- and *q*-values are shown. **(C)** Univariate Cox regression, ROC and Kaplan-Meier analysis of 63 TF genes in BRAF-like, RAI-treated PTC (n = 137). AUC, area under curve; ECV; expression cut-off value used for patient cohort stratification; CI, confidence interval. *P*- and *q*-values are shown.

SUPPLEMENTARY FIGURE S15

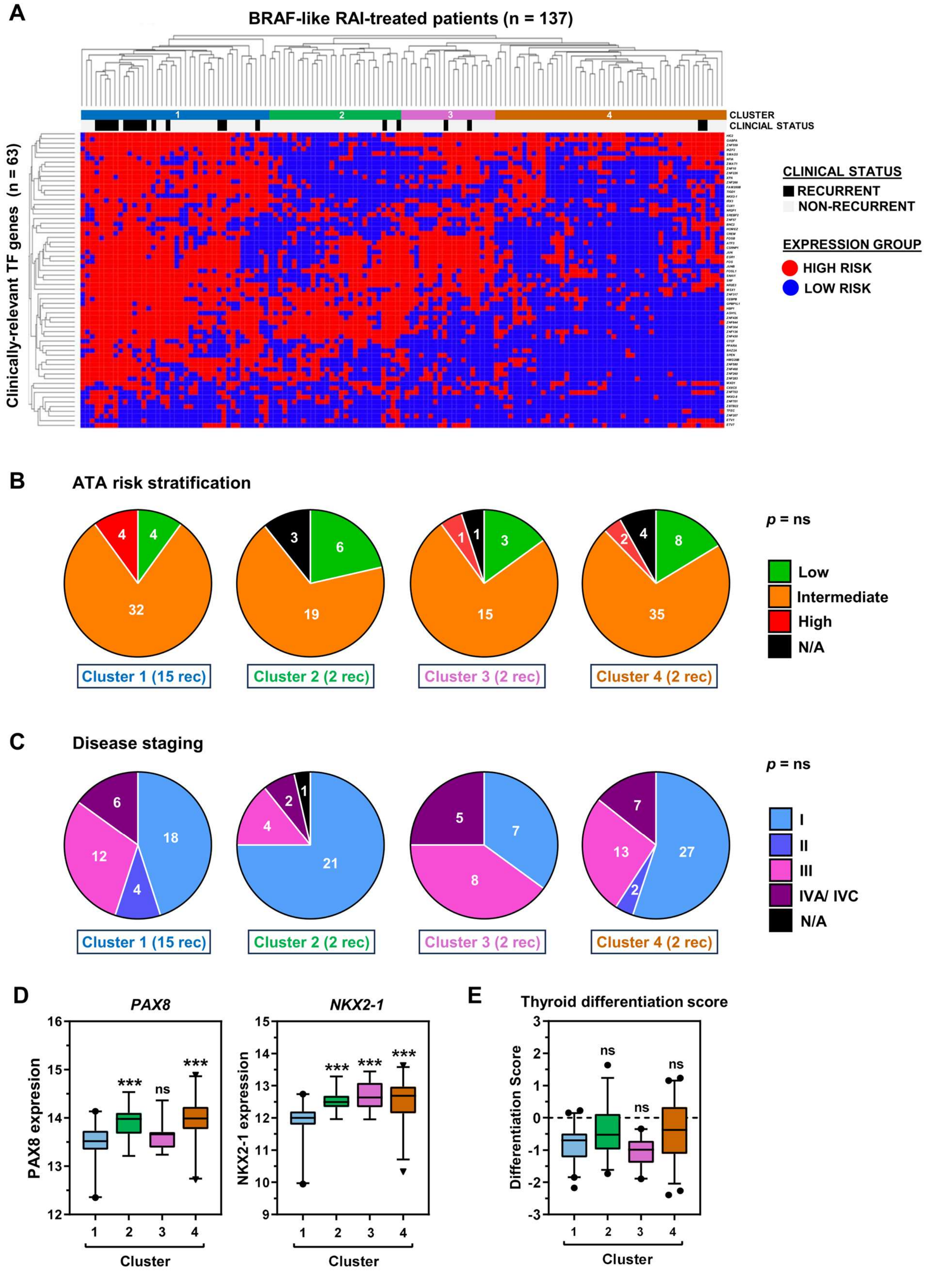

***Fig. S15:* Hierarchical cluster analysis of transcription factors with recurrence in RAI-treated PTC. (A)** Hierarchical cluster analysis based on expression of transcription factor (TF) genes (n = 63) stratified into high (red) and low (blue) risk groups in BRAF-like, RAI-treated PTC (n = 137). Four major clusters (1, blue; 2, green; 3, pink; 4, brown) and clinical status (recurrent, non-recurrent) are indicated. **(B)** Pie charts showing the ATA risk stratification characteristics of BRAF-like, RAI-treated patients subdivided into different clusters (1-4). Rec: number of recurrences. **(C)** Same as (B) but instead showing disease staging characteristics as indicated. *p*-values derived using Chi-Squared test; ns: not significant. **(D)** Box and whisker plots showing expression (log_2) of *PAX8* and *NKX2-1* in BRAF-like, RAI-treated PTC stratified into patient clusters 1 to 4; Kruskal-Wallis test, Dunn's post hoc test (ns, not significant; ***$P < 0.001$). **(E)** Box and whisker plot showing thyroid differentiation score (TDS) of BRAF-like, RAI-treated patients stratified into 4 clusters (1-4); Kruskal-Wallis test, Dunn's post hoc test (ns, not significant).

SUPPLEMENTARY FIGURE S16

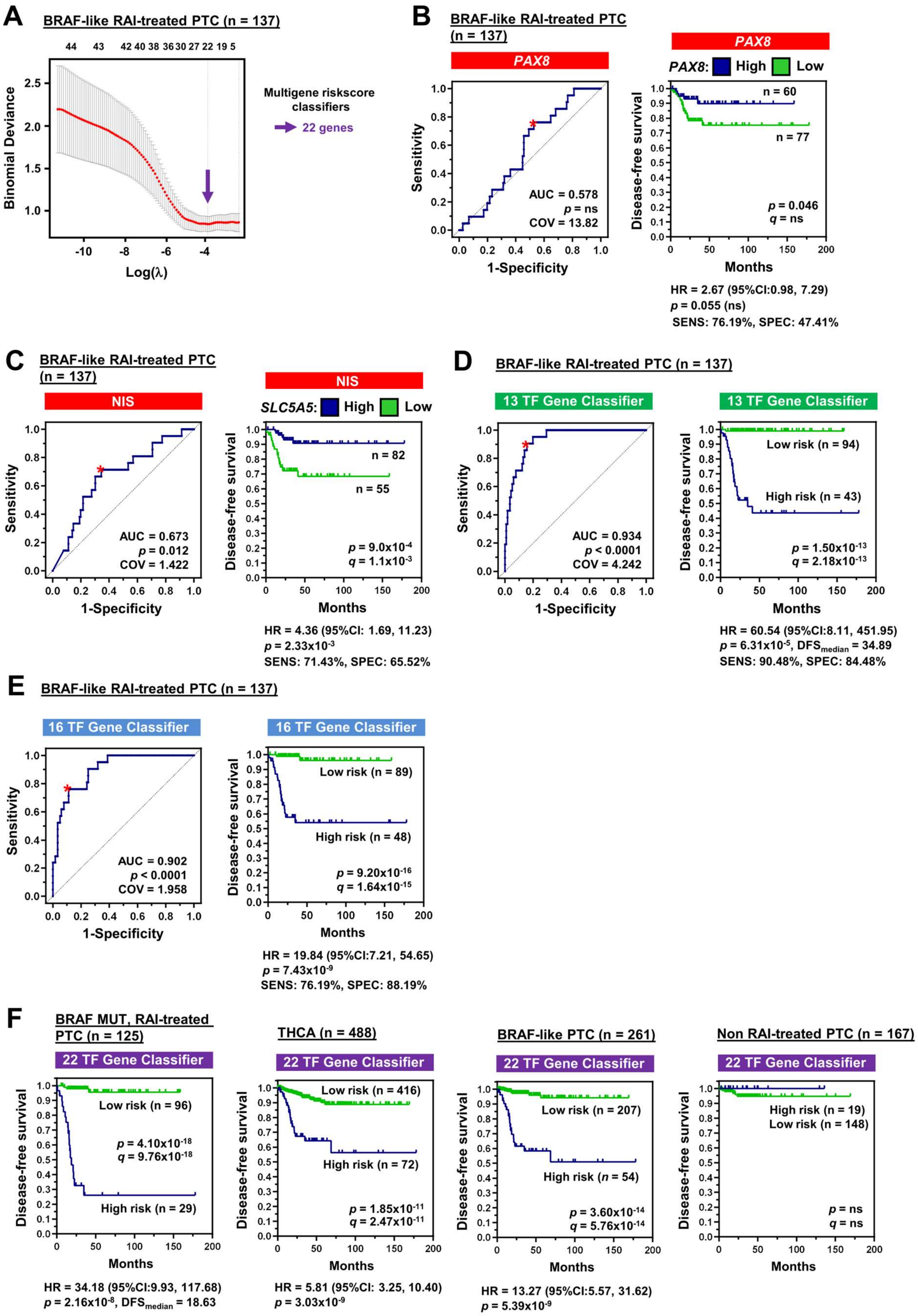

Fig. S16: **Construction of PTC riskscore classifiers. (A)** LASSO regression analysis of 63 transcription factor (TF) genes in BRAF-like RAI-treated PTC (n = 137). Cross validation plot of binomial deviance versus log (λ); minimal model deviation (left dotted line); minimal model deviation plus one standard error (right dotted line). Arrows highlight construction of 3 multigene riskscore classifiers based on 13, 16 and 22 TF genes. **(B** and **C)** ROC analysis (*left*) and Kaplan-Meier curve (*right*) for BRAF-like, RAI-treated PTC stratified on high versus low PAX8 (B) or NIS (C) expression. Number (n) of patients per expression group, *p*- and *q*-values are shown. **(D)** ROC analysis (*left*) and Kaplan-Meier curve (*right*) of the 13 TF gene riskscore classifier in BRAF-like, RAI-treated PTC. Construction of riskscore same as (A) except 13 genes filtered using LASSO regression from top 20 candidate TF genes. **(E)** Same as (D) except 16 genes filtered using LASSO regression from top 40 candidate TF genes. **(F)** Representative Kaplan-Meier analysis of DFS in BRAF MUT RAI-treated PTC (n = 125), entire THCA PTC cohort (n = 488), BRAF-like PTC (n = 261) and non RAI-treated PTC (n = 167) stratified using the 22 TF gene riskscore classifier: log-rank test. Number (n) of patients per group (high/low risk), as well as *P*- and *q*-values are shown.

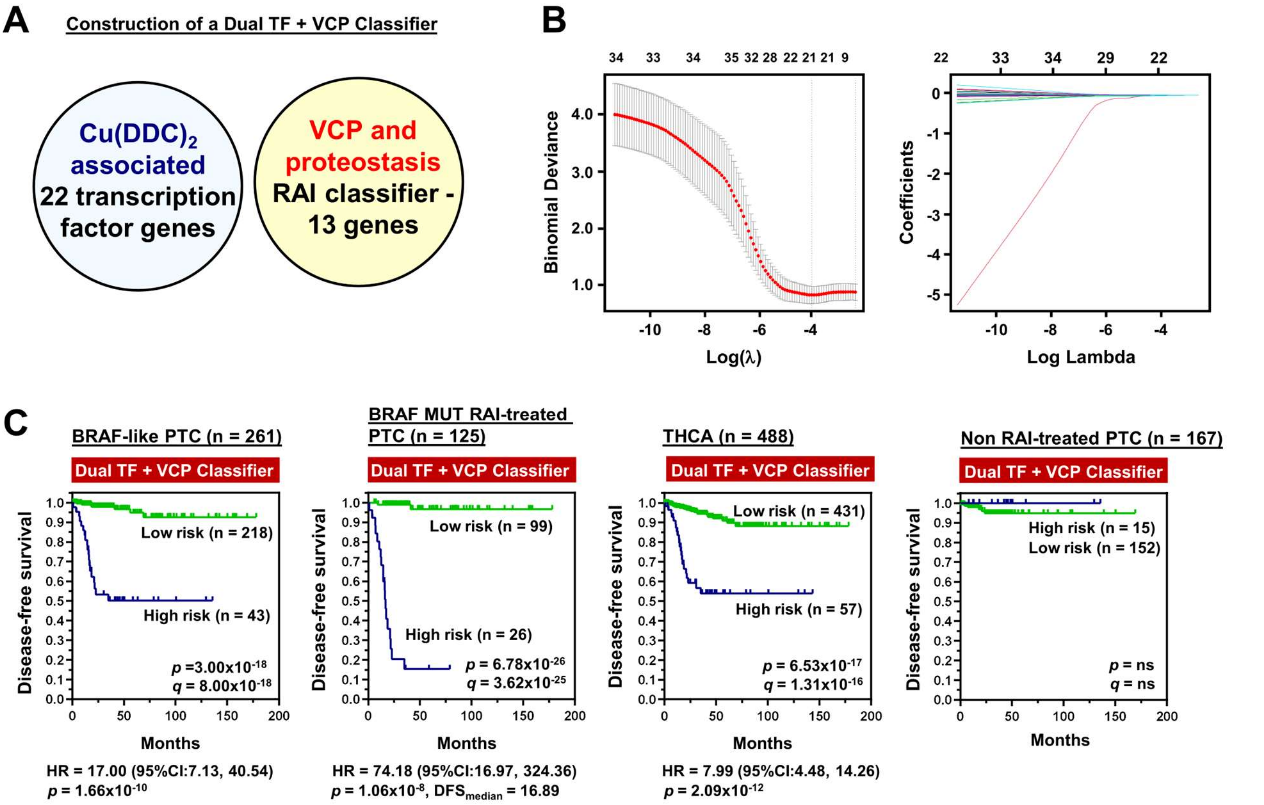

D BRAF-like RAI-treated PTC (n = 137)

Gene Classifier	No. of Genes	Cut-off value	Sensitivity (%)	Specificity (%)	AUC	HR	95%CI	PPV (%)	NPV (%)
Dual TF + VCP	22	10.63	90.48	94.83	0.954	84.43	19.34-368.53	76.0	98.2
TF	22	1.03	85.71	91.38	0.951	39.06	11.36-134.30	64.3	97.2
VCP/proteostasis	13	12.60	95.24	69.83	0.842	35.86	4.81-267.41	36.4	98.8
TF	13	4.24	90.48	84.48	0.934	60.54	8.11-451.95	46.5	98.9
TF	16	1.96	76.19	88.19	0.902	19.84	7.21-54.65	39.6	97.8
NIS	1	1.42	71.43	65.52	0.673	4.36	1.69-11.23	27.3	92.7
PAX8	1	13.82	76.19	47.41	0.578	2.67	0.98-7.29	20.8	91.7

Fig. S17: **Enhanced prognostic characteristics using a dual transcription factor (TF) and VCP/proteostasis riskscore classifier. (A** and **B)** LASSO regression analysis of combined 22 TF and 13 VCP/proteostasis genes in BRAF-like RAI-treated PTC (n = 137). *Left*: Cross validation plot of binomial deviance versus log (λ); minimal model deviation (left dotted line); minimal model deviation plus one standard error (right dotted line). *Right*: LASSO coefficient plot (loglambda). Y-axis: coefficient value; x-axis (lower): log(λ) value, and x-axis (upper): gene number. **(C)** Representative Kaplan-Meier analysis of DFS for BRAF-like, RAI-treated PTC (n = 137), BRAF-like PTC (n = 261), BRAF MUT RAI-treated PTC (n = 125), entire THCA cohort (n = 488) and non RAI-treated PTC (n = 167) stratified using the dual TF and VCP/proteostasis riskscore classifier; log-rank test. Number (n) of

patients per group (high/low risk), as well as p- and q-values are shown. **(D)** Comparative overview of prognostic characteristics of riskscore classifiers used in study. Details shown include number of genes, cut-off value (COV), sensitivity (%), specificity (%), area under the curve (AUC), hazard ratio (HR), confidence intervals (95% CI), % positive predictive value (PPV) and % negative predictive value (NPV).

SUPPLEMENTARY FIGURES S18

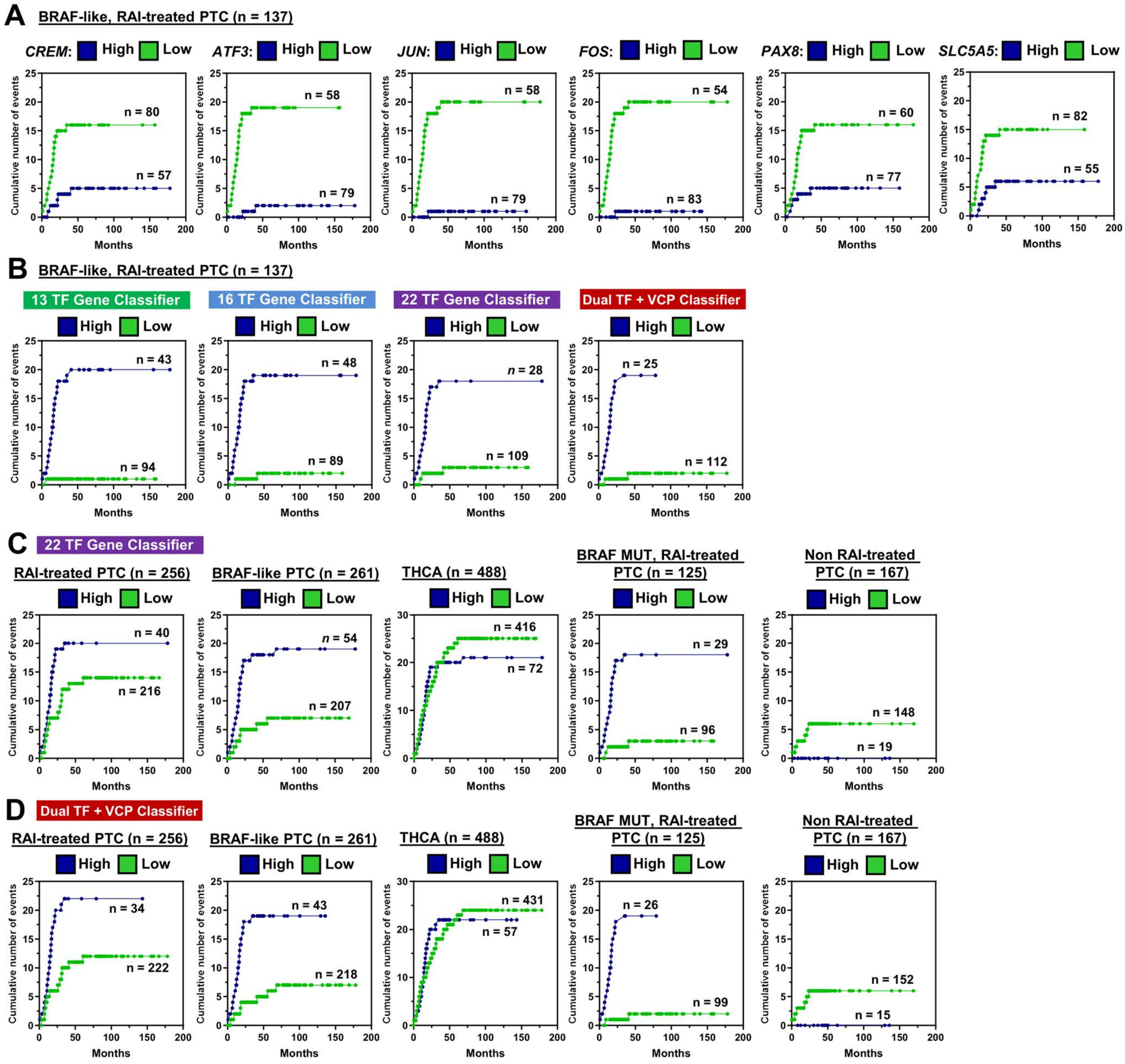

Fig. S18: **Visualisation of total number of recurrent events over time using different predictors.** **(A)** Cumulative recurrent event plots in BRAF-like, RAI-treated PTC stratified on high versus low tumoural expression of CREM, ATF3, JUN, FOS, PAX8 and SLC5A5. Number (n) of patients per sub-group. **(B)** Same as (A) but patients stratified into high versus low risk groups using the 13 transcription factor (TF), 16 TF, 22 TF or dual TF + VCP/proteostasis riskscore classifier. **(C)** Same as (B) but using the 22 TF gene classifier to stratify patients in different THCA cohorts as indicated. **(D)** Same as (C) but using the dual TF + VCP/proteostasis classifier to stratify patients into high versus low risk groups.

SUPPLEMENTARY FIGURE S19

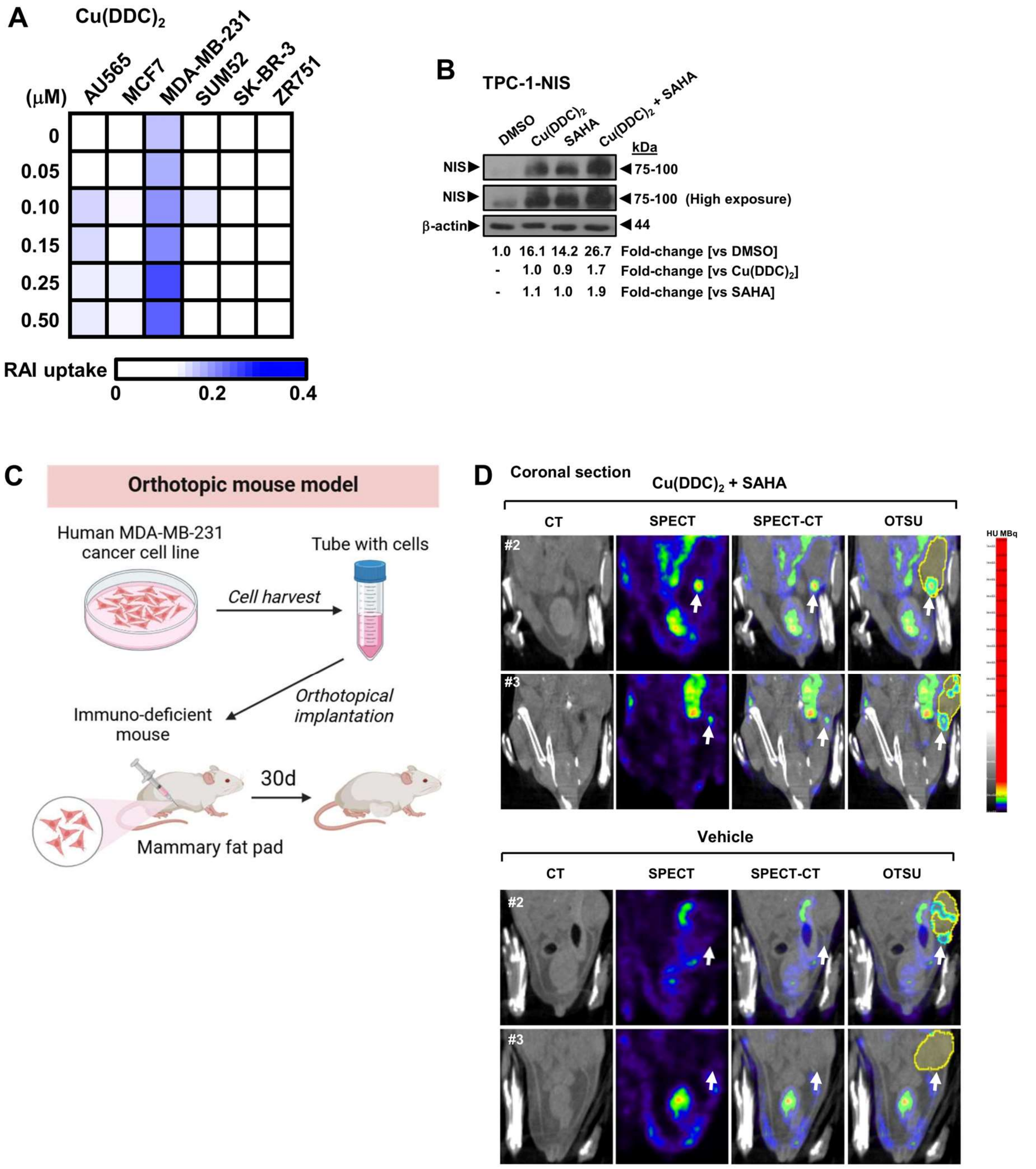

Fig. S19: **Combinatorial drug strategy to enhance NIS activity in breast cancer. (A)** Heatmap showing relative RAI uptake across a panel of breast cancer cell lines (AU565, MCF7, MDA-MB-231, SUM-2, SK-BR-3, ZR751) treated with $Cu(DDC)_2$ at indicated doses. **(B)** Western blot analysis of NIS protein in TPC1-NIS cells treated with $Cu(DDC)_2$, SAHA or combination versus vehicle. *Below*: image quantification of NIS protein levels along with relative fold differences between drug treatments. **(C)** Schematic illustrating generation of an orthotopic MDA-MB-231 breast cancer mouse model. Created in BioRender.com. **(D)** SPECT/CT images of technetium-99m (^{99m}Tc) uptake in parental

MDA-MB-231 mammary pad xenografts. Mice were treated with $Cu(DDC)_2$ + SAHA (*upper*) or vehicle (*lower*) as indicated. Images: CT, SPECT, SPECT-CT and SPECT-CT with OTSU thresholding analysis of tumour region. Relative location of tumour indicated (white arrow).

SUPPLEMENTARY TABLE S1

Clotrimazole (CLOT)

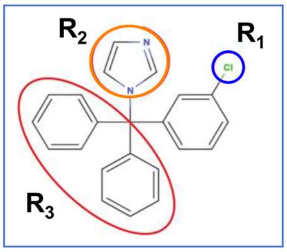

Clotrimazole analogues	Modifications[1]			LogP
	R_1	R_2	R_3	
CLOT	Cl	$C_3H_4N_2$	$(C_6H_6)_2$	5.37
C1	COOH	$C_3H_4N_2$	$(C_6H_6)_2$	4.07
C2	$CONH_2$	$C_3H_4N_2$	$(C_6H_6)_2$	4.67
C3	OH	$C_3H_4N_2$	$(C_6H_6)_2$	4.69
C4	$COOCH_3$	$C_3H_4N_2$	$(C_6H_6)_2$	4.67
C5	COC_4H_9NO	$C_3H_4N_2$	$(C_6H_6)_2$	4.44
C6	No R_1 group	$C_3H_4N_2$	$(C_6H_6)_2$	4.76
C7	CH_2OH	$C_3H_4N_2$	$(C_6H_6)_2$	4.16
C8	Cl	$C_3H_6N_2^+ + Cl^-$	$(C_6H_6)_2$	1.31
C9	Cl	$C_3H_4N_2$	No R_3 group	1.91
C10	Cl	$C_3H_4N_2NO_2$	$(C_6H_6)_2$	4.62
C11	Cl	$C_3H_6N_2^+ + Cl^-$	No R_3 group	-2.14
C12	Cl	$C_7H_6N_2$	$(C_6H_6)_2$	6.81
C13	Cl	$C_2H_3N_3$	$(C_6H_6)_2$	4.78
C14	Cl	$C_4H_4N_3O$	$(C_6H_6)_2$	5.42
C15	No R_1 group	$C_3H_6N_2^+ + Cl^-$	No R_3 group	-2.14
C16	Cl	$C_3H_2N_3O_2$	No R_3 group	1.18
C17	F	$C_3H_6N_2^+ + Cl^-$	No R_3 group	-2.64
C18	F+F on C_2 of benzyl ring	$C_3H_6N_2^+ + Cl^-$	No R_3 group	-2.55
C19	No R_1 group	$C_3H_6N_2^+ + Cl^-$	No R_3 group	-2.75
C20	Cl	$C_3H_4N_2$	CO	2.31

Supp. Table S1: **Clotrimazole analogues designed to enhance biological efficacy and bioavailability.** [1]20 clotrimazole analogues (C1 to C20) are listed with structural modifications made at the R_1, R_2 and R_3-groups; unmodified clotrimazole: CLOT. Calculated LogP values are also given. (*left*) Chemical structure of clotrimazole highlighting modifications made at the chloro-substituted aryl ring (R1; blue), imidazole ring (R2; orange) and aryl substituent groups (R3; red).

SUPPLEMENTARY TABLE 2

Rank	Copper/Zinc related drugs	*q*-value	Hit count	ID	Source
1	APTT	1.21E-101	146	C517041	CTD
2	PCI 5002	7.609E-90	163	C568608	CTD
5	Cupric oxide	1.863E-51	107	C030973	CTD
9	Elesclomol	1.109E-40	36	C512195	CTD
17	NSC 689534	2.459E-32	148	C558013	CTD
65	Cupric chloride	3.534E-17	68	C029892	CTD

Rank	VCP/Proteasomal inhibitors	*q*-value	Hit count	ID	Source
4	Disulfiram	1.866E-76	154	D004221	CTD
8	MG262	8.802E-44	59	7068_UP	Broad Institute CMAP Up
14	MG132	6.371E-33	50	1140_UP	Broad Institute CMAP Up
23	Terfenadine	1.081E-29	47	2227_UP	Broad Institute CMAP Up
34	Astemizole	3.054E-25	42	2211_UP	Broad Institute CMAP Up
77	Clotrimazole	1.848E-15	35	5726_UP	Broad Institute CMAP Up

Supp. Table S2: **Top drug: gene associations with VCP/proteasomal inhibitors and copper-related drugs in Cu(DDC)$_2$-treated cells.** ToppGene classification of drug-gene associations in 771 differentially expressed genes ($log_2FC > 1$, $p < 0.05$, FDR < 13%) in parental 8505C cells treated with 0.25 µM Cu(DDC)$_2$. Top 6 drugs are shown with strongest associations to differentially expressed genes in the categories of "Copper/Zinc related drugs" and "VCP/Proteasomal inhibitors". Abbreviations: CTD, Comparative Toxicogenomics Database; CMAP, Connectivity Map, APTT, (4-amino-1,4-dihydro-3-(2-pyridyl)-5-thioxo-1,2,4-triazole)copper(II).

SUPPLEMENTARY TABLE S3

(i) Transcription Factor 13 Gene Risk Score Classifier: LASSO Regression Coefficients

Gene	Coefficient
BNC2	0.000680413
CREM	-0.002761923
CXXC5	0.001734669
EGR1	-5.66919E-05
ETV7	0.004060528
FOS	-5.90495E-06
HOMEZ	0.005996233
NR2E3	-0.003392
SREBF2	0.000200779
SRF	0.001028347
ZNF10	-0.002769783
ZNF468	0.001982434
ZNF703	-0.019887234

(ii) Transcription Factor 16 Gene Risk Score Classifier: LASSO Regression Coefficients

Gene	Coefficient
CREM	-0.000707965
CUX1	-0.00035432
CXXC5	0.001053119
EGR1	-1.97328E-05
ETV7	0.001605858
FOS	-1.78995E-06
HOMEZ	0.004319221
JUNB	-3.61043E-07
MXD1	-0.000497921
NR2F1	8.31242E-05
SPEN	4.83122E-06
SREBF2	9.56941E-05
TFEC	0.001182093
ZNF10	-0.001036329
ZNF468	0.000721357
ZNF703	-0.008611942

(iii) VCP/Proteostasis 13 Gene Risk Score Classifier: Regression Coefficients

Gene	Coefficient
AP3D1	0.000088
AP4B1	0.002892
ATG2A	-0.000932
ATG9A	0.001931
BECN1	0.002862
HSPA5	-0.000125
HPS1	0.000045
PSMD2	-0.001065
PSMD8	0.000185
PSMD11	0.002498
SEC24C	0.002052
SQSTM1	0.000036
VCP	0.000265

(iv) Transcription Factor 22 Gene Risk Score Classifier: LASSO Regression Coefficients

Gene	Coefficient
CREM	-0.000130599
CUX1	-0.000470708
CXXC5	0.001234149
EGR1	-1.29613E-05
ETV1	-0.000675338
ETV7	0.001420873
FOS	-3.55568E-06
GABPA	-0.001734825
GPBP1L1	2.21377E-05
HOMEZ	0.004487796
IRX3	-0.000136032
MXD1	-0.002122027
NR2E3	-0.000268855
NR2F1	5.05628E-05
SPEN	0.000237215
SREBF2	0.000101241
TFEC	0.00204131
ZNF10	-0.000866429
ZNF207	0.000796842
ZNF226	-0.00244898
ZNF468	0.001136406
ZNF703	-0.017290483

(v) Dual Transcription Factor + VCP/Proteostasis 22 Gene Risk Score Classifier: LASSO Regression Coefficients

Gene	Coefficient
AP3D1	3.34752E-05
ATG9A	0.001315281
BECN1	0.001077816
CUX1	-0.000726222
CXXC5	0.001227282
ETV1	-0.000783195
ETV7	0.00050079
FOS	-1.7761E-05
GABPA	-0.000691614
GPBP1L1	0.000130766
HOMEZ	0.005644972
IRX3	-0.000203417
MXD1	-0.002508809
SPEN	0.000357002
SQSTM1	2.03804E-05
SREBF2	6.16728E-05
TFEC	0.00348904
VCP	0.000457048
ZNF207	0.000818327
ZNF226	-0.001111371
ZNF468	0.000817046
ZNF703	-0.017715603

Supp. Table S3: **Multigene riskscore classifiers used to predict PTC recurrence.**

SUPPLEMENTARY TABLE S4

Clinical Variable	BRAF-like, RAI-treated PTC	BRAF-like PTC	RAI-treated PTC	TCGA THCA	*P*-value
n	137	261	256	488	
**Age**					
Median (IQR)	43.0 (33.5-56.5)	46.0 (33.5-56.0)	46.0 (35.0-58.0)	46.0 (34.0-57.0)	ns[1]
**Sex**					
Female (%)	70.80	75.1	68.75	73.36	ns[2]
**Disease stage**					
III + IV (%)	41.91	34.61	38.82	31.89	ns[2]
Missing values (n)	1	1	1	2	
**Risk group (n)**					
Low	21	70	56	168	< 0.0001[2]
Intermediate + High	108	172	180	274	
Missing values	8	19	20	46	
**Dual TF + VCP riskscore classifier**					
Median (IQR)	8.91 (7.72-10.14)	9.15 (7.82-10.14)	8.49 (7.05-9.80)	8.53 (7.05-9.75)	ns[1]
**Recurrence**					
n	21	26	34	46	ns[2]
%	15.33	9.96	13.28	9.45	
Missing values (n)	0	0	0	1	
**Signature (n)**					
BRAF-like	137	261	138	263	ND[3]
RAS-like	0	0	50	116	
Missing values	0	0	68	109	
**Treatment (n)**					
RAI	137	137	256	256	
Non-RAI	0	94	0	167	
Missing values	0	30	0	65	
**TN staging (n)**					
Yes	124	241	226	439	
Missing values	13	20	30	49	

Supp. Table S4: **Characterisation of PTC cohorts used in study.** Summary of key characteristics of different PTC cohorts, including sample size, age, sex, disease stage, risk group, dual TF + VCP riskscore classifier, outcome events (recurrence), reported molecular signatures (BRAF-like versus RAS-like), treatments received and TN staging. The number of missing values is also given. [1]Kruskal-Wallis test followed by Dunn's multiple comparisons test comparing BRAF-like, RAI-treated PTC to other 3 datasets (ns, not significant). [2]Fisher's exact test was used to determine significance between datasets (ns, not significant). [3]Not done (ND). Comparative analysis was not performed due to a high proportion of missing values (> 10%) which precluded a reliable comparison.

SUPPLEMENTARY TABLE S5

A

Clinical Variable	RAI-treated (*n* = 211)					THCA (*n* = 399)				
	n	Univariate *P*-value	Univariate HR (95% CI)	Multivariate *P*-value	Multivariate HR (95% CI)	*n*	Univariate *P*-value	Univariate HR (95% CI)	Multivariate *P*-value	Multivariate HR (95% CI)
**Age, years**										
< 50	127	0.058	2.007 (0.977-4.121)	0.289	2.089 (0.535-8.154)	233	0.070	1.780 (0.954-3.324)	0.978	1.013 (0.396-2.589)
> 50	84					166				
**Sex**										
Male	67	0.464	1.320 (0.628-2.775)	0.410	0.708 (0.312-1.608)	106	0.154	1.606 (0.838-3.078)	0.816	1.083 (0.551-2.129)
Female	144					293				
**Disease stage**										
I + II	122	0.055	2.032 (0.984-4.199)	0.954	1.043 (0.251-4.338)	267	**8.10E-04**	**2.911 (1.558-5.441)**	0.234	1.846 (0.673-5.061)
III + IV	89					132				
**Risk group**										
Low	40	0.055	7.058 (0.961-51.817)	0.272	3.341 (0.388-28.738)	137	**3.00E-03**	**4.841 (1.723-13.605)**	0.129	2.470 (0.769-7.933)
Intermediate + High	171					262				
**Dual TF + VCP riskscore classifier[1]**										
Low	182	**9.93E-15**	**21.345 (9.833-46.333)**	**3.09E-13**	**20.577 (9.126-46.397)**	351	**4.45E-12**	**8.979 (4.823-16.716)**	**1.08E-09**	**7.372 (3.879-14.011)**
High	29					48				

B

Clinical Variable	RAI-treated (*n* = 211)					THCA (*n* = 399)				
	n	Univariate *P*-value	Univariate HR (95% CI)	Multivariate *P*-value	Multivariate HR (95% CI)	*n*	Univariate *P*-value	Univariate HR (95% CI)	Multivariate *P*-value	Multivariate HR (95% CI)
**TF riskscore classifier[2]**										
Low	178	**1.17E-11**	**13.942 (6.512-29.852)**	**1.03E-10**	**13.214 (6.041-28.906)**	339	**2.53E-09**	**6.664 (3.571-12.435)**	**1.04E-06**	**5.021 (2.627-9.595)**
High	33					60				
**VCP/ proteostasis riskscore classifier[3]**										
Low	133	**1.60E-06**	**13.195 (4.600-37.854)**	**2.78E-06**	**12.882 (4.424-37.511)**	278	**1.06E-07**	**6.575 (3.283-13.166)**	**2.20E-06**	**5.479 (2.710-11.079)**
High	78					121				
**NIS classifier[4]**										
Low	130	**1.33E-03**	**3.468 (1.623-7.412)**	**1.45E-03**	**3.496 (1.618-7.555)**	264	**2.80E-04**	**3.278 (1.728-6.220)**	**5.10E-04**	**3.163 (1.652-6.059)**
High	81					135				

Supp. Table S5: **Dual transcription factor (TF) and VCP/proteostasis riskscore classifier is an independent predictor of recurrence in RAI-treated PTC. (A)** n, number; HR, hazard ratio; CI, confidence interval. $P < 0.05$ (bold) was considered statistically significant. Some patients in the RAI-treated PTC (n = 45) and entire TCGA THCA cohorts (n = 89) were not included in univariate and multivariate analysis of the THCA dataset due to missing clinical variables. [1]Patients stratified into high and low risk of recurrence using the dual TF and VCP/proteostasis riskscore classifier. Multivariate models were adjusted for the indicated covariates age, sex, disease stage and ATA risk group to control for potential confounding. **(B)** Same as (A) except the TF riskscore classifier, VCP/proteostasis riskscore classifier or NIS expression classifier were used instead of the dual TF and VCP/proteostasis riskscore classifier in multivariate analysis. [2]Patients stratified into high and low risk of recurrence using the 22 gene TF riskscore classifier. [3]Patients stratified into high and low risk of recurrence using the 13 gene VCP/proteostasis riskscore classifier. [4]Patients stratified based on high versus low tumoural NIS expression.

SUPPLEMENTARY TABLE S6

A

Clinical Variable	BRAF-like PTC (*n* = 222)					BRAF-like, RAI-treated PTC (*n* = 117)				
	n	Univariate *P*-value	Univariate HR (95% CI)	Multivariate *P*-value	Multivariate HR (95% CI)	*n*	Univariate *P*-value	Univariate HR (95% CI)	Multivariate *P*-value	Multivariate HR (95% CI)
**Age, years**										
< 50	128	0.094	2.027 (0.887-4.633)	0.38	0.486 (0.097-2.434)	73	0.090	2.179 (0.885-5.365)	0.339	0.257 (0.016-4.168)
> 50	94					44				
**Sex**										
Male	56	0.471	1.388 (0.569-3.383)	0.608	0.784 (0.309-1.986)	36	0.922	1.050 (0.398-2.764)	0.143	0.429 (0.138-1.333)
Female	166					81				
**Disease stage**										
I + II	143	**0.003**	**3.508 (1.517-8.116)**	0.084	4.235 (0.825-21.739)	64	0.150	1.954 (0.786-4.862)	0.084	12.106 (0.719-203.899)
III + IV	79					53				
**Risk group**										
Low	57	**0.038**	**8.305 (1.119-61.624)**	0.333	2.848 (0.342-23.694)	14	0.332	2.709 (0.361-20.305)	0.602	0.563 (0.065-4.864)
Intermediate + High	165					103				
**Dual TF + VCP riskscore classifier[1]**										
Low	186	**1.35E-09**	**17.904 (7.044-45.509)**	**3.49E-08**	**17.784 (6.394-49.463)**	96	**6.34E-09**	**84.149 (18.852-375.611)**	**8.43E-07**	**183.027 (23.021-1455.14)**
High	36					21				

B

Clinical Variable	BRAF-like PTC (*n* = 222)					BRAF-like, RAI-treated PTC (*n* = 117)				
	n	Univariate *P*-value	Univariate HR (95% CI)	Multivariate *P*-value	Multivariate HR (95% CI)	*n*	Univariate *P*-value	Univariate HR (95% CI)	Multivariate *P*-value	Multivariate HR (95% CI)
**TF riskscore classifier[2]**										
Low	177	**1.75E-08**	**14.628 (5.753-37.190)**	**1.25E-06**	**12.584 (4.521-35.031)**	94	**8.47E-09**	**42.046 (11.779-150.089)**	**9.85E-08**	**65.541 (14.076-305.176)**
High	45					23				
**VCP/ proteostasis riskscore classifier[3]**										
Low	141	**3.63E-05**	**12.904 (3.834-43.438)**	**1.71E-04**	**10.405 (3.068-35.291)**	68	**8.44E-04**	**30.916 (4.123-231-846)**	**1.02E-03**	**29.714 (3.927-224.816)**
High	81					49				
**NIS classifier[4]**										
Low	139	**2.36E-03**	**3.970 (1.632-9.656)**	**3.50E-03**	**3.832 (1.555-9.445)**	70	**8.22E-03**	**3.687 (1.401-9.706)**	**3.97E-03**	**4.299 (1.594-11.594)**
High	83					47				

Supp. Table S6: **Multivariate analysis of the dual transcription factor (TF) and VCP/proteostasis riskscore classifier in BRAF-like PTC. (A)** n, number; HR, hazard ratio; CI, confidence interval. $P < 0.05$ (bold) was considered statistically significant. Some patients in the BRAF-like PTC (n = 48) and BRAF-like, RAI-treated PTC cohorts (n = 20) were not included in univariate and multivariate analysis of the THCA dataset due to missing clinical variables. [1]Patients stratified into high and low risk of recurrence using the dual TF + VCP/proteostasis riskscore classifier. Multivariate models were adjusted for the indicated covariates age, sex, disease stage and ATA risk group to control for potential confounding. **(B)** Same as (A) except the TF riskscore classifier, VCP/proteostasis riskscore classifier or NIS expression classifier were used instead of the dual TF and VCP/proteostasis riskscore classifier in multivariate analysis. [2]Patients stratified into high and low risk of recurrence using the 22 gene TF riskscore classifier. [3]Patients stratified into high and low risk of recurrence using the 13 gene VCP/proteostasis riskscore classifier. [4]Patients stratified based on high versus low tumoural NIS expression.
