# Supplementary material for: Disulfiram metabolite Cu(DDC)2 enhances radionuclide uptake in vivo revealing insights into tumoural ablation resistance
Source: eBioMedicine. 2026 Feb 11;125:106165. doi: 10.1016/j.ebiom.2026.106165 (PMC12917385; doi:10.1016/j.ebiom.2026.106165)

Figure 1f

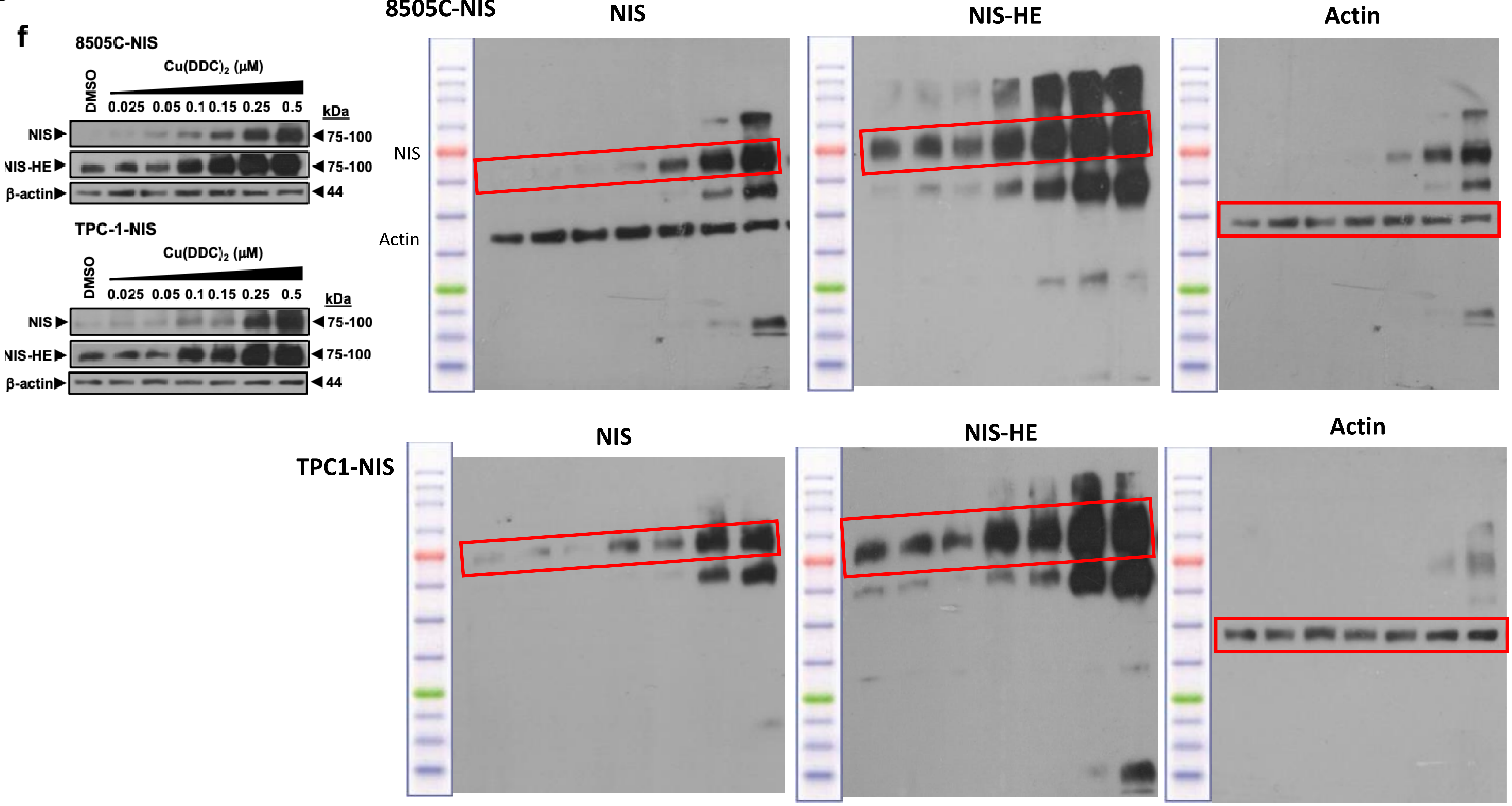

f
8505C-NIS
Cu(DDC)2 (μM)
DMSO
0.025 0.05 0.1 0.15 0.25 0.5
kDa
NIS
NIS-HE
β-actin
75-100
75-100
44
TPC-1-NIS
Cu(DDC)2 (μM)
DMSO
0.025 0.05 0.1 0.15 0.25 0.5
kDa
NIS
NIS-HE
β-actin
75-100
75-100
44
8505C-NIS
NIS
NIS-HE
Actin
NIS
Actin
TPC1-NIS
NIS
NIS-HE
Actin

Figure 1i

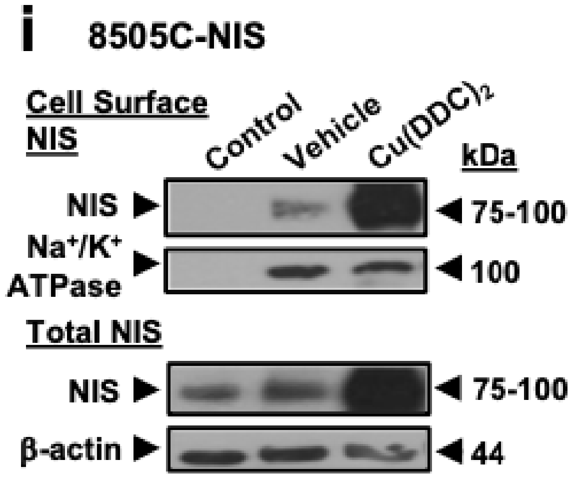

i 8505C-NIS
Cell Surface NIS
Control
Vehicle
Cu(DDC)₂
kDa
NIS
75-100
Na⁺/K⁺ ATPase
100
Total NIS
NIS
75-100
β-actin
44

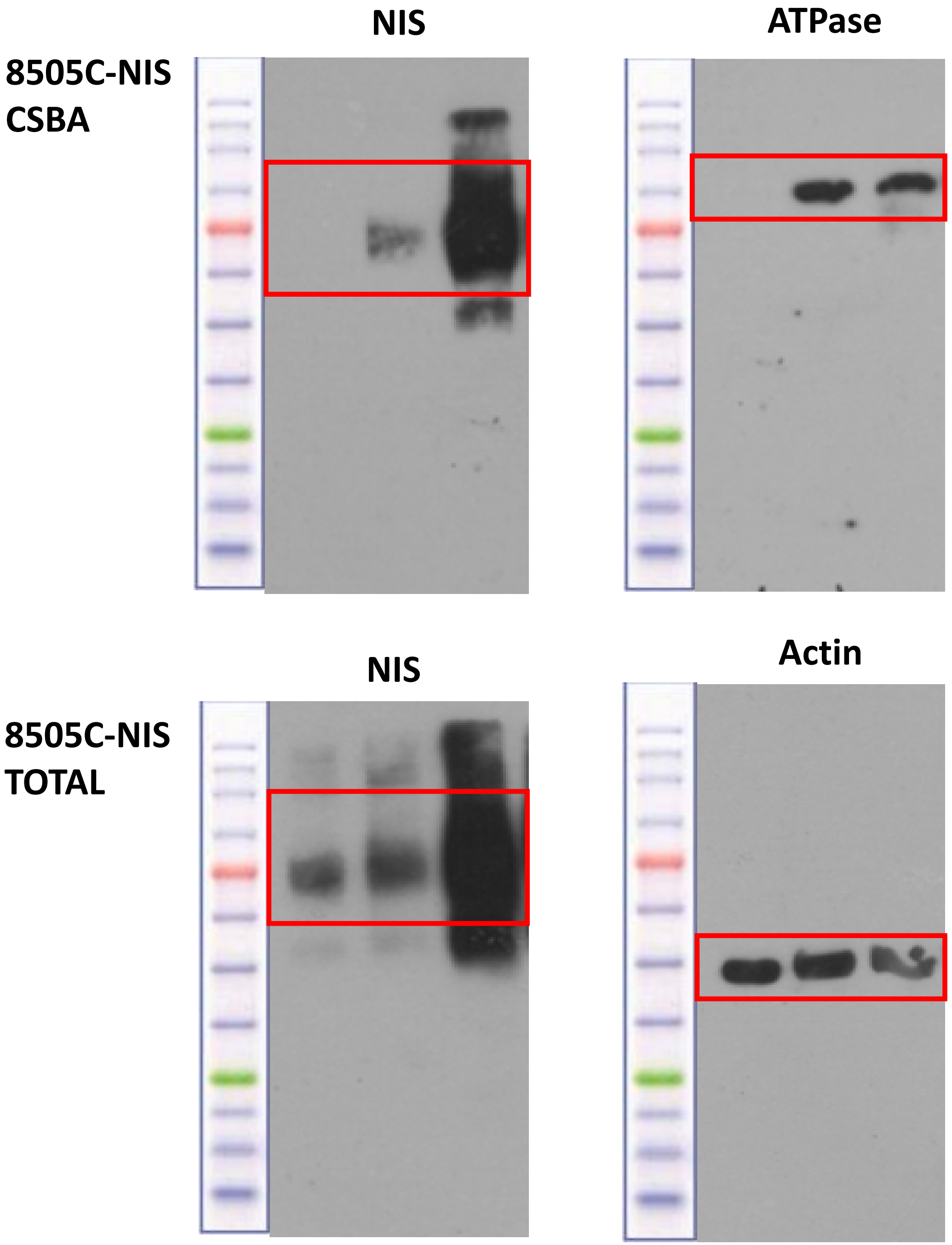

NIS
ATPase
8505C-NIS CSBA
NIS
Actin
8505C-NIS TOTAL

Figure 3g

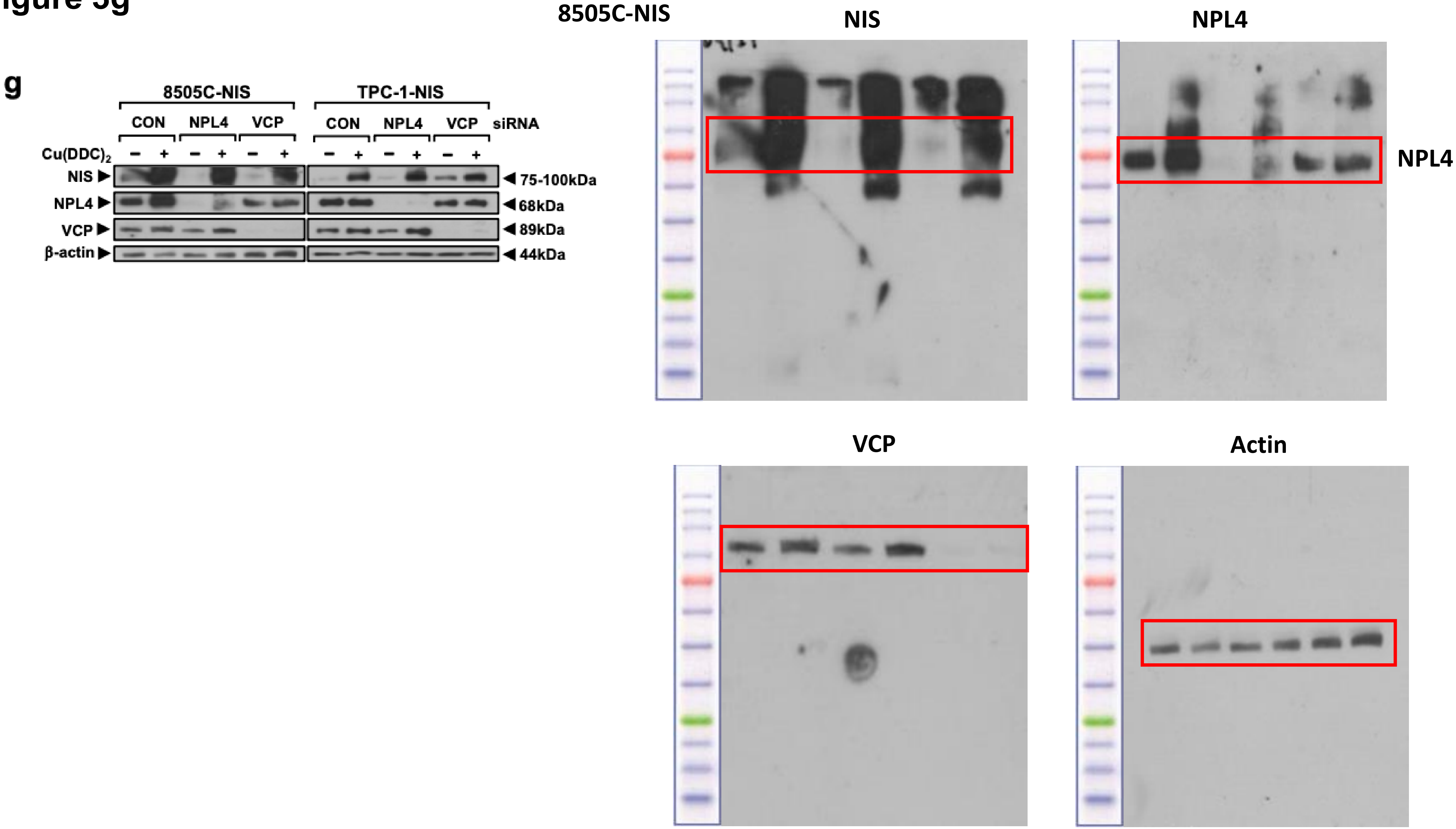
g
8505C-NIS
TPC-1-NIS
CON
NPL4
VCP
siRNA
Cu(DDC)2
NIS
NPL4
VCP
β-actin
75-100kDa
68kDa
89kDa
44kDa
8505C-NIS
NIS
NPL4
NPL4
VCP
Actin

Figure 3g

TPC1-NIS

g

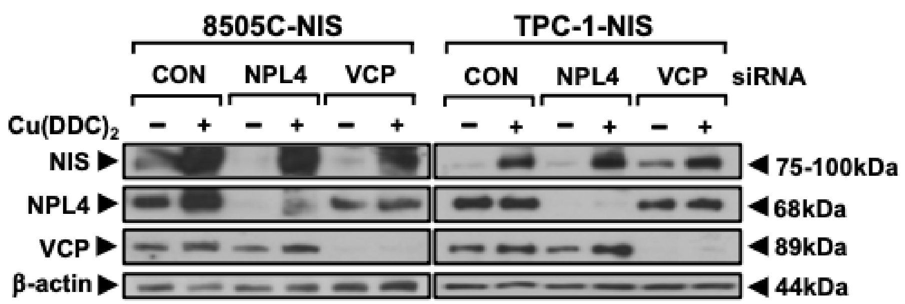

NIS

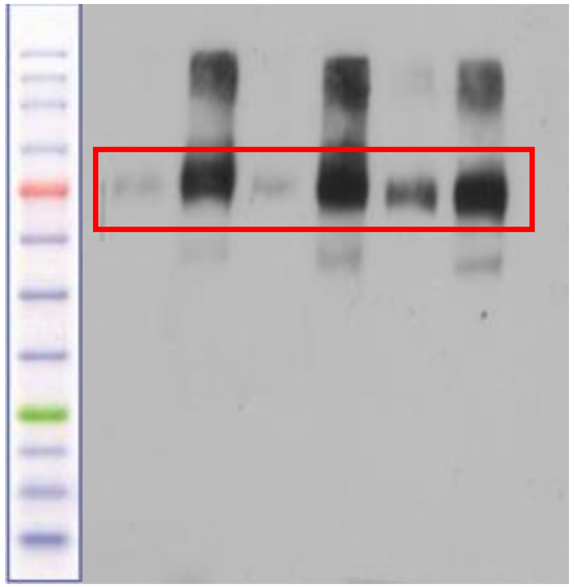

NPL4

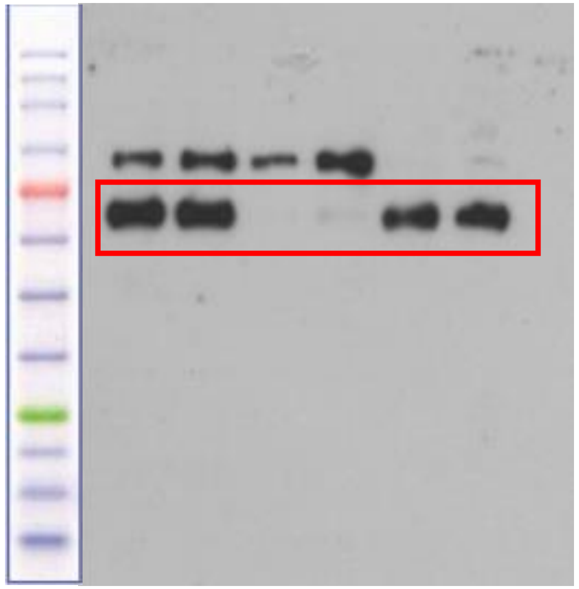

VCP bleed through when probed for NPL4

VCP

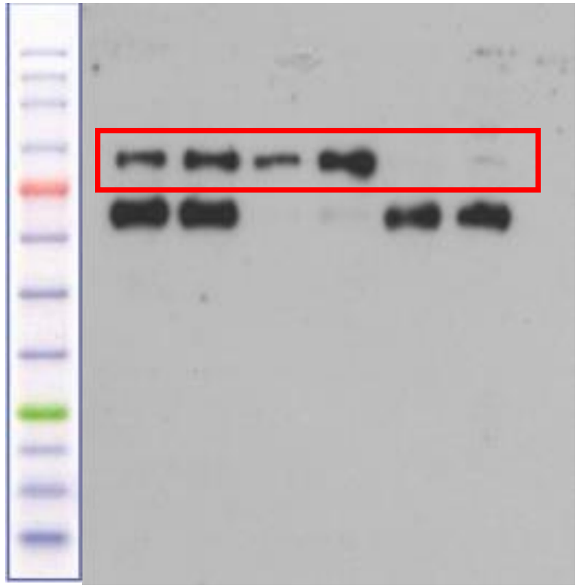

Actin

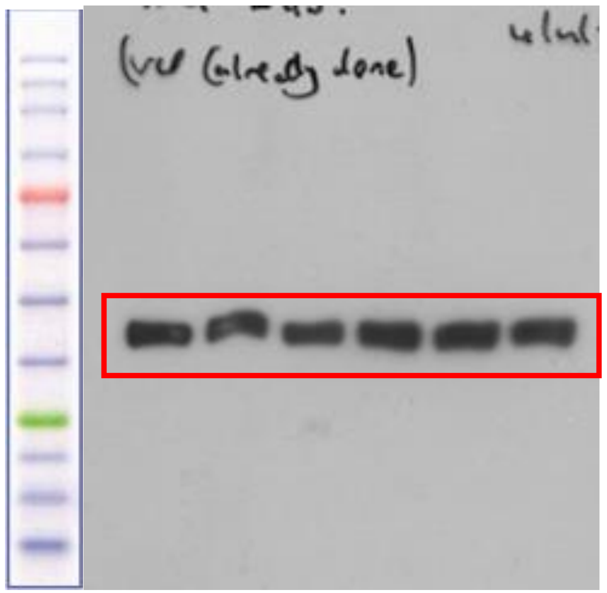

Figure 6c

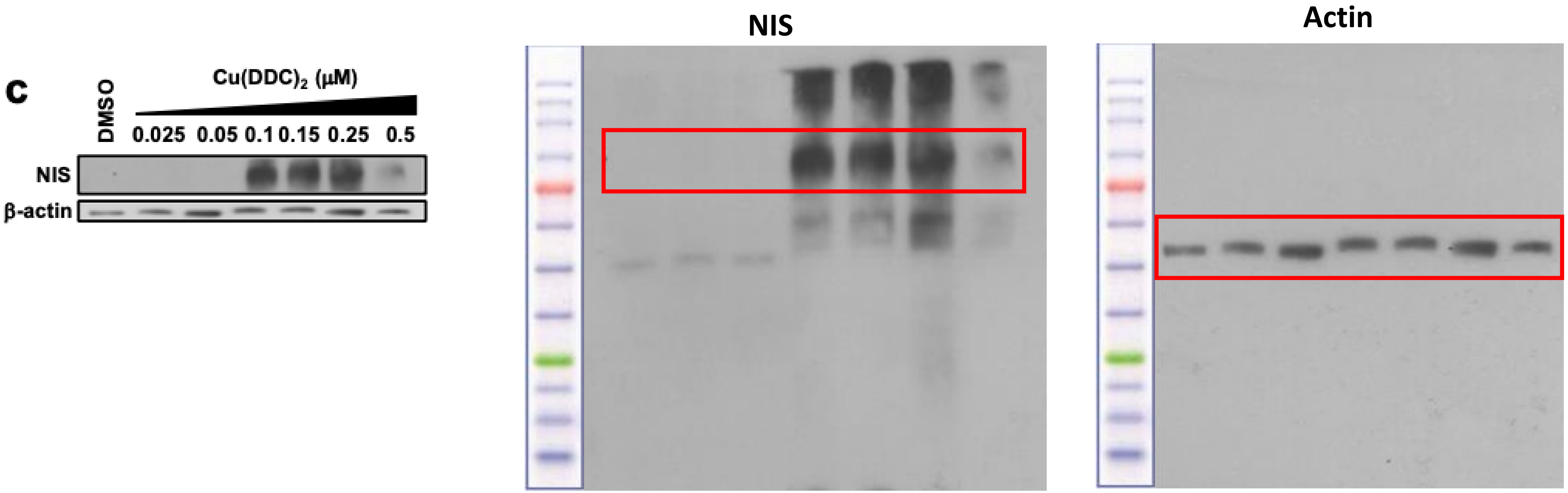

Supp Figure S5

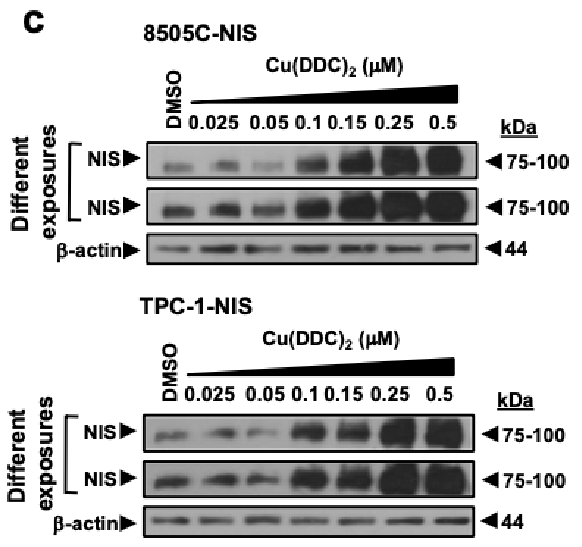

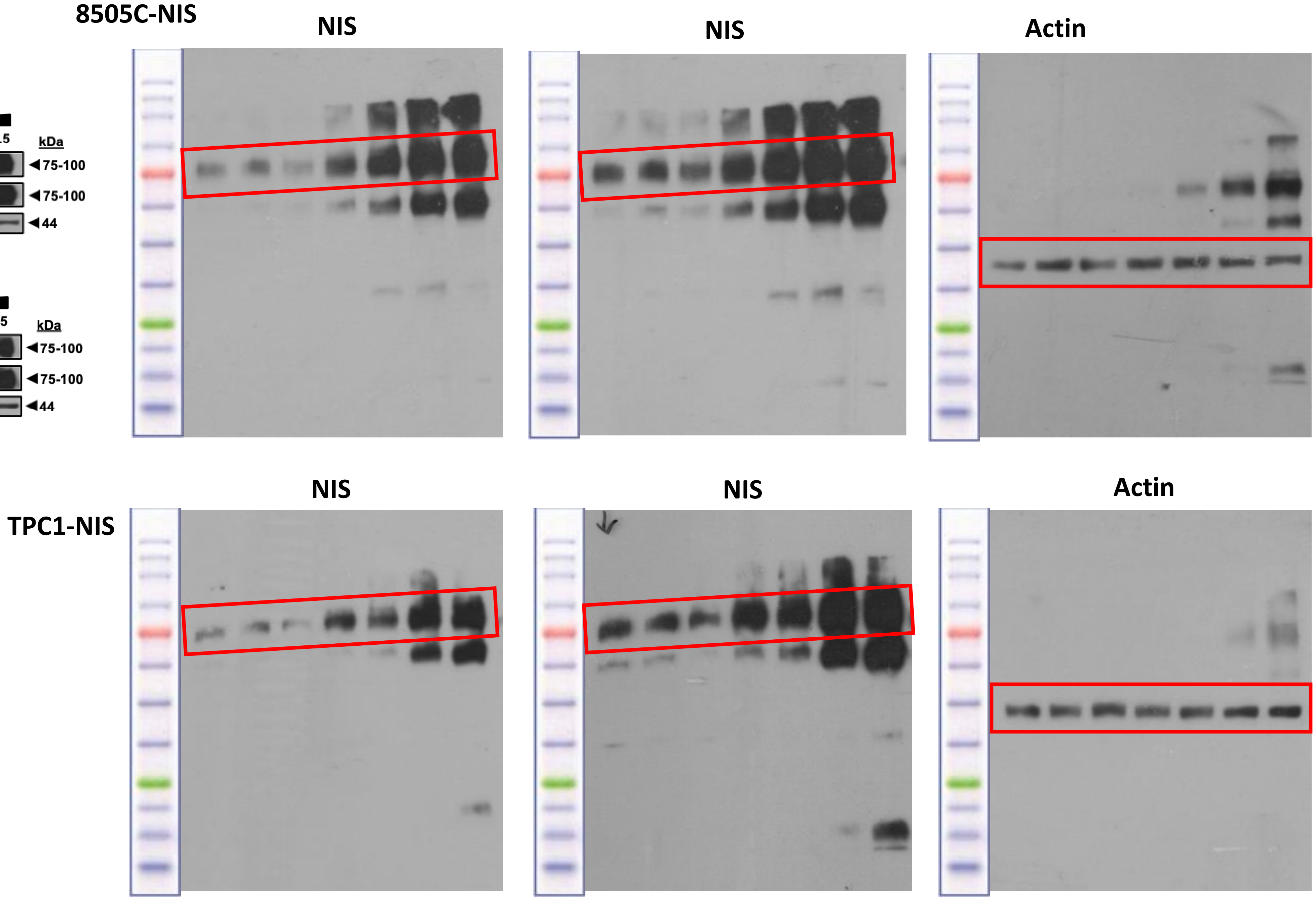

Supp Figure S5

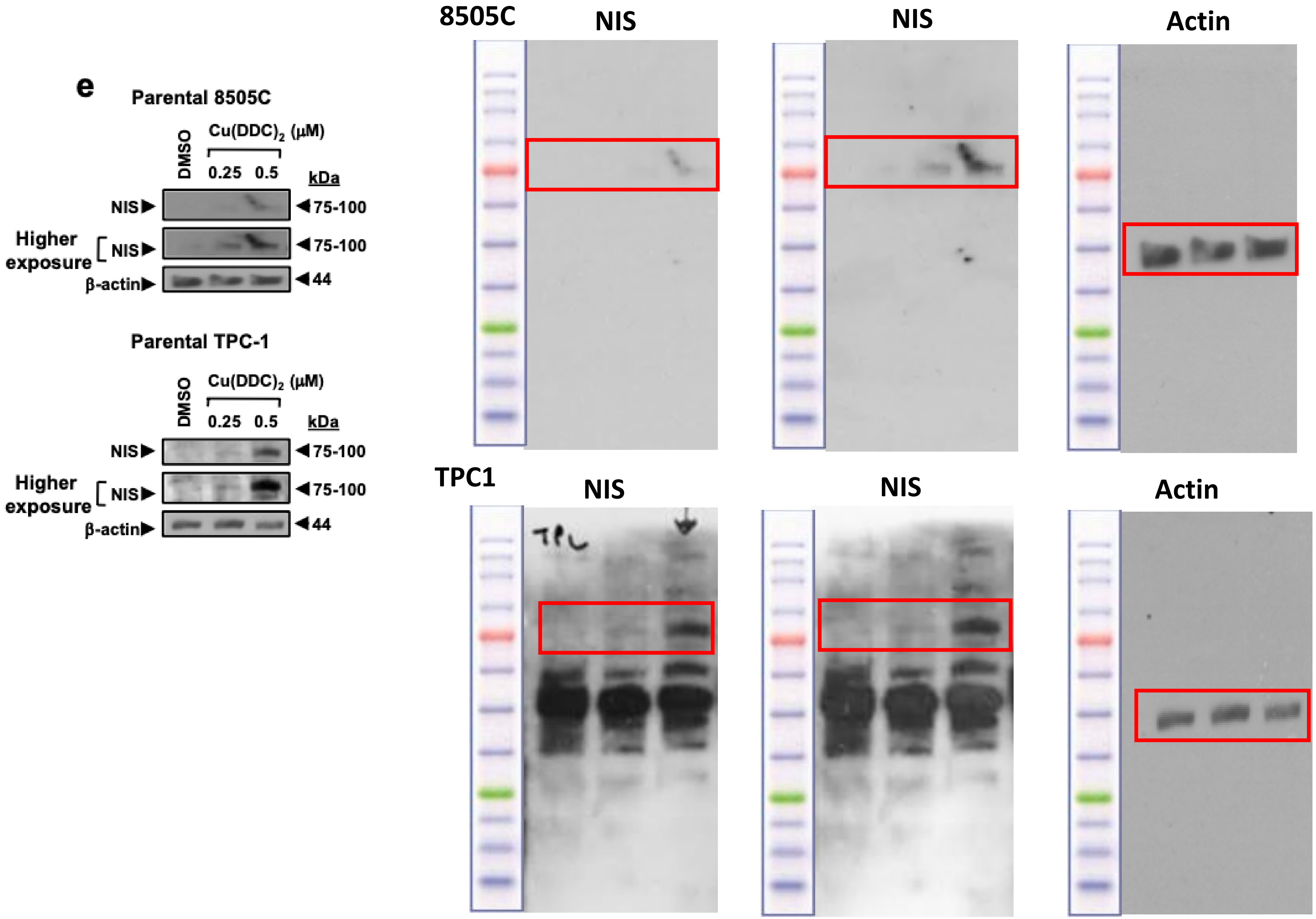

Supp Figure S5

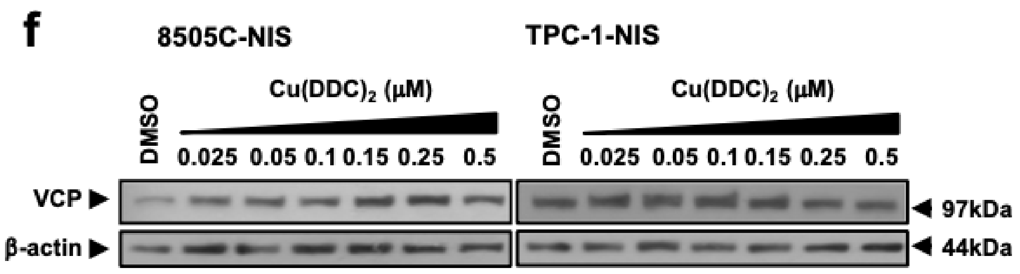

8505C-NIS

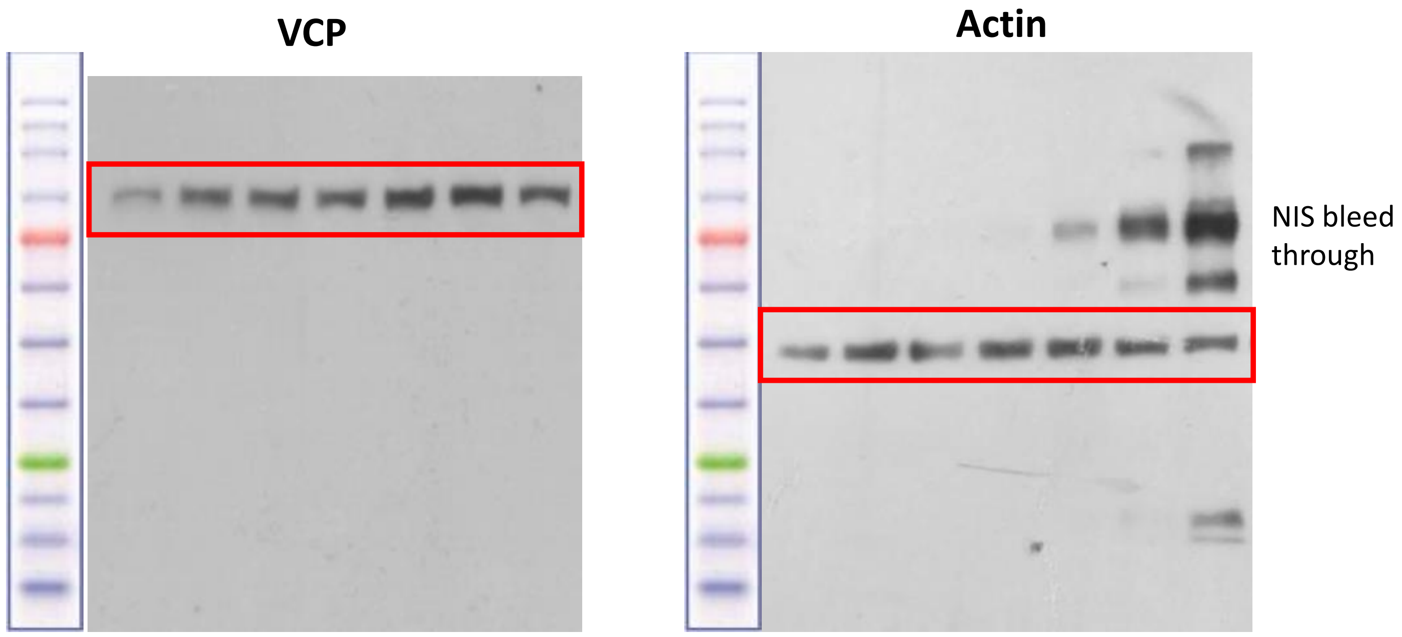

Supp Figure S5

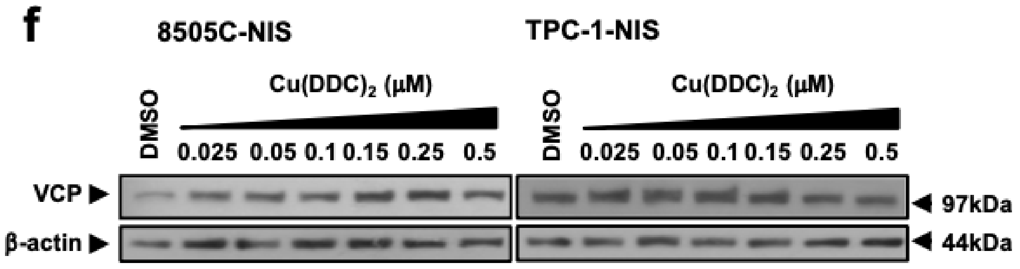

TPC1-NIS

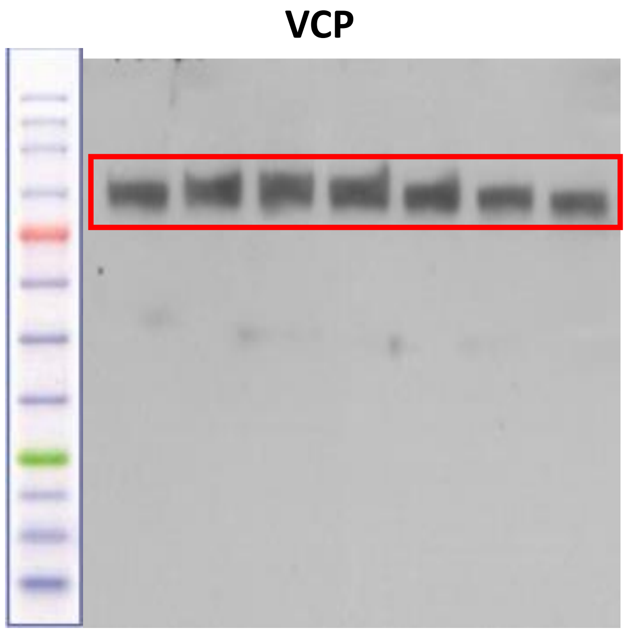

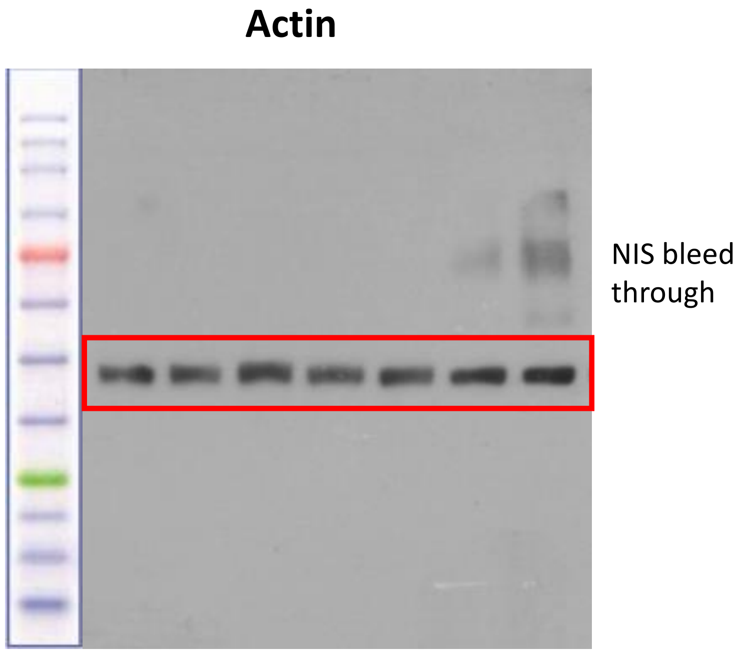

Supp Figure S6

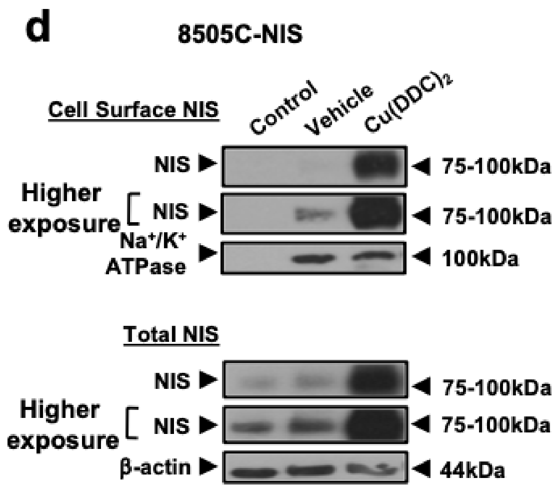

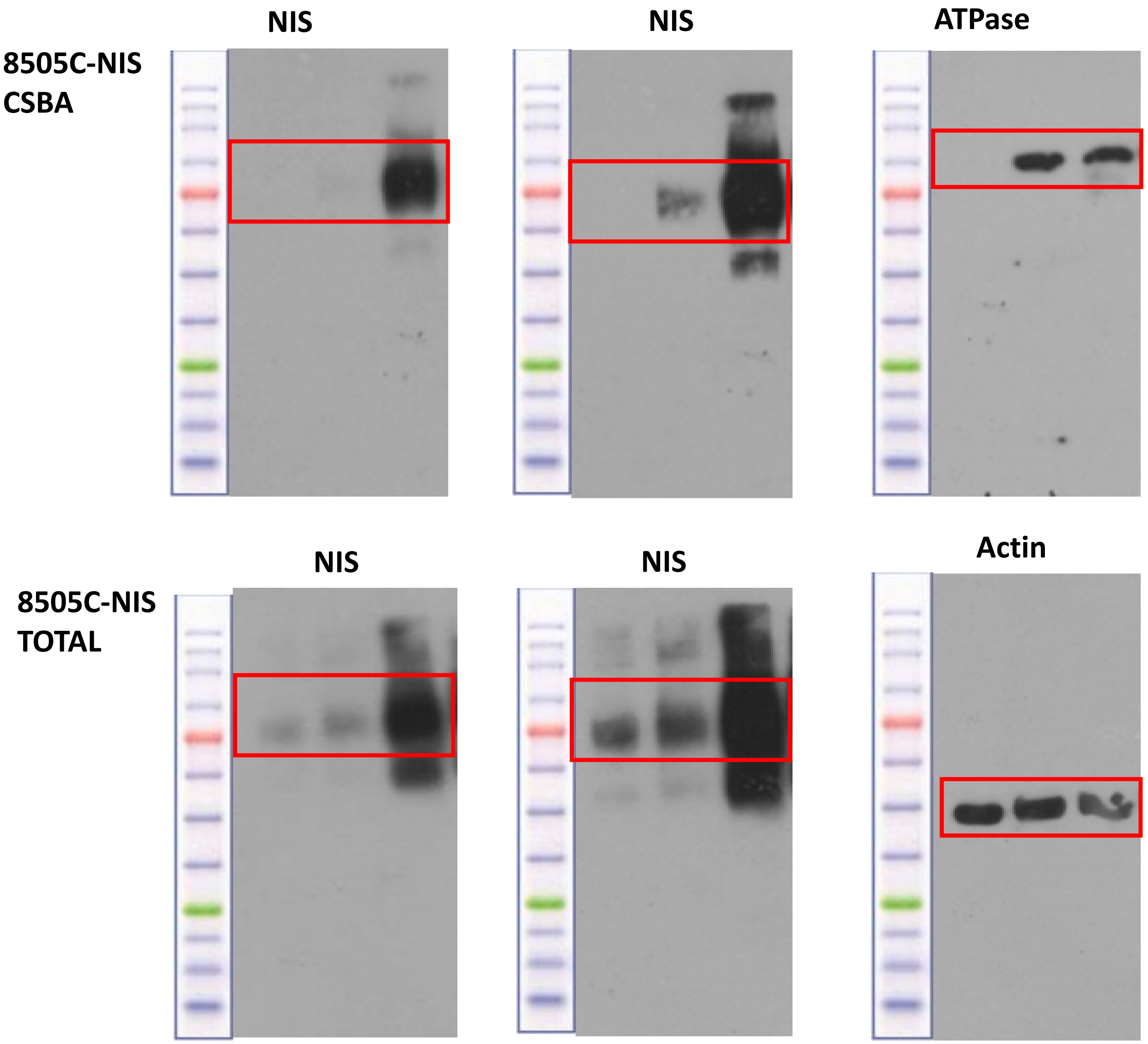

Supp Figure S6

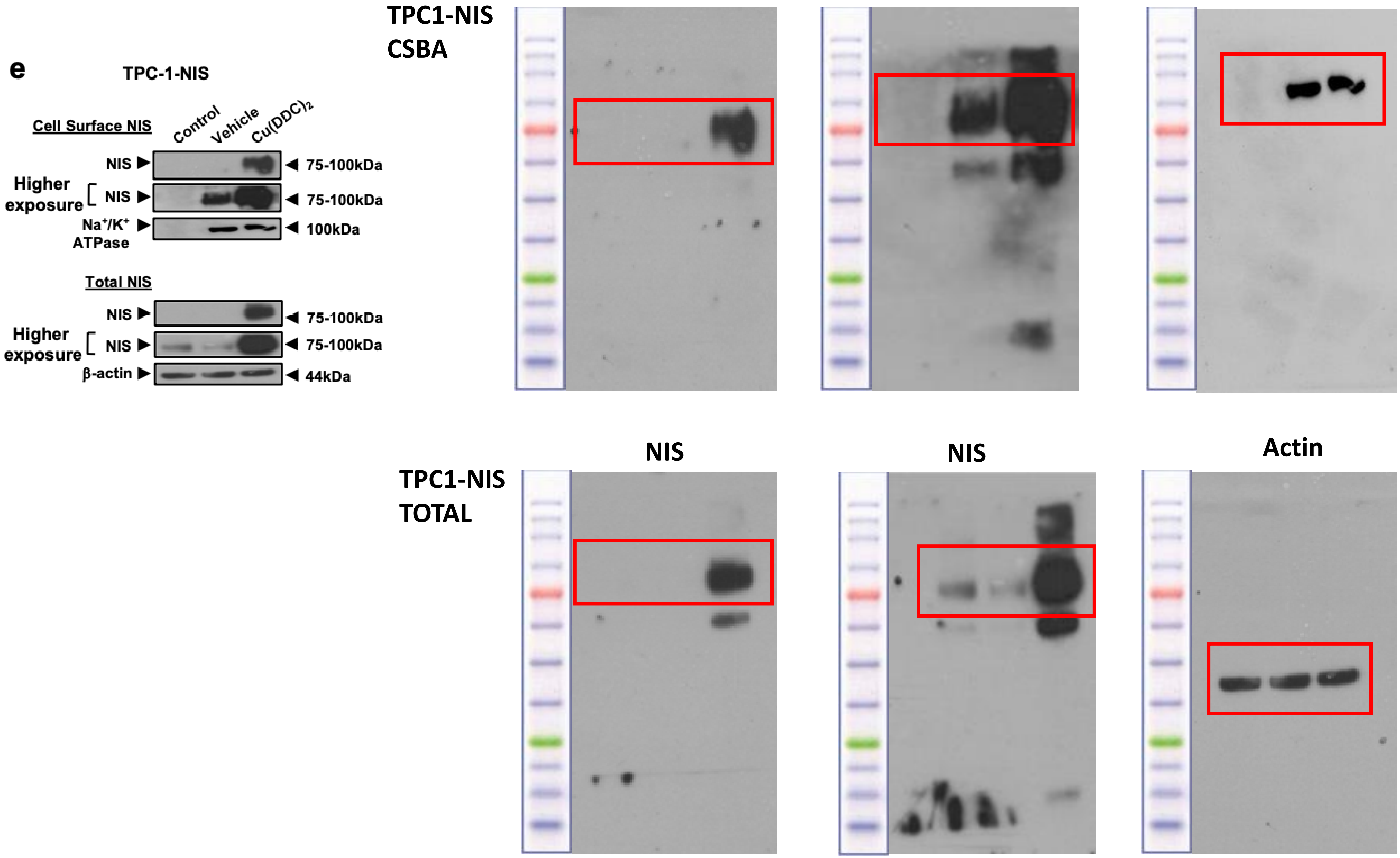

Supp Figure S8

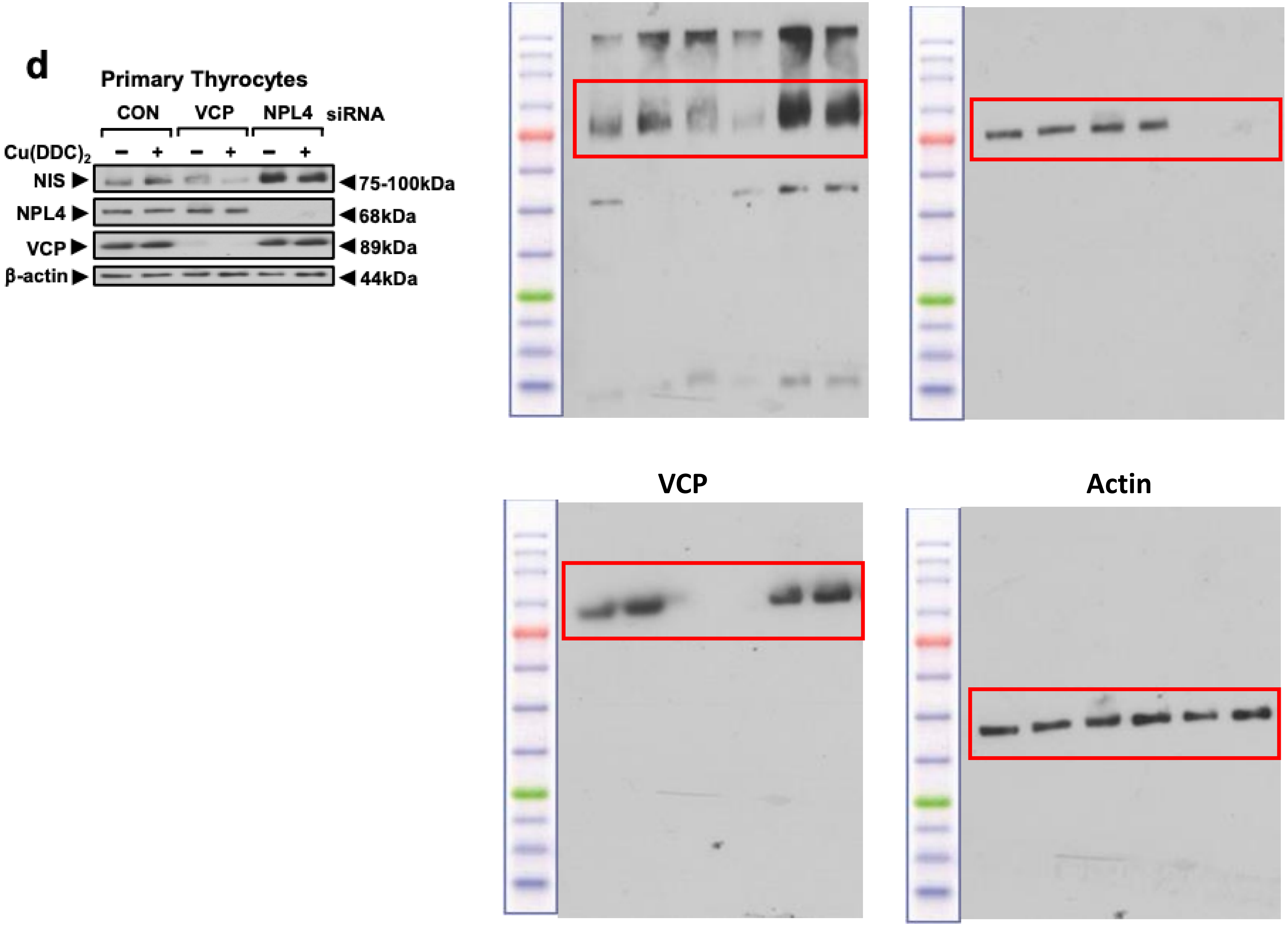

Supp Figure S19

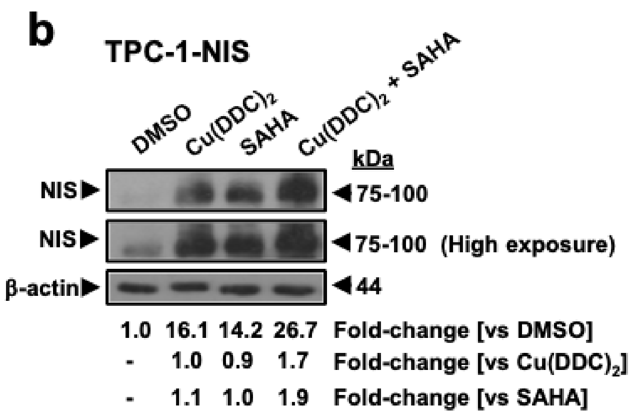

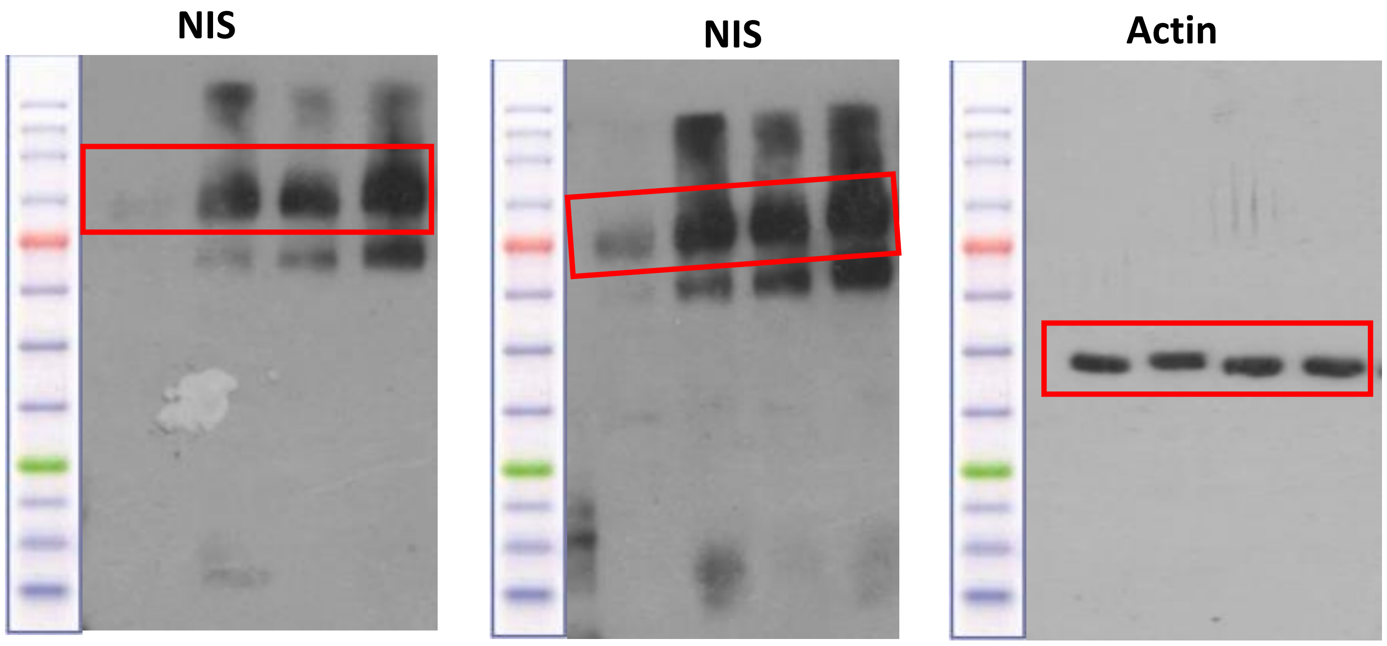

BLUeye pre-stained protein ladder

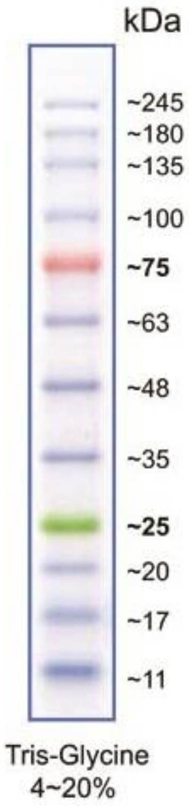

Supplement: Supplemental Western Blots [file mmc2.pdf]
