# Supplementary material for: Disulfiram metabolite Cu(DDC)2 enhances radionuclide uptake in vivo revealing insights into tumoural ablation resistance
Source: eBioMedicine. 2026 Feb 11;125:106165. doi: 10.1016/j.ebiom.2026.106165 (PMC12917385; doi:10.1016/j.ebiom.2026.106165)
Supplement: Supplementary Information [file mmc3.docx]

**Supplementary Information**

**Disulfiram metabolite Cu(DDC)_2_ enhances radionuclide uptake *in vivo* revealing insights into tumoural ablation resistance**

Katie Brookes,^a^ Jessica S. Fear,^a^ Caitlin E.M. Thornton,^a^ Ling Zha,^a^ Jana Kim,^b^ Benjamin Small,^c,d^ Sarinya Wongsanit,^a^ Hannah R. Nieto,^a^ Holly Adcock,^e^ Adam Jones,^b^ Truc T. Pham,^b^ Giovanni Bottegoni,^f,g^ Liam R. Cox,^e^ Vinodh Kannappan,^c,d^ Weiguang Wang,^c,d^ Caroline M. Gorvin,^a^ Daniel G. Stover,^h^ Christine Spitzweg,^i,j^ Sissy Jhiang,^h^ Matthew D. Ringel,^h^ Moray J. Campbell,^k^ Kavitha Sunassee,^b^ Philip J. Blower,^b^ Kristien Boelaert,^l^ Vicki E. Smith,^1^ Martin L. Read,^a,↑^ and Christopher J. McCabe,^a,↑^

^a^Metabolism and Systems Science, College of Medicine and Health, University of Birmingham, Birmingham, UK

^b^School of Biomedical Engineering & Imaging Sciences, King’s College London, London, UK

^c^Research Institute in Healthcare Science, Faculty of Science and Engineering, University of Wolverhampton, Wolverhampton, UK

^d^Disulfican Ltd, University of Wolverhampton Science Park, Wolverhampton, UK

^e^School of Chemistry, University of Birmingham, Birmingham, UK

^f^Institute of Clinical Sciences, University of Birmingham, Birmingham, UK

^g^Università degli Studi di Urbino Carlo Bo, Urbino, Italy

^h^Division of Endocrinology, Diabetes, and Metabolism and Cancer Biology Program, The Ohio State University College of Medicine and Comprehensive Cancer Center, Columbus, Ohio, USA

^i^Department of Internal Medicine IV, University Hospital of Munich, LMU Munich, Munich, Germany

^j^Division Endocrinology, Diabetes, Metabolism and Nutrition, Mayo Clinic, Rochester, MN, USA

^k^Division of Cancer Biology, Cedars Sinai Cancer, Los Angeles, USA

^l^Institute of Applied Health Research, University of Birmingham, Birmingham, UK

^↑^C J McCabe and M L Read contributed equally as senior authors.

**Corresponding authors**: Christopher J. McCabe, Metabolism and Systems Science, College of Medicine and Health, University of Birmingham, Birmingham, B15 2TH, UK. Email: mccabcjz@bham.ac.uk; Tel.: +44 (0) 121 415 8713. Martin L. Read, Metabolism and Systems Science, College of Medicine and Health, University of Birmingham, Birmingham, B15 2TH, UK. Email: m.l.read.20@bham.ac.uk. Tel.: +44 (0) 121 414 2764.

This PDF contains: (i) Key Resources, (ii) Supplementary Methods, (iii) Supplementary References

**(i) Key Resources**

| **REAGENT or RESOURCE** | **SOURCE** | **IDENTIFIER** |
| --- | --- | --- |
| **Antibodies** |  |  |
| Rabbit polyclonal anti-VCP | Proteintech | Cat#10736-1-AP;  RRID:AB_2214635 |
| Rabbit polyclonal anti-NPL4 | Cell Signaling Technology | Cat#13489;  RRID: AB_2798232 |
| Rabbit polyclonal anti-NIS | Proteintech | Cat#24324-1-AP; RRID: AB_2879495 |
| Mouse monoclonal anti-HA | BioLegend | Cat#901502; RRID: AB_2565006 |
| Mouse monoclonal anti-β-actin | Sigma-Aldrich | Cat#A1978; RRID: AB_476692 |
| Goat polyclonal anti-rabbit immunoglobulins/ HRP | Agilent Technologies | Cat#P0448; RRID:AB_2617138 |
| Rabbit polyclonal anti-Na,K-ATPase | Cell Signaling Technology | Cat# 3010  RRID: AB_2060983 |
| Rabbit polyclonal anti-mouse immunoglobulins/ HRP | Agilent Technologies | Cat#P0260; RRID:AB_2636929 |
| Goat anti-Rabbit IgG secondary antibody, Alexa Fluor™ 555 | ThermoFisher Scientific | Cat#A-21428; RRID:AB_2535849 |
| Goat anti-Mouse IgG secondary antibody, Alexa Fluor™ 488 | ThermoFisher Scientific | Cat#A28175; RRID:AB_2536161 |
| **Bacterial and Virus Strains** |  |  |
| Subcloning efficiency^TM^ DH5α competent cells | ThermoFisher Scientific | Cat#18265017 |
| **Biological Samples** |  |  |
| Human thyroid tissue | Queen Elizabeth Hospital, Birmingham, UK | N/A |
| **Oligonucleotides** |  |  |
| ATF3 TaqMan® Gene Expression Assay (human) | ThermoFisher Scientific | Hs00231069_m1 |
| CREM TaqMan® Gene Expression Assay (human) | ThermoFisher Scientific | Hs01582003_g1 |
| FOS TaqMan® Gene Expression Assay (human) | ThermoFisher Scientific | Hs04194186_s1 |
| JUN TaqMan® Gene Expression Assay (human) | ThermoFisher Scientific | Hs01103582_s1 |
| PAX8 TaqMan® Gene Expression Assay (human) | ThermoFisher Scientific | Hs00247586_m1 |
| PPIA TaqMan® Gene Expression Assay (human) | ThermoFisher Scientific | Hs04194521_s1 |
| SLC5A5 TaqMan® Gene Expression Assay (human) | ThermoFisher Scientific | Hs00950358_m1 |
| SMAD3 TaqMan® Gene Expression Assay (human) | ThermoFisher Scientific | Hs00969210_m1 |
| TG TaqMan® Gene Expression Assay (human) | ThermoFisher Scientific | Hs00174974_m1 |
| TPO TaqMan® Gene Expression Assay (human) | ThermoFisher Scientific | Hs00892519_m1 |
| TSHR TaqMan® Gene Expression Assay (human) | ThermoFisher Scientific | Hs01053846_m1 |
| ACTB TaqMan® Gene Expression Assay (mouse) | ThermoFisher Scientific | Mm01205647_g1 |
| ATF3 TaqMan® Gene Expression Assay (mouse) | ThermoFisher Scientific | Mm00476033_m1 |
| CREB1 TaqMan® Gene Expression Assay (mouse) | ThermoFisher Scientific | Mm00501607_m1 |
| CREM TaqMan® Gene Expression Assay (mouse) | ThermoFisher Scientific | Mm04336053_g1 |
| FOS TaqMan® Gene Expression Assay (mouse) | ThermoFisher Scientific | Mm00487425_m1 |
| JUN TaqMan® Gene Expression Assay (mouse) | ThermoFisher Scientific | Mm07296811_s1 |
| NKX2-1 TaqMan® Gene Expression Assay (mouse) | ThermoFisher Scientific | Mm07296387_g1 |
| PAX8 TaqMan® Gene Expression Assay (mouse) | ThermoFisher Scientific | Mm00440623_m1 |
| SLC5A5 TaqMan® Gene Expression Assay (mouse) | ThermoFisher Scientific | Mm01351811_m1 |
| SMAD3 TaqMan® Gene Expression Assay (mouse) | ThermoFisher Scientific | Mm01170760_m1 |
| TG TaqMan® Gene Expression Assay (mouse) | ThermoFisher Scientific | Mm01200340_m1 |
| TPO TaqMan® Gene Expression Assay (mouse) | ThermoFisher Scientific | Mm00456355_m1 |
| ON-TARGETplus Non-targeting Pool siRNA | Horizon Discovery | Cat#D-001810-10-05 |
| ON-TARGETplus PAX8 siRNA, SMARTpool | Horizon Discovery | Cat#L-003778-00-0005 |
| CREM siRNA | Insight Biotechnology Limited | Cat#sc-37700 |
| ON-TARGETplus VCP siRNA, SMARTpool | Horizon Discovery | Cat#L-008727-00-0005 |
| ON-TARGETplus NPL0C4 siRNA, SMARTpool | Horizon Discovery | Cat#L-020796-01-0005 |
| **Recombinant DNA** |  |  |
| pcDNA3.1(+) | ThermoFisher Scientific | Cat#V79020 |
| pcDNA3.1-NIS-HA | Smith VE et al., 2009 | N/A |
| pcDNA3.1-NIS-SmBiT | Read ML et al., 2022 | N/A |
| pcDNA3.1-LgBiT-VCP | Read ML et al., 2022 | N/A |
| pcDNA3.1-RAB1-Venus | Kevin Pfleger’s lab  (University of Western Australia) | N/A |
| pcDNA3.1-RAB8-Venus | Kevin Pfleger’s lab  (University of Western Australia) | N/A |
| pcDNA3.1-KRAS-Venus | Nevin Lambert’s lab  (Georgia Regents University) | N/A |
| pcDNA3.1-RAB5-Venus | Nevin Lambert’s lab  (Georgia Regents University) | N/A |
| pcDNA3.1-RAB11-Venus | Nevin Lambert’s lab  (Georgia Regents University) | N/A |
| pcDNA3.1-NIS-Nluc | Read ML et al., 2024 | N/A |
| **Chemicals** |  |  |
| CB-5083 | This paper | N/A |
| CB-5339 | This paper | N/A |
| Clotrimazole | Sigma-Aldrich | Cat#C6019 |
| Clotrimazole analogues C1 to C25 | This paper | N/A |
| Copper gluconate | Sigma-Aldrich | Cat#527-09-3 |
| Cu(DDC)_2_ | Tokyo Chemical Industry | Cat#D0487 |
| Cu(DDC)_2_-albumin | This paper | N/A |
| Disulfiram | Sigma-Aldrich | Cat#86720 |
| Vorinostat (SAHA) | Stratech Scientific | Cat#S1047 |
| **Experimental Models: Cell Lines** |  |  |
| AU565 | Clare Davies’ lab (University of Birmingham) | RRID:CVCL_1074 |
| BCPAP | Rebecca Schweppe’s lab  (University of Colorado) | RRID:CVCL_0153 |
| HEK293 | European Collection of Authenticated Cell Cultures (ECACC) | Cat#85120602;  RRID: CVCL_0045 |
| HeLa | ECACC | Cat#93021013;  RRID: CVCL_0030 |
| L87-NIS | Christine Spitzweg’s lab  (University Hospital of Munich) | RRID: CVCL_6837  (parental) |
| MCF7 | Leibniz Institute  DSMZ-German Collection of  Microorganisms and Cell Cultures GmbH (DSMZ) | Cat#ACC115; RRID:CVCL_0031 |
| MDA-MB-231 | American Type  Culture Collection (ATCC) | Cat#ATCC-HTB-26;  RRID:CVCL_0062 |
| MDA-MB-231-NIS | Fletcher A et al. 2020 | N/A |
| SK-BR-3 | DSMZ | Cat#ACC736; RRID:CVCL_0033 |
| SUM52 | John Heath’s lab (University of Birmingham) | RRID:CVCL_3425 |
| SW1736 | Rebecca Schweppe’s lab  (University of Colorado) | RRID:CVCL_3883 |
| TPC-1 | Rebecca Schweppe’s lab  (University of Colorado) | RRID:CVCL_6298 |
| TPC-1-NIS | Read ML et al., 2022 | N/A |
| ZR751 | Clare Davies’ lab (University of Birmingham) | RRID:CVCL_0588 |
| 8505C | DSMZ | Cat#ACC219;  RRID: CVCL_1054 |
| 8505C-NIS | Read ML et al., 2024 | N/A |
| **Experimental Models: Organisms/Strains** |  |  |
| BALB/cAnNCrl (BALB/c) | Charles River | RRID:IMSR_CRL:028 |
| Tg-rtTA/tetO-BRAF^V600E^ | Matthew Ringel’s lab (Ohio State University) | doi: 10.1172/JCI46382 |
| NOD.Cg-Prkdc^scid^ Il2rg^tm1WjI^/SzJ (NSG) | Charles River | RRID:IMSR_JAX:005557 |
| **Deposited data** |  |  |
| Thyroid carcinoma (TCGA, Firehose Legacy) | https:www.cbioportal.org/study/summary?id=thca_tcga; | RRID:SCR_014555 |
| TCGA GDAC Firehose standard data – Thyroid Carcinoma (THCA) | Broad Institute of MIT and Harvard | https://doi.org/10.7908/C11G0KM9  http://firebrowse.org |
| TCGA THCA | https://portal.gdc.cancer.gov/projects/TCGA-THCA | RRID:SCR_014514 |
| GSE33630 | https://www.ncbi.nlm.nih.gov/geo/geo2r/?acc=GSE33630 | N/A |
| Sodium/iodide cotransporter structure | AlphaFold Protein Structure Database | https://alphafold.ebi.ac.uk/entry/Q92911 |
| VCP structure | https://www.rcsb.org/ | doi.org/10.2210/pdb7K59/pdb |
| **Software** |  |  |
| GraphPad Prism Version 10.4 | GraphPad Software | RRID:SCR_002798 |
| Excel 2016 | Microsoft | RRID:SCR_016137 |
| PowerPoint 2016 | Microsoft | RRID:SCR_023631 |
| EndNote 20 | Clarivate | RRID:SCR_014001 |
| SPSS Statistics Version 29 | IBM | RRID:SCR_002865 |
| R Project for Statistical Computing | http://www.r-project.org/ | RRID:SCR_001905 |
| EdgeR | http://bioconductor.org/packages/edgeR/ | RRID:SCR_012802 |
| GEO2R | National Center for Biotechnology Information | RRID:SCR_016569 |
| BioRender | http://biorender.com | RRID:SCR_018361 |
| ImageJ | National Institutes of Health | RRID:SCR_003070 |
| Morpheus | Broad Institute | RRID:SCR_017386 |
| DAVID Bioinformatics | National Institutes of Health | RRID:SCR_001881 |
| ToppGene Suite | Cincinnati Children's Hospital Medical Center | RRID:SCR_005726 |
| XLSTAT | Lumivero | RRID:SCR_016299 |
| VivoQuant Preclinical Imaging Software | InviCRO | https://www.vivoquant.com/ |
| RandoMice | Leiden University Medical Center, Leiden, the Netherlands | https://github.com/RvE54/RandoMice?tab=readme-ov-file |
| DynaVenn | Chair for Clinical Bioinformatics at Saarland University | https://ccb-compute.cs.uni-saarland.de/dynavenn |

**(ii) Supplementary Methods**

**Development and validation of a multigene riskscore classifier to predict recurrence in RAI-treated PTC**

Recurrence in PTC is associated with increased mortality.^1^ It is therefore anticipated that earlier prediction of PTC recurrence following RAI treatment will impact favourably on patient outcome. Despite a diverse range of gene classifiers being reported for thyroid cancer^2,3,4^, the lack of training such signatures to account for the impact of treatment strategies will likely weaken or distort their usefulness in the clinical setting. Therefore, our rationale was to extrapolate mechanistic insights based on clinically relevant transcriptional and proteostatic factors linked to radionuclide uptake to develop a prognostic riskscore for PTC recurrence in patients treated with RAI. We envisage that the intended purpose of our riskscore classifier is to be part of the dynamic risk stratification that occurs for patients who have undergone a total thyroidectomy and RAI treatment, and who may benefit from intensified surveillance and additional therapy. Its intended users are oncologists and multidisciplinary care teams.

Our objectives were to describe the development and validation of a prediction model for recurrence in RAI-treated PTC, and also evaluate its applicability in the entire TCGA THCA. We used TCGA THCA as it is the largest publicly available PTC dataset with extensive molecular tumour profiling and correlation with survival indicators. Recent studies have indicated that white patients are significantly overrepresented in TCGA compared to the U.S population, indicating that our findings from TCGA may not be generalisable to minority populations.^5^ The low sample size for minority groups in TCGA THCA might therefore limit the statistical power needed to identify less common but potentially useful prediction models specific to these groups.

Project wide the sample recruitment period for TCGA was 2006-2013 and the data freeze for key TCGA THCA analysis was 14/7/2013. Overall, TCGA used 161 tissue source sites and TCGA THCA samples were drawn from a subset of them. TCGA THCA cases consisted of histologically confirmed, treatment naïve primary thyroid carcinomas and available clinical annotation. All tissue specimens met TCGA quality control criteria and were collected under local Institutional Review Board (IRB) approved protocols with informed consent. A subset of patients with PTC received postoperative RAI therapy. Data on tissue source sites and administered RAI doses are documented (NCI Genomic Data Commons (GDC; portal.gdc.cancer.gov/). TCGA THCA is a publicly available observational dataset and has not been prospectively registered as a clinical trial. No study protocols for TCGA-THCA have been posted. Clinical case quality control and enrollment forms are available (NCI Genomic Data Commons (GDC; portal.gdc.cancer.gov/). Sex data for TCGA THCA samples was obtained from clinical records at the submitting institutions and validated using sex chromosome–based genomic inference as part of TCGA quality control procedures.

Sample size was determined by the number of TCGA THCA cases with available molecular data, clinical annotation, and follow-up information for survival analysis. No discrimination in data inclusion was made based on personal attributes such as race, sex and age. For development of riskscore classifiers we used 137 cases characterised as BRAF-like RAI-treated PTC, whereas for validation in larger TCGA THCA cohorts we used RAI-treated PTC (n = 256), BRAF-like PTC (n = 261) and the entire TCGA THCA (n = 488). Some patient data in the BRAF-like, RAI-treated PTC (n = 20), RAI-treated PTC (n = 45), BRAF-like PTC (n = 48) and entire THCA THCA (n = 89) were not included in univariate and multivariate analysis due to missing clinical variables, including disease stage, risk group and outcome event, as well as incomplete T and N staging data. We limited this approach to univariate and multivariate analyses to avoid potential selection bias (Fig. 5m and n, Supplementary Tables S4-S6).

The rationale for constructing a multigene riskscore classifier was due to the anticipated gains of effect size weighting (i.e. integrating the combined prognostic effects of multiple classifiers into a single quantitative index), and improved model stability (i.e. more robust to noise than individual classifiers). In addition, stratification using a riskscore was expected to improve model interpretability and facilitate clinical decision making via patients being stratified into high and low risk groups. We applied the machine learning algorithm Least Absolute Shrinkage and Selection Operator (LASSO) using XLSTAT software (Addinsoft) to construct a prognostic model using transcriptional and proteostatic factors to avoid overfitting.^6^ The prognostic value of riskscore classifiers was then evaluated using Kaplan–Meier survival analysis and log-rank testing to assess risk stratification. Disease-free survival (DFS) was defined as the time from initial treatment to the first reported recurrence. Patients without recurrence at last follow-up were censored at the last contact date. Cumulative event plots are given for all classifiers (Supplementary Figure 18) showing the total number of outcome events over time, providing context for the Kaplan–Meier survival curves and timing of events for each stratified group.

Univariable and multivariable Cox proportional hazards models were used to assess the independent prognostic value of each multigene riskscore classifier after adjusting for clinical covariates, including age, sex, disease stage and risk group. Thresholds (cut-off values) for riskscore stratification using multigene riskscore classifiers are outlined in Supplementary Figure S17. We have also reported on the key characteristics between the BRAF-like, RAI-treated PTC and larger PTC datasets, including age at diagnosis, sex, disease stage, risk group, dual TF+VCP proteostasis riskscore classifier and recurrence events (Supplementary Table S4). The distributions of demographics in larger PTC datasets were overall comparable with the BRAF-like RAI-treated PTC dataset. Interestingly, a difference was indicated in the patient risk group but this pattern was not evident in other categories related to outcome.

Although we used TCGA THCA data that had already been preprocessed and normalised using standardised pipelines (i.e. FireBrowse (firebrowse.org)), data quality was assessed prior to construction of prediction models by eliminating samples with excessive missing values, implausible distributions or presence of outliers. Samples failing quality thresholds were excluded from model prediction. Our prediction model requires minimal user interaction beyond supplying appropriately preprocessed TCGA-format data and is intended for users with basic bioinformatics and computational expertise. No manual preprocessing or model tuning is required.

**(iii) Supplementary References**

1. Nieto HR, Thornton CEM, Brookes K, et al. Recurrence of Papillary Thyroid Cancer: A Systematic Appraisal of Risk Factors. *J Clin Endocrinol Metab.* 2022;107(5):1392-1406.

2. Lin P, Guo YN, Shi L, et al. Development of a prognostic index based on an immunogenomic landscape analysis of papillary thyroid cancer. *Aging.* 2019;**11**(2):480-500.

3. Ren H, Liu X, Li F, He X, Zhao N. Identification of a Six Gene Prognosis Signature for Papillary Thyroid Cancer Using Multi-Omics Methods and Bioinformatics Analysis. *Front Oncol.* 2021;11:624421.

4. Wang Y, Yang J, Chen S, Wang W, Teng L. Identification and Validation of a Prognostic Signature for Thyroid Cancer Based on Ferroptosis-Related Genes. *Genes.* 2022;13(6).

5. Barseghyan L, Chan S, Yamauchi CR, et al. Unraveling Racial Disparities in Papillary Thyroid Cancer: A Comparative Bulk RNA-Sequencing Gene Expression Analysis. *Curr Oncol.* 2025;32(6).

6. Tibshirani, R. Regression Shrinkage and Selection via the Lasso. *Journal of the Royal Statistical Society: Series B*. 1996;58(1), 267–288.
